# Supplementary material for: Comparing the levelized cost of electric vehicle charging options in Europe
Source: Nat Commun. 2022 Sep 8;13:5277. doi: 10.1038/s41467-022-32835-7 (PMC9458728; doi:10.1038/s41467-022-32835-7)
Supplement: Supplementary file 1 — Supplementary Information [file 41467_2022_32835_MOESM1_ESM.pdf]

Supplementary Information – Comparing the levelized cost of electric vehicle charging options in Europe

Lukas Lanz[1], Bessie Noll[1]*, Tobias S. Schmidt[1,2], Bjarne Steffen[2,3]*

[1] Energy and Technology Policy Group, Swiss Federal Institute of Technology, ETH Zurich, Clausiusstrasse 37, 8092 Zurich, Switzerland
[2] Institute of Science, Technology and Policy, Swiss Federal Institute of Technology, ETH Zurich, 8092 Zurich, Switzerland
[3] Climate Finance and Policy Group, Swiss Federal Institute of Technology, ETH Zurich, Clausiusstrasse 37, 8092 Zurich, Switzerland

* Correspondence: bessie.noll@gess.ethz.ch, bjarne.steffen@gess.ethz.ch

Contents

Supplementary Methods 3

Supplementary Methods 1: Modeling framework 3

Supplementary Methods 2: Technical implementation of LCOC model 4

Supplementary Methods 3: Components of LCOC 5

Supplementary Methods 4: Electricity cost of residential charging sites with solar rooftop PV 6

Supplementary Methods 5: Input data of LCOC parameters and data processing 10

Supplementary Methods 6: Auxiliary country-specific input data 17

Supplementary Methods 7: Auxiliary input data of European grid electricity prices 19

Supplementary Methods 8: Estimation of average Swiss electricity prices 21

Supplementary Methods 9: Conversion rates 22

Supplementary Methods 10: Additional data on user profiles 23

Supplementary Methods 11: Interviewees 25

Supplementary Discussion 26

Supplementary Discussion 1: General sensitivity analysis 26

Supplementary Discussion 2: Impact of electricity price variability 39

Supplementary Tables: Additional Results 48

Supplementary Figures: Additional Results 50

Supplementary References 75

Supplementary Methods 1: Modeling framework

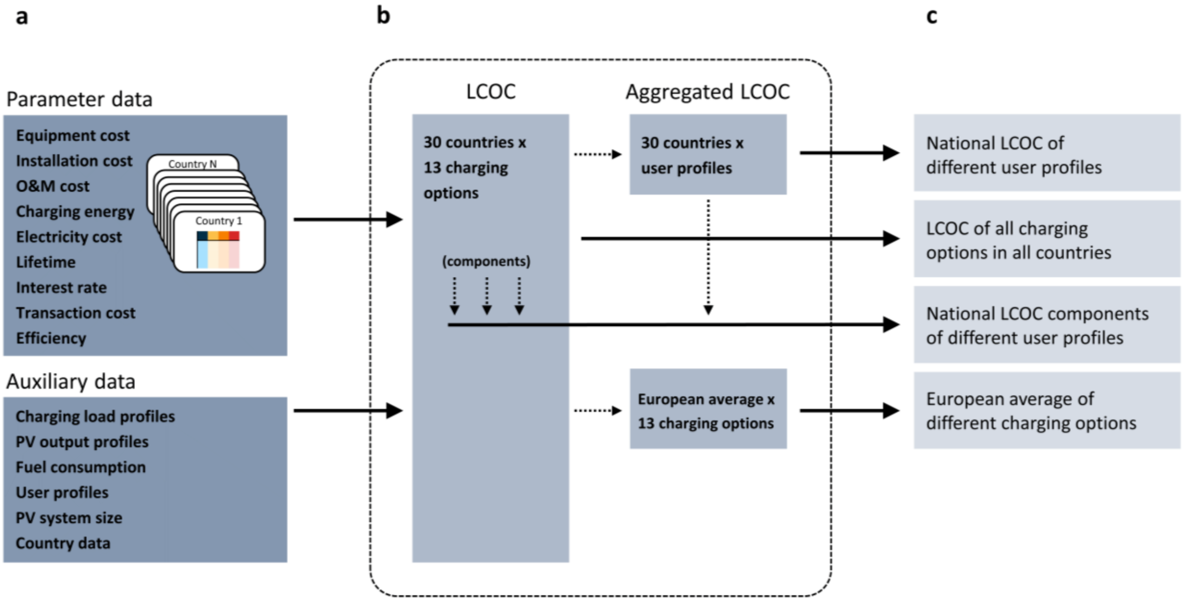

Supplementary Fig. 1. Framework of the LCOC model (own illustration). Modeling framework (own illustration). **a** Input data covering all parameters in the LCOC equation, as well as diverse auxiliary data, is fed to the model for every country and every charging option. **b** In the LCOC Model different components of the LCOC equation are evaluated and summed up for every country and charging option separately, resulting in 390 LCOC data points (30 countries x 13 charging options) (see Supplementary Fig. 2 for more detail). **c** Output data is then organized for plotting and analysis. The LCOC values are then aggregated by the user profiles to obtain national (country-specific) costs of typical charging behavior to get a European average cost.

Supplementary Methods 2: Technical implementation of LCOC model

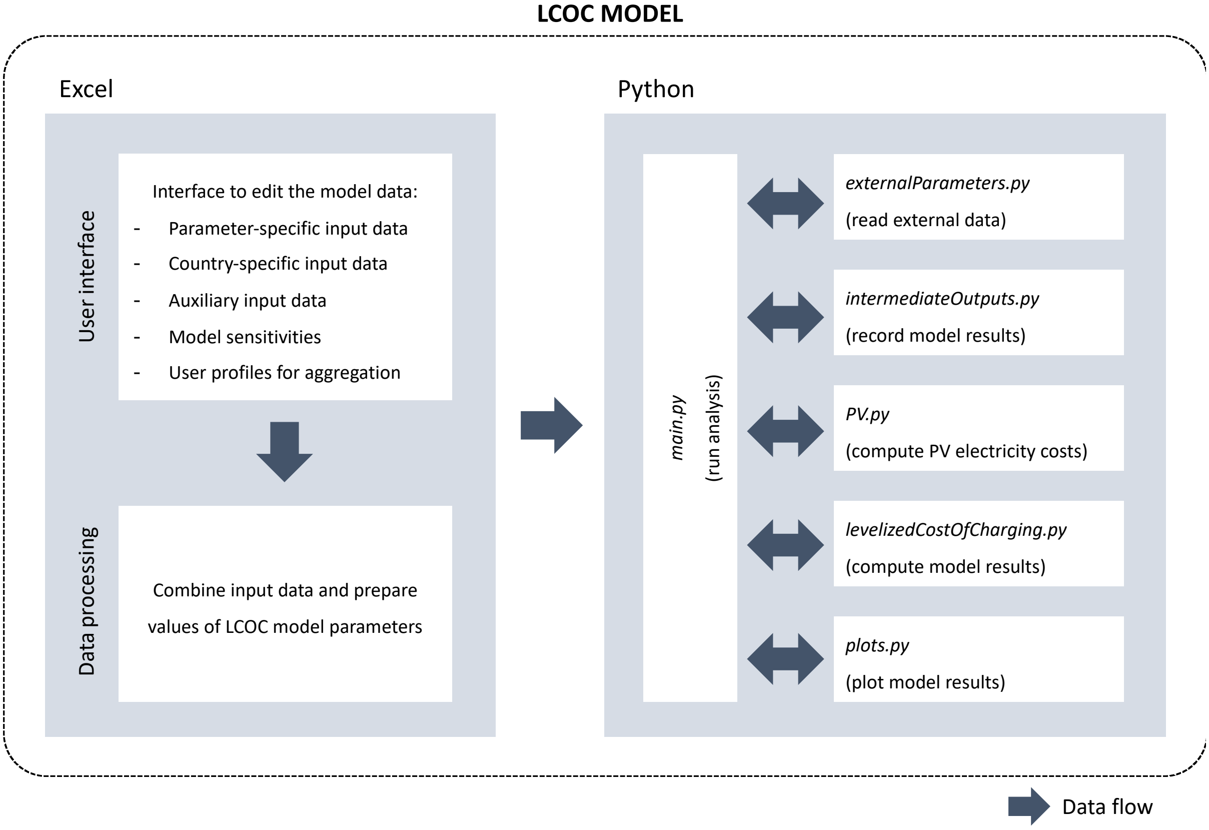

Supplementary Fig. 2. Technical implementation of the LCOC model (own illustration). Arrows describe dataflow and interactions of the model parts. The LCOC model is divided into two interrelated parts – an Excel spreadsheet which acts as a user interface and preprocesses the data and a Python model consisting of interacting modules. The blue arrows depict the data flow. The Python model reads in the data processing Excel files which take and reorganize data directly from the user interface Excel files. The Python model is comprised of six modules that call and interact with each other. To run the model, only the module *main.py* is called. The full Python model is attached as a supplementary code file (see Supplementary Software 1).

Supplementary Methods 3: Components of LCOC

In order to understand the drivers behind the results of this study, the model computes the LCOC equation component by component. The levelized cost of the single components are defined in Supplementary Equations (1a) to (1j).

$$\mathrm{LCOC_{equipment}}[€\ \mathrm{kWh^{-1}}] = C_{\mathrm{equipment}}\ [€] \Big/ \sum_{t=1}^{T} \frac{\text{Charging Energy}_t\ [\mathrm{kWh\ year^{-1}}]}{(1+i)^t}$$ Supplementary Equation (1a)

$$\mathrm{LCOC_{installation}}[€\ \mathrm{kWh^{-1}}] = C_{\mathrm{installation}}\ [€] \Big/ \sum_{t=1}^{T} \frac{\text{Charging Energy}_t\ [\mathrm{kWh\ year^{-1}}]}{(1+i)^t}$$ Supplementary Equation (1b)

$$\mathrm{LCOC_{O\&M}}[€\ \mathrm{kWh^{-1}}] = \sum_{t=1}^{T} \frac{C_{\mathrm{O\&M}_t}\ [€\ \mathrm{year^{-1}}]}{(1+i)^t} \Bigg/ \sum_{t=1}^{T} \frac{\text{Charging Energy}_t\ [\mathrm{kWh\ year^{-1}}]}{(1+i)^t}$$ Supplementary Equation (1c)

$$\mathrm{LCOC_{infrastructure}}[€\ \mathrm{kWh^{-1}}] = \mathrm{LCOC_{equipment}}\ [€\ \mathrm{kWh^{-1}}] + \mathrm{LCOC_{installation}}\ [€\ \mathrm{kWh^{-1}}] + \mathrm{LCOC_{O\&M}}\ [€\ \mathrm{kWh^{-1}}]$$ Supplementary Equation (1d)

$$\mathrm{LCOC_{energy}}[€\ \mathrm{kWh^{-1}}] = \frac{C_{\mathrm{electricity_{energy}}}\ [€\ \mathrm{kWh^{-1}}]}{\eta\ [\%]}$$ Supplementary Equation (1e)

$$\mathrm{LCOC_{network}}[€\ \mathrm{kWh^{-1}}] = \frac{C_{\mathrm{electricity_{network}}}\ [€\ \mathrm{kWh^{-1}}]}{\eta\ [\%]}$$ Supplementary Equation (1f)

$$\mathrm{LCOC_{taxes\&levies}}[€\ \mathrm{kWh^{-1}}] = \frac{C_{\mathrm{electricity_{taxes\&levies}}}\ [€\ \mathrm{kWh^{-1}}]}{\eta\ [\%]}$$ Supplementary Equation (1g)

$$\mathrm{LCOC_{electricity}}[€\ \mathrm{kWh^{-1}}] = \mathrm{LCOC_{energy}}\ [€\ \mathrm{kWh^{-1}}] + \mathrm{LCOC_{network}}[€\ \mathrm{kWh^{-1}}] + \mathrm{LCOC_{taxes\&levies}}\ [€\ \mathrm{kWh^{-1}}]$$ Supplementary Equation (1h)

$$\mathrm{LCOC_{transaction}}[€\ \mathrm{kWh^{-1}}] = \left(\mathrm{LCOC_{infrastructure}}\ [€\ \mathrm{kWh^{-1}}] + \mathrm{LCOC_{electricity}}\ [€\ \mathrm{kWh^{-1}}]\right) \cdot C_{\mathrm{transaction}}\ [\%]$$ Supplementary Equation (1i)

$$\mathrm{LCOC_{total}}[€\ \mathrm{kWh^{-1}}] = \mathrm{LCOC_{infrastructure}}\ [€\ \mathrm{kWh^{-1}}] + \mathrm{LCOC_{electricity}}[€\ \mathrm{kWh^{-1}}] + \mathrm{LCOC_{transaction}}[€\ \mathrm{kWh^{-1}}]$$ Supplementary Equation (1j)

Supplementary Methods 4: Electricity cost of residential charging sites with solar rooftop PV

At the residential (PV) charging site, it is assumed that a solar rooftop PV system provides as much of the charging electricity as possible. Based on the analysis of realistic hour-by-hour load profiles, hourly shares of the grid electricity replaced during the day with electricity from a rooftop solar PV system are determined per country. The electricity cost at this charging is determined as a combination of costs of grid electricity and cost of the PV power on-site generated. The electricity cost of the on-site generated PV power is approximated by the levelized cost of electricity (LCOE) of solar rooftop PV in the different countries of the analysis. We use LCOE estimates based on high-resolution satellite images established in the study of Bódis et al.[1]. Here, we aggregate the study's results in each country and thus estimate the national average LCOE (listed in Supplementary Table 1).

Due to high prices of residential energy storage, it is assumed that no battery storage is available at the charging site. This means that the PV system can only supply electricity for charging if the PV output can immediately be used to charge a vehicle. However, the load curve of the PV output during the day only partially overlaps with the residential charging demand, which occurs mainly in the evening. Thus, only a certain share of the charging energy can be supplied by the PV system – the remaining charging energy must be drawn from the electricity grid. As a result, the electricity cost at the respective charging site is a weighted average of the LCOE for the PV share and the usual residential tariff used at the residential (grid) sites (see Supplementary Table 12).

To estimate the country-specific shares of the yearly charging energy that can be supplied by the PV system, a typical hourly load curve of residential charging is modeled and then overlaid with average hourly PV outputs in the different countries, corrected for the countries time zones and daylight saving time (see Supplementary Table 1 for details). Previous studies that evaluated real-world charging events at residential sites have shown that throughout the year, the charging load curves at residential sites mainly vary between workdays and weekend[2]. Many studies on charging demand therefore report normalized load curves for these two different daily patterns only[3–5]. Here, we use hourly charging profiles for weekdays and weekends reported in Noussan & Neirotti 2020[5] (see Supplementary Fig. 3-4) to produce a normalized annual charging profile which distributes the total annual residential charging energy in each country over the course of a year with an hourly resolution. The annual profile is comprised of 261 weekdays and 104 weekend days and normalized over the whole year. The load curve in kW is attained by multiplying the normalized hourly profile with the total annual charging energy. The annual charging profile is assumed to be representative of European charging behavior and is therefore applied uniformly across all countries.

For the PV output, we use a peer-reviewed and validated long-term dataset of European PV output patterns[6]. The study determines hourly PV capacity factors based on 30 years of validated hourly reanalysis and satellite data for all 30 countries considered. For this work, we calculate the long-term average capacity factor of each hour over the entire timespan to attain representative country-specific annual profiles. The actual PV output in each hour and each country is then attained by multiplying the rated power of a PV system with the calculated average country-specific capacity factor. In the model base case, we assume a system size of 10 kWp. See Supplementary Table 1 for the yearly PV output per country, as well as the effect of a smaller system size of only 5 kWp.

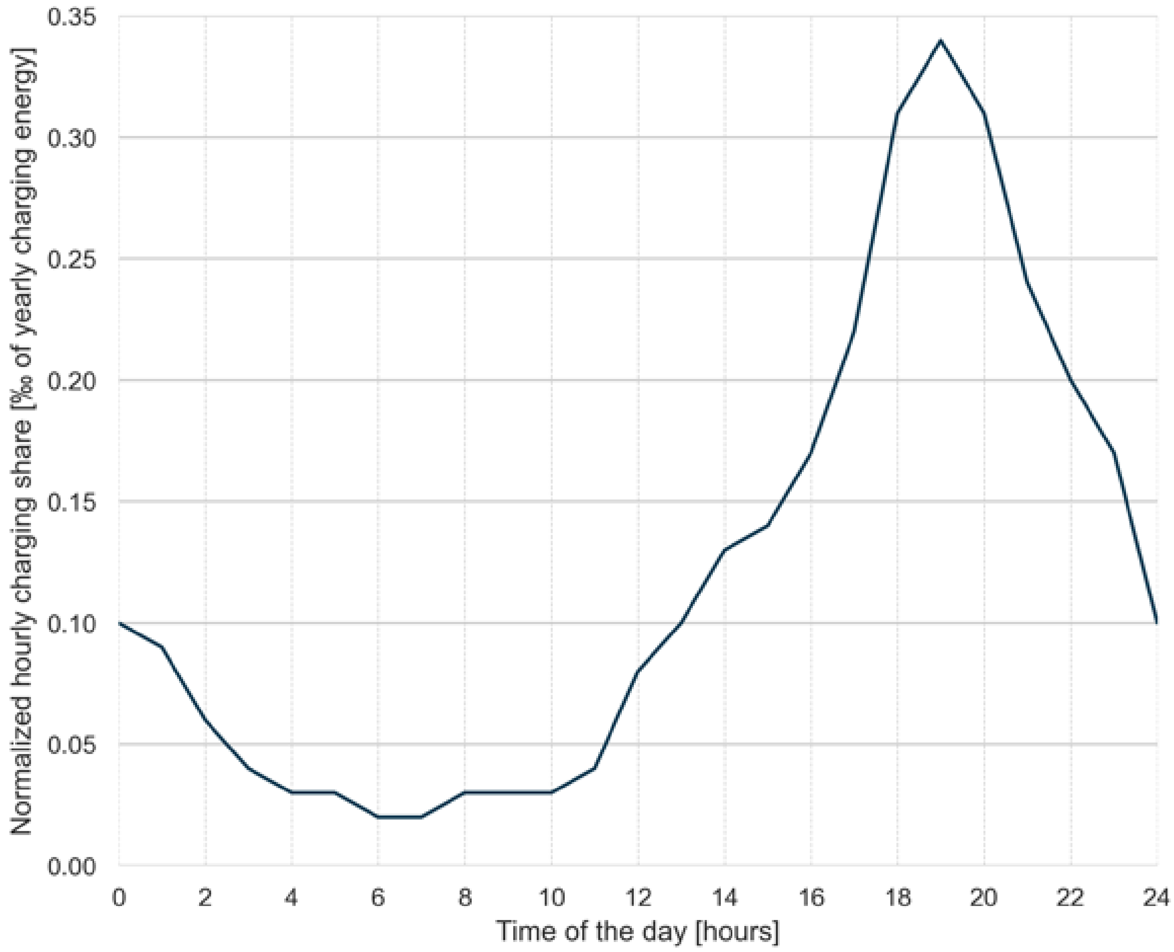

Supplementary Fig. 3. Solar PV charging share for weekdays. Normalized hourly residential charging profile on a weekday (adapted from Noussan & Neirotti[5]).

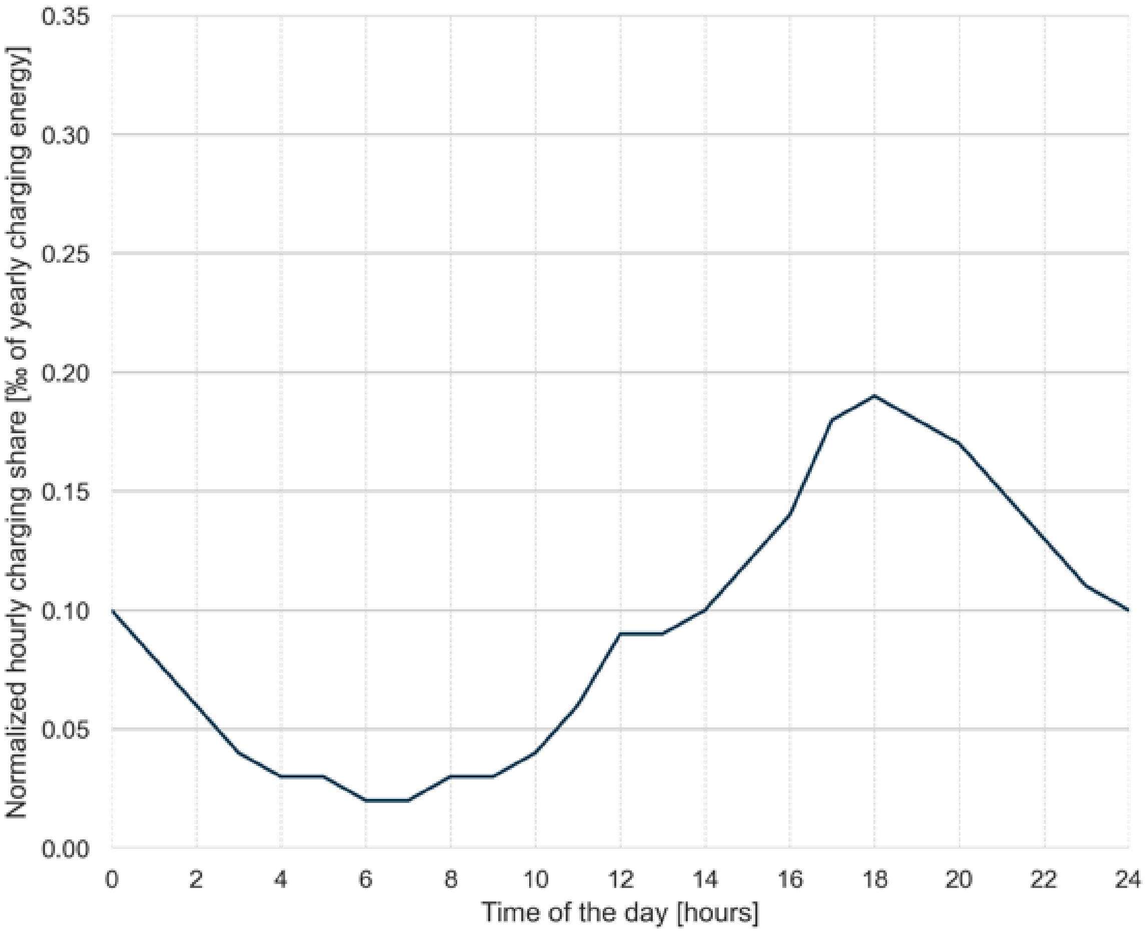

Supplementary Fig. 4. Solar PV charging share for weekend days. Normalized hourly residential charging profile on a weekend day (adapted from Noussan & Neirotti[5]).

Finally, for every country, the charging share that can be met by PV in each hour of the year is calculated by comparing the PV and charging load profiles described above. It is assumed that the residential charger is a prioritized consumer—whenever PV output is available, it is used for EV charging, limited maximally by the charging demand of the respective hour. Multiplying the hourly charging shares by the charging load curves yields the hourly charging energy that can be supplied by PV. By dividing the sum of this by the total yearly charging energy, we derive country-specific shares of charging energy supplied by PV over the entire year. These shares are listed in detail in Supplementary Table 1 for the selected PV system size of 10 kWp. As a sensitivity, we also report the effect of a smaller system size of only 5 kWp on the attainable PV share. In a last step, the resulting electricity cost at residential charging sites with PV can be calculated in each country by averaging the LCOE of PV and the residential grid tariff (see Supplementary Table 12) weighted with the determined PV share, see Supplementary Equation (2).

$$C_{\text{electricity}_{\text{country,residential (PV)}}}\ [€\ \text{kWh}^{-1}] = \text{PVShare}_{\text{country}}\ [\%] \cdot \text{LCOE}_{\text{PV}_{\text{country}}}\ [€\ \text{kWh}^{-1}] + \left(1 - \text{PVShare}_{\text{country}}\ [\%]\right) \cdot C_{\text{electricity}_{\text{country,residential (grid)}}}\ [€\ \text{kWh}^{-1}]$$

Supplementary Equation (2)

Supplementary Table 1. Cost and performance data of solar rooftop PV in European countries.

	Average LCOE of solar rooftop PV	Time zone	Maximum charging share with 5 kWp PV	Average daily output with 5 kWp PV	Maximum charging share with 10 kWp PV	Average daily output with 10 kWp PV
	**€ kWh^{-1}**	**to UTC**	**%**	**kWh**	**%**	**kWh**
**Austria**	0.1334	+1	37.8%	16.6	40.5%	33.3
**Belgium**	0.1350	+1	41.8%	14.7	44.5%	29.6
**Bulgaria**	0.1355	+2	42.3%	18.2	43.8%	36.5
**Croatia**	0.1574	+1	37.2%	17.2	39.4%	34.4
**Cyprus**	0.0750	+2	37.4%	21.9	39.0%	43.8
**Czech Republic**	0.1519	+1	38.7%	15.8	40.6%	31.7
**Denmark**	0.1350	+1	36.4%	13.4	39.8%	26.9
**Estonia**	0.1650	+2	37.3%	12.8	40.8%	25.7
**Finland**	0.1474	+2	36.8%	11.3	40.7%	22.7
**France**	0.1102	+1	43.9%	16.7	46.7%	33.5
**Germany**	0.1114	+1	38.7%	14.9	41.6%	29.9
**Greece**	0.1325	+2	42.5%	19.4	44.0%	38.9
**Hungary**	0.1650	+1	34.5%	16.6	36.9%	33.3
**Ireland**	0.1847	+0	39.7%	13.0	42.8%	26.0
**Italy**	0.1047	+1	40.6%	18.5	42.8%	37.2
**Latvia**	0.1950	+2	36.4%	13.0	40.0%	26.0
**Lithuania**	0.1671	+2	38.5%	13.7	41.7%	27.4
**Luxembourg**	0.1350	+1	42.4%	15.5	44.7%	31.1
**Malta**	0.0750	+1	39.0%	20.7	40.7%	41.5
**Netherlands**	0.1350	+1	40.2%	14.6	43.0%	29.3
**Poland**	0.1650	+1	36.5%	14.4	38.7%	28.9
**Portugal**	0.1025	+0	41.9%	19.9	44.0%	39.8
**Romania**	0.1525	+2	42.2%	16.9	44.1%	33.8
**Slovakia**	0.1392	+1	36.7%	16.1	38.8%	32.3
**Slovenia**	0.1650	+1	39.2%	16.9	40.9%	33.8
**Spain**	0.1112	+1	48.1%	20.1	50.8%	40.2
**Sweden**	0.1629	+1	36.7%	11.7	40.0%	23.4
**United Kingdom**	0.1485	+0	37.4%	12.8	40.4%	25.7
**Norway**	[a] 0.1474	+1	38.3%	11.7	41.8%	23.5
**Switzerland**	[b] 0.1334	+1	41.5%	18.5	43.8%	37.1
Sources	[1]	[7]	(see method above)	[6]	(see method above)	[6]

[a] assumed same LCOE as Finland (based on financing conditions and geographical latitude)
[b] assumed same LCOE as Austria (based on financing conditions, geographical latitude and in accordance with Swiss LCOE results[8])

Supplementary Methods 5: Input data of LCOC parameters and data processing

The following sections describe in detail the used data and how it the values are derived for each of the LCOC parameters. The full input dataset is attached in Supplementary Data 1.

Equipment cost

With the exception of the lowest power level (Low AC), the equipment price of each charging option is determined as the average price of a slice of the compiled equipment database (see Supplementary Data 2). Slice selections are performed by filtering the power level of the charging option and the standard which is appropriate for the charging site *(Home* for all residential sites, *Home/Commercial* for privately accessible sites and both *Home/Commercial* and *Commercial* for the publicly accessible sites). To better reflect the average equipment cost of the different charging options, additional criteria are considered in certain cases in order to remove outliers. First, for all residential site charging options, only chargers without RFID access control are selected. Second, for the two commercial charging options at medium AC power level (3.7 – 7.4 kW), charger models are scarce. We therefore assume these medium AC models to be 10 % cheaper than the corresponding high AC (11 – 22 kW) charging equipment, in line with findings from a recent US study on EV infrastructure costs[9]. Last, for both DC fast charging options, only the standard *Commercial* is relevant. The resulting equipment costs in € per plug, excluding value added tax (VAT), are summarized in Supplementary Table 2.

Supplementary Table 2. Input data of the cost of equipment for each charging option.

$C_{\text{equipment}_{\text{option}}}$	Low AC (< 2.3 kW)	Medium AC (3.7 – 7.4 kW)	High AC (11-22 kW)	DC (50 kW)
Residential (grid)	0	590	800	-
Residential (PV)	0	590	800	-
Commercial (priv. acc.)	0	1’490	1’660	23’330
Commercial (publ. acc.)	-	2’150	2’390	23’330
Units				€ per plug excl. VAT

Apart from the charger hardware, it is important to take into account potential additional costs for cables, which are required to connect the EV to any power source. For low AC charging at a socket, a Mode-2 cable is necessary (Type-2 connector to conventional household plug). Medium and high AC charging requires a Mode-3 cable (Type-2 to Type-2). Both cables cost between 200 – 300 € and thus represent significant additional equipment expenses. For DC charging, the European standard is the Type-2 Combined Charging System (CCS), which is even more expensive. However, here, the cost for cables are not added to the equipment cost for the following two reasons: First, home chargers typically come with an attached cable included in the purchase price of the EV and DC fast charging stations are often exclusively sold with permanently connected cables, similar to fuel hoses at petrol stations. For these charging options, the cable cost can therefore be safely neglected. Second, the purchase price of EVs often includes at least a basic cable to charge at a socket and more recent EV

models frequently come with a Mode-3 cable as well[10]. As a consequence, although EV owners indeed must bring their own cables to many commercial AC stations, it is assumed that the vast majority can use an existing cable that was included with the EV or a home charger they bought.

For the LCOC model, the equipment cost data is specified for each country. To this regard, we highlight two important points: First, within the EU internal market, price differences between Member States for the same equipment are minimal, a fact which has been confirmed by contacted industry experts. Second, the collected database of equipment costs revealed that price differences between similar products (i.e. equipment for the same charging power levels) are substantial. The price range of different chargers thus exceeds the between-country price differences, including the three non-EU countries and even when only comparing models for the same charging option. Therefore, it is assumed that the net equipment costs (excl. VAT) in Supplementary Table 2 are the same for all 30 countries of the analysis. At the two residential charging sites, equipment costs are calculated using country-specific VAT rates, as shown in Supplementary Equation (3a). For the two commercial charging sites, it is assumed that commercial operators may reclaim the VAT so it is therefore ignored, as seen in Supplementary Equation (3b).

$$C_{\text{equipment}_{\text{country,residential option}}}\ [€] = C_{\text{equipment}_{\text{residential option}}}\ [€] \cdot \left(1 + \text{VAT}_{\text{country}}\ [\%]\right) \quad \text{Supplementary Equation (3a)}$$

$$C_{\text{equipment}_{\text{country,commercial option}}}\ [€] = C_{\text{equipment}_{\text{commercial option}}}\ [€] \quad \text{Supplementary Equation (3b)}$$

Installation cost

To determine installation costs, we collect diverse cost information available in the literature (see Supplementary Table 3). We then estimate installation costs and their labor shares for the different charging options and validate the cost compilation with industry experts. The resulting input data for installation cost in € per plug (excluding value added tax (VAT) and grid connection) as well as the respective labor shares, are summarized in Supplementary Table 4.

Supplementary Table 3. Literature and industry values for installation costs.

	Medium AC (3.7 – 7.4 kW)	High AC (11 – 22 kW)	DC (50 kW)
Residential (grid) & Residential (PV)	1286 $ (US, 2020) [11]<br>1354 $ (US, 2018) [12]<br>200-800 $ (US, 2014) [13]<br>1325-1427 $ (US, 2013) [14]	1286 $ (US, 2020) [11]<br>1354 $ (US, 2018) [12]<br>700-1800 $ (US, 2014) [13]<br>1325-1427 $ (US, 2013) [14]<br>540-1548 € (DE, 2021) [15]<br>1624-2573 CHF (CH, 2021) [16]	-
Commercial (priv. acc.) & Commercial (publ. acc.)	2500 $ (range: 510-6620 $) (US, 2020) [11]<br>3108 $ (US, 2018) [12]<br>2050-5000 $ (garage) / 3800-10150 $ (curbside) (US, 2014) [13]<br>3367-4173 $ (US, 2013) [14]	2500 $ (range: 510-6620 $) (US, 2020) [11]<br>3108 $ (US, 2018) [12]<br>2550-6000 $ (garage) / 4300-13150 $ (curbside) (US, 2014) [13]<br>3367-4173 $ (US, 2013) [14]<br>2940-5670 CHF (CH, 2021) [16]	20000 $ (range: 10500 -46500 $) (US, 2020) [11]<br>22626 $ (US, 2018) [12]<br>17650-45400 $ (US, 2014) [13]<br>36’992 $ (US, 2019) [17]<br>20’000-25’000 $ (US, 2018) [18]

Supplementary Table 4. Input data of the cost of installation for each charging option. Installation costs include transformer costs for DC charging. The numbers in parentheses represent the estimated labor share of the respective installation.

$C_{\text{installation}_{\text{option}}}$	Low AC (< 2.3 kW)	Medium AC (3.7 – 7.4 kW)	High AC (11-22 kW)	DC (50 kW)
Residential (grid)	0	1’100 (50 %)	1’200 (50 %)	-
Residential (PV)	0	1’100 (50 %)	1’200 (50 %)	-
Commercial (priv. acc.)	0	2’200 (75 %)	2’700 (75 %)	20’000 (65 %)
Commercial (publ. acc.)	-	2’200 (80 %)	2’700 (80 %)	20’000 (65 %)
Units	€ per plug, excl. VAT and grid connection (% labor)			

The labor share for each project type (see Supplementary Table 4) is used to account for country differences of material and labor costs. Labor cost, as a share of the total installation cost, is scaled based on national hourly labor rates in the construction sector (see Supplementary Table 10 for details). We therefore define a country-specific labor factor, see Supplementary Equation (4a). This factor is then used to scale the labor share in both residential and commercial options, see Supplementary Equations (4b) and (4c). To address material cost differences, the country-specific VAT is added for all residential charging options and ignored at the commercial sites, same as with equipment costs.

$$\text{LaborFactor}_{\text{country}}\,[-] = \text{LaborCost}_{\text{country}}\,[€\ \text{h}^{-1}]\ /\ \text{LaborCost}_{\text{EU28}}\,[€\ \text{h}^{-1}]$$ Supplementary Equation (4a)

$$C_{\text{installation}_{\text{country,residential option}}}\,[€] = \Big(C_{\text{installation}_{\text{residential option}}}[€]\cdot(1-\text{LaborShare}_{\text{residential option}}\,[\%]) + C_{\text{installation}_{\text{residential option}}}\,[€]\cdot\text{LaborShare}_{\text{residential option}}\,[\%]\cdot\text{LaborFactor}_{\text{country}}\,[-]\Big)\cdot\big(1+\text{VAT}_{\text{country}}\,[\%]\big)$$ Supplementary Equation (4b)

$$C_{\text{installation}_{\text{country,commercial option}}}\,[€] = C_{\text{installation}_{\text{commercial option}}}\,[€]\cdot(1-\text{LaborShare}_{\text{commercial option}}\,[\%]) + C_{\text{installation}_{\text{commercial option}}}\,[€]\cdot\text{LaborShare}_{\text{commercial option}}\,[\%]\cdot\text{LaborFactor}_{\text{country}}\,[-]$$ Supplementary Equation (4c)

O&M

The third infrastructure component besides equipment and installation is the annual cost for operation and maintenance (O&M) over the lifetime of a project. In general, EV charging equipment is assumed to have very low maintenance requirements. Consequently, for technical maintenance of residential and commercial privately accessible charging sites, we assume annual costs of 2 % of the equipment cost. This covers 1-2 maintenance or repair incidents per year, based on expert interviews. At publicly accessible stations, O&M costs are assumed to be 4 % of equipment cost due to higher station strain. Besides the technical maintenance (O&M), for the operation of commercial, publicly accessible charging options we assume an additional 180 € per year (or 15 € per months) of service costs for billing, network and load management systems, based on expert interviews. The resulting yearly O&M costs for each charging option can be viewed in Supplementary Table 5.

Supplementary Table 5. Input data of the yearly O&M cost for each charging option. The numbers in parentheses represent the assumed annual percentage of the equipment cost needed for technical maintenance. For the operation of commercial, publicly accessible charging options an additional service cost for billing, network and load management systems of 180 € per year is added.

$C_{\text{O\&M}_{t,\text{option}}}$	Low AC (< 2.3 kW)	Medium AC (3.7 – 7.4 kW)	High AC (11-22 kW)	DC (50 kW)
Residential (grid)	0	12 (2%)	16 (2 %)	-
Residential (PV)	0	12 (2 %)	16 (2 %)	-
Commercial (priv. acc.)	0	30 (2 %)	33 (2 %)	467 (2 %)
Commercial (publ. acc.)	-	266 (4 % + 180)	276 (4 % + 180)	1113 (4 % + 180)
Units	€ per plug per year, excl. VAT (% of equipment cost)			

Charging energy

As described in the Methods, we apply a user-centered approach based on average annual driving distances in order to estimate annual charging energies of residential charging options, see Supplementary Equation (5). The employed annual driving distances are reported in Supplementary Table 10. To estimate average annual charging energies of commercial options, we take the average charging energy of single charging events and average number of events per day at different charging options from the literature (see Methods) and then extrapolate this to annual charging amounts. Real-world charging data indicates that stations with higher power levels generally serve more customers per day, due to shorter charging times. In contrast, the charging energy per event is predominantly dependent on the typical use patterns at the charging stations: While increased AC power levels typically result in higher charging amounts, DC stations are again mainly used for smaller battery fill-ups of around 10 kWh, taking around 10-15 minutes at a 50 kW station. Furthermore, it is assumed that commercial stations are used on working days only (260 days per year). This approach gives the daily charging patterns and resulting annual charging energies for commercial charging options summarized in Supplementary Table 6. The data was validated with industry experts.

Supplementary Table 6. Input data of the annual charging energy for each commercial charging option.

**Charging Energy**	**Low AC (< 2.3 kW)**	**Medium AC (3.7 – 7.4 kW)**	**High AC (11-22 kW)**	**DC (50 kW)**
Residential (grid)		(user-centered approach)		-
Residential (PV)		(user-centered approach)		-
Commercial (priv. acc.)	3’400 (1 x 13kWh)	3’400 (1 x 13kWh)	5’000 (1.5 x 19kWh)	16’000 (6 x 10kWh)
Commercial (publ. acc.)	-	3’400 (1 x 13kWh)	5’000 (1.5 x 19kWh)	16’000 (6 x 10kWh)
Units	kWh per year and plug (daily charging pattern: events d^{-1} x kWh $event^{-1}$)			

$$\text{ChargingEnergy}_{t,\text{country,residential option}}\ [\text{kWh year}^{-1}] = \text{ChargingShare}_{\text{residential option}}\ [\%] \cdot \frac{\text{Distance}_{t,\text{country}}\ [\text{km year}^{-1}]}{100\ [\text{km}]} \cdot \text{FuelConsumption}[\text{kWh } 100\text{km}^{-1}]$$ Supplementary Equation (5)

Interest rate

To discount annual O&M cost and charging energy, we assume an interest rate of 3 % for residential charging sites, based on current bank interest rates for household loans and mortgages (1-5 % in the Euro Area[19]) and common estimates of the social discount rate (2-3 %[20]). For the commercial charging sites the cost of capital is not always consistent in literature. For Europe, we estimate a discount rate of 7 % (representing long-term averages of the cost of capital of typical companies) based on a number of studies[21–23] (see Supplementary Table 7).

Supplementary Table 7. Input data of the interest rates for each charging option.

i_{option}	**Low AC (< 2.3 kW)**	**Medium AC (3.7 – 7.4 kW)**	**High AC (11-22 kW)**	**DC (50 kW)**
Residential (grid)	3	3	3	-
Residential (PV)	3	3	3	-
Commercial (priv. acc.)	7	7	7	7
Commercial (publ. acc.)	-	7	7	7
Units				% interest rate

Interest rates are applied uniformly across all 30 countries in the analysis. For (renewable) energy system models, however, it has been shown that disregarding country differences in cost of capital can significantly bias model results[24]. Despite this, this study exhibits several differences to the case above: First, charging infrastructure is less capital-intensive than renewable energy (RE) generation technologies, where the majority of the cost is upfront. Thus, the cost of capital tends to be less determinate. Second, while for RE technologies the spread of cost of capital between different technologies is small compared to the country spreads[24], this is not so for vehicle charging infrastructure. Cost of capital variation between different charging options is considerable (see Supplementary Table 7) and within-country differences will thus (at least) be comparable to between-country differences estimated in previous studies[25]. Last, costs of capital differ particularly across countries of different in-come and investment risk classes—as we analyze 30 European countries, the variations are much lower than on a global scale. In sum, there are several reasons why we justify geographic uniform cost of capital. If the model were expanded to even higher power levels (e.g. 150 or 350 kW) and thus to infrastructure that is more capital-intensive, a re-evaluation of country-specific interest rates may be considered.

Transaction cost

At publicly accessible stations, charging service is typically for a fee and entails additional expenses for the financial transaction. We therefore assume an additional 2 % surcharge on the total LCOC to account for financial transaction fees incurred. For all other charging sites, the transaction cost is assumed to be 0 % (see Supplementary Table 8).

Supplementary Table 8. Input data of the cost of transaction for each charging option.

$C_{\text{transaction}_{\text{option}}}$	**Low AC (< 2.3 kW)**	**Medium AC (3.7 – 7.4 kW)**	**High AC (11-22 kW)**	**DC (50 kW)**
Residential (grid)	0	0	0	-
Residential (PV)	0	0	0	-
Commercial (priv. acc.)	0	0	0	0
Commercial (publ. acc.)	-	2	2	2
Units				% on total due payment

Efficiency

In order to be consistent with the infrastructure cost and to set the system boundary of our model directly at the plug, we consider charging efficiencies of different types of chargers. The charging efficiency is primarily dependent on the equipment type. We assume 100 % efficiency for socket charging without charging equipment (low AC) and 99.5 % in models for medium and high AC charging, based on data from equipment testing[26]. For DC fast charging, we consider both the transformer and the charger itself. We assume 98 % efficiency of the transformer[27] and 94 % for a typical 50 kW DC fast charging station[28], resulting in an overall efficiency of 92.1 % (98 % x 94 %) (see Supplementary Table 9).

Supplementary Table 9. Input data of the charging efficiency for each charging option.

η_{option}	**Low AC (< 2.3 kW)**	**Medium AC (3.7 – 7.4 kW)**	**High AC (11-22 kW)**	**DC (50 kW)**
Residential (grid)	100	99.5	99.5	-
Residential (PV)	100	99.5	99.5	-
Commercial (priv. acc.)	100	99.5	99.5	7
Commercial (publ. acc.)	-	99.5	99.5	92.1
Units				% efficiency

Supplementary Methods 6: Auxiliary country-specific input data

Supplementary Table 10. Country-specific auxiliary data used in the model.

	Country code (ISO Alpha-2)	Population on 1 January 2019	Value added tax (standard rate)	Labor cost in construction (2019)	Average distance driven
		million	%	€ h^{-1}	km $year^{-1}$
**Austria**	AT	8.859	20.0	35.70	13800
**Belgium**	BE	11.456	21.0	36.40	14800
**Bulgaria**	BG	7.000	20.0	4.70	8000
**Croatia**	HR	4.076	25.0	9.90	12500
**Cyprus**	CY	0.876	19.0	10.93	10000
**Czech Republic**	CZ	10.650	21.0	12.10	8000
**Denmark**	DK	5.806	25.0	41.60	15900
**Estonia**	EE	1.325	20.0	14.60	14700
**Finland**	FI	5.518	24.0	35.00	13600
**France**	FR	67.178	20.0	33.40	13100
**Germany**	DE	83.019	19.0	29.60	13800
**Greece**	GR	10.725	24.0	10.30	10000
**Hungary**	HU	9.773	27.0	8.30	17000
**Ireland**	IE	4.904	23.0	27.80	12600
**Italy**	IT	59.817	22.0	24.00	9500
**Latvia**	LV	1.920	21.0	10.40	14700
**Lithuania**	LT	2.794	21.0	8.40	14700
**Luxembourg**	LU	0.614	17.0	28.00	13000
**Malta**	MT	0.494	18.0	11.20	10000
**Netherlands**	NL	17.282	21.0	37.10	13000
**Poland**	PL	37.973	23.0	10.10	8000
**Portugal**	PT	10.277	23.0	10.20	13200
**Romania**	RO	19.414	19.0	6.10	8000
**Slovakia**	SK	5.450	20.0	10.30	8000
**Slovenia**	SI	2.081	22.0	15.30	8000
**Spain**	ES	46.937	21.0	20.20	13200
**Sweden**	SE	10.230	25.0	36.50	11700
**United Kingdom**	GB	66.647	20.0	27.80	12600
**Norway**	NO	5.328	25.0	42.20	12400
**Switzerland**	CH	8.545	7.7	48.12	11000
**EU28**		513.094		25.60	
Sources/ Comments	[29] Note: The EU uses other conventions for GR and GB: GR → EL GB → UK	[30]	NO[31], CH[32], other countries[33]	CH[34,35], other countries[36] Note: CY is estimated from GR, scaled by the relation of labor cost in all industry and services ["B to S excl. O"] between the two countries	compiled from several sources[37–40]

Supplementary Table 11. Country-specific auxiliary on share of population living in owner-occupied dwellings according to Eurostat (values are not used directly in the model but rather for depth of discussion)[41].

	Country code (ISO Alpha-2)	Owner	Tenant
		%	%
**Austria**	AT	55.0	45.0
**Belgium**	BE	70.9	29.1
**Bulgaria**	BG	82.3	17.7
**Croatia**	HR	90.0	10.0
**Cyprus**	CY	72.5	27.5
**Czech Republic**	CZ	78.2	21.8
**Denmark**	DK	61.7	38.3
**Estonia**	EE	81.4	18.6
**Finland**	FI	71.6	28.4
**France**	FR	64.9	35.1
**Germany**	DE	51.7	48.3
**Greece**	GR	73.9	26.1
**Hungary**	HU	86.3	13.7
**Ireland**	IE	69.5	30.5
**Italy**	IT	72.3	27.7
**Latvia**	LV	80.9	19.1
**Lithuania**	LT	90.3	9.7
**Luxembourg**	LU	73.9	26.1
**Malta**	MT	81.4	18.6
**Netherlands**	NL	69.0	31.0
**Poland**	PL	83.4	16.6
**Portugal**	PT	75.2	24.8
**Romania**	RO	96.0	4.0
**Slovakia**	SK	89.5	10.5
**Slovenia**	SI	75.1	24.9
**Spain**	ES	77.8	22.2
**Sweden**	SE	65.2	34.8
**United Kingdom**	GB	82.9	17.1
**Norway**	NO	63.4	36.6
**Switzerland**	CH	42.5	57.5
**EU28**		69.2	30.8

Supplementary Methods 7: Auxiliary input data of European grid electricity prices

Supplementary Table 12. Average household electricity prices 2019 in European countries according to Eurostat (Consumption from 5 000 kWh to 14 999 kWh - band DD)[42].

	Energy	Network	Value added tax	Renewable taxes	Capacity taxes	Environ. taxes	Nuclear taxes	Other taxes	Total
	€ kWh^{-1}	€ kWh^{-1}	€ kWh^{-1}	€ kWh^{-1}	€ kWh^{-1}	€ kWh^{-1}	€ kWh^{-1}	€ kWh^{-1}	€ kWh^{-1}
**Austria**	0.0637	0.0555	0.0301	0.0154	0.0000	0.0150	0.0000	0.0010	0.1807
**Belgium**	0.0780	0.0976	0.0447	0.0353	0.0013	0.0019	0.0007	0.0015	0.2610
**Bulgaria**	0.0546	0.0258	0.0161	0.0000	0.0000	0.0000	0.0000	0.0000	0.0965
**Croatia**	0.0569	0.0415	0.0146	0.0142	0.0000	0.0000	0.0000	0.0000	0.1272
**Cyprus**	0.1201	0.0312	0.0329	0.0096	0.0065	0.0150	0.0000	0.0008	0.2161
**Czech Republic**	0.0670	0.0265	0.0238	0.0195	0.0000	0.0012	0.0000	0.0000	0.1380
**Denmark**	0.0539	0.0445	0.0472	0.0075	0.0000	0.0829	0.0000	0.0000	0.2360
**Estonia**	0.0521	0.0421	0.0218	0.0104	0.0000	0.0045	0.0000	0.0000	0.1309
**Finland**	0.0488	0.0502	0.0292	0.0000	0.0001	0.0224	0.0000	0.0000	0.1507
**France**	0.0625	0.0441	0.0258	0.0000	0.0030	0.0318	0.0000	0.0000	0.1672
**Germany**	0.0547	0.0592	0.0421	0.0669	0.0073	0.0205	0.0000	0.0137	0.2644
**Greece**	0.0897	0.0234	0.0137	0.0170	0.0000	0.0022	0.0000	0.0238	0.1698
**Hungary**	0.0419	0.0424	0.0228	0.0000	0.0000	0.0000	0.0000	0.0000	0.1071
**Ireland**	0.1108	0.0678	0.0252	0.0040	0.0014	0.0000	0.0000	0.0029	0.2121
**Italy**	0.0894	0.0378	0.0211	0.0545	0.0000	0.0218	0.0028	0.0031	0.2305
**Latvia**	0.0570	0.0517	0.0269	0.0192	0.0000	0.0000	0.0000	0.0002	0.1550
**Lithuania**	0.0474	0.0431	0.0208	0.0090	0.0000	0.0000	0.0000	0.0000	0.1203
**Luxembourg**	0.0539	0.0559	0.0115	0.0330	0.0000	0.0010	0.0000	0.0000	0.1553
**Malta**	0.1172	0.0250	0.0072	0.0000	0.0000	0.0015	0.0000	0.0000	0.1509
**Netherlands**	0.0701	0.0260	0.0366	0.0189	0.0000	0.0593	0.0000	0.0000	0.2109
**Poland**	0.0416	0.0400	0.0237	0.0056	0.0033	0.0094	0.0000	0.0032	0.1268
**Portugal**	0.0676	0.0454	0.0383	0.0398	0.0022	0.0010	0.0000	0.0111	0.2054
**Romania**	0.0596	0.0413	0.0223	0.0153	0.0000	0.0010	0.0000	0.0000	0.1395
**Slovakia**	0.0510	0.0282	0.0229	0.0206	0.0114	0.0000	0.0033	0.0000	0.1374
**Slovenia**	0.0573	0.0462	0.0258	0.0104	0.0001	0.0030	0.0000	0.0000	0.1428
**Spain**	0.0701	0.0405	0.0346	0.0275	0.0035	0.0080	0.0000	0.0151	0.1993
**Sweden**	0.0496	0.0544	0.0346	0.0034	0.0000	0.0311	0.0000	0.0000	0.1731
**United Kingdom**	0.0896	0.0439	0.0093	0.0213	0.0032	0.0000	0.0000	0.0282	0.1955
**Norway**	0.0503	0.0405	0.0237	0.0000	0.0000	0.0165	0.0000	0.0000	0.1310
**Switzerland**[a]	0.0641	0.0701	0.0128	0.0207	0.0000	0.0000	0.0000	0.0107	0.1784
**EU28**	0.0670	0.0462	0.0278	0.0245	0.0028	0.0179	0.0003	0.0084	0.1949

[a] Swiss electricity prices are added manually, using a methodology equivalent to Eurostat and coordinated with the Swiss Federal Statistical Office (see Supplementary Methods 8 for details)

Supplementary Table 13. Average non-household electricity prices 2019 in European countries according to Eurostat (Consumption 20 000 kWh to 499 000 kWh - band IB)[43].

	Energy	Network	Value added tax	Renewable taxes	Capacity taxes	Environ. taxes	Nuclear taxes	Other taxes	Total
	€ kWh^{-1}	€ kWh^{-1}	€ kWh^{-1}	€ kWh^{-1}	€ kWh^{-1}	€ kWh^{-1}	€ kWh^{-1}	€ kWh^{-1}	€ kWh^{-1}
**Austria**	0.0530	0.0459	0.0252	0.0114	0.0000	0.0150	0.0000	0.0013	0.1518
**Belgium**	0.0543	0.0623	0.0321	0.0345	0.0018	0.0017	0.0006	0.0012	0.1885
**Bulgaria**	0.0719	0.0241	0.0194	0.0000	0.0000	0.0010	0.0000	0.0000	0.1164
**Croatia**	0.0655	0.0445	0.0162	0.0142	0.0000	0.0006	0.0000	0.0000	0.1410
**Cyprus**	0.1201	0.0293	0.0324	0.0100	0.0064	0.0154	0.0000	0.0007	0.2143
**Czech Republic**	0.0648	0.0575	0.0293	0.0161	0.0000	0.0011	0.0000	0.0001	0.1689
**Denmark**	0.0443	0.0400	0.0523	0.0065	0.0000	0.1184	0.0000	0.0000	0.2615
**Estonia**	0.0471	0.0371	0.0198	0.0104	0.0000	0.0045	0.0000	0.0000	0.1189
**Finland**	0.0444	0.0367	0.0212	0.0000	0.0001	0.0069	0.0000	0.0000	0.1093
**France**	0.0573	0.0421	0.0252	0.0000	0.0029	0.0268	0.0000	0.0000	0.1543
**Germany**	0.0347	0.0524	0.0356	0.0669	0.0072	0.0205	0.0000	0.0059	0.2232
**Greece**	0.0799	0.0246	0.0125	0.0156	0.0000	0.0050	0.0000	0.0180	0.1556
**Hungary**	0.0610	0.0400	0.0279	0.0066	0.0000	0.0009	0.0000	0.0027	0.1391
**Ireland**	0.0920	0.0631	0.0214	0.0040	0.0015	0.0003	0.0000	0.0012	0.1835
**Italy**	0.0810	0.0280	0.0347	0.0600	0.0000	0.0123	0.0021	0.0043	0.2224
**Latvia**	0.0492	0.0561	0.0273	0.0237	0.0000	0.0000	0.0000	0.0011	0.1574
**Lithuania**	0.0515	0.0438	0.0220	0.0090	0.0000	0.0004	0.0000	0.0000	0.1267
**Luxembourg**	0.0549	0.0416	0.0089	0.0140	0.0000	0.0006	0.0000	0.0000	0.1200
**Malta**	0.1254	0.0250	0.0076	0.0000	0.0000	0.0015	0.0000	0.0000	0.1595
**Netherlands**	0.0539	0.0258	0.0255	0.0135	0.0000	0.0287	0.0000	0.0000	0.1474
**Poland**	0.0497	0.0444	0.0265	0.0050	0.0041	0.0100	0.0000	0.0019	0.1416
**Portugal**	0.0683	0.0368	0.0313	0.0087	0.0126	0.0010	0.0000	0.0168	0.1755
**Romania**	0.0606	0.0337	0.0209	0.0150	0.0000	0.0005	0.0000	0.0000	0.1307
**Slovakia**	0.0579	0.0547	0.0298	0.0206	0.0114	0.0013	0.0033	0.0000	0.1790
**Slovenia**	0.0587	0.0371	0.0250	0.0144	0.0001	0.0030	0.0000	0.0000	0.1383
**Spain**	0.0802	0.0265	0.0302	0.0180	0.0033	0.0070	0.0000	0.0088	0.1740
**Sweden**	0.0441	0.0381	0.0215	0.0034	0.0000	0.0005	0.0000	0.0000	0.1076
**United Kingdom**	0.0801	0.0416	0.0321	0.0209	0.0014	0.0082	0.0000	0.0168	0.2011
**Norway**	0.0434	0.0268	0.0200	0.0000	0.0000	0.0100	0.0000	0.0000	0.1002
**Switzerland**[a]	0.0646	0.0709	0.0128	0.0207	0.0000	0.0000	0.0000	0.0098	0.1788
**EU28**	0.0594	0.0408	0.0295	0.0272	0.0028	0.0143	0.0003	0.0048	0.1791

[a] Swiss electricity prices are added manually, using a methodology equivalent to Eurostat and coordinated with the Swiss Federal Statistical Office (see Supplementary Methods 8 for details)

Supplementary Methods 8: Estimation of average Swiss electricity prices

Switzerland is not part of the European statistics of electricity prices in Eurostat. In the following, the methodology to add Swiss electricity prices to the dataset is described, which is based on the official Eurostat[44,45] methodology.

According to the official Eurostat methodology "prices are to be weighted according to the market share of [..] electricity supply undertakings in each consumption band. […] the data must cover a representative share of the national market. Market shares should be based on the quantity of electricity […] billed by the suppliers to household end-users. If possible, market shares will be calculated separately for each band"[45]. In Switzerland, the population in the service area of each utility is used as a proxy for the quantity of electricity and thus to estimate the market share. For this, we merge official data on Swiss population by municipality[46] with the list of Swiss municipalities and their responsible electricity grid operators (as of 24.11.2020) by the Swiss Federal Electricity Commission ElCom[47]. Next, the complete list of electricity tariffs in 2019 in each consumption category from all electricity grid operators[47] is merged to this dataset. The population in the service area of each operator is then used to calculate weighted national average electricity prices separately for each consumption category. Finally, the prices of the Swiss consumption categories that best match the used Eurostat consumption bands are added to the European dataset as input to the LCOC model: For household prices, the Swiss category H5 (consumption 7'500 kWh) best matches the European consumption band DD, for non-household prices, the Swiss category C3 (consumption 150'000 kWh, power 50 kW) best matches the European consumption band IB.

Supplementary Methods 9: Conversion rates

Supplementary Table 14. Average annual currency exchange rates to € in 2019.

	**Swiss Francs**	**British Pounds**
Average exchange rates 2019	1.11247283 CHF per € [35]	0.87681 £ per € [48]

Supplementary Methods 10: Additional data on user profiles

Supplementary Table 15. Assumed charging shares of charging options in the five different user profiles defined in the study (based on a number of sources [11,49–57]).

	Average user	Wallbox user	Wallbox user with PV	Commercial user	Socket user
Residential (grid) – low AC	25%				80%
Residential (grid) – medium AC	25%	37.5%			
Residential (grid) – high AC	25%	37.5%			
Residential (PV) – low AC					
Residential (PV) – medium AC			37.5%		
Residential (PV) – high AC			37.5%		
Commercial (priv. acc.) – low AC	3%				20%
Commercial (priv. acc.) – medium AC	6%	7%	7%	35%	
Commercial (priv. acc.) – high AC	6%	8%	8%	25%	
Commercial (priv. acc.) – DC				5%	
Commercial (pub. acc.) – medium AC	2%	2%	2%	8%	
Commercial (pub. acc.) – high AC	3%	3%	3%	12%	
Commercial (pub. acc.) – DC	5%	5%	5%	15%	
Total	100%	100%	100%	100%	100%

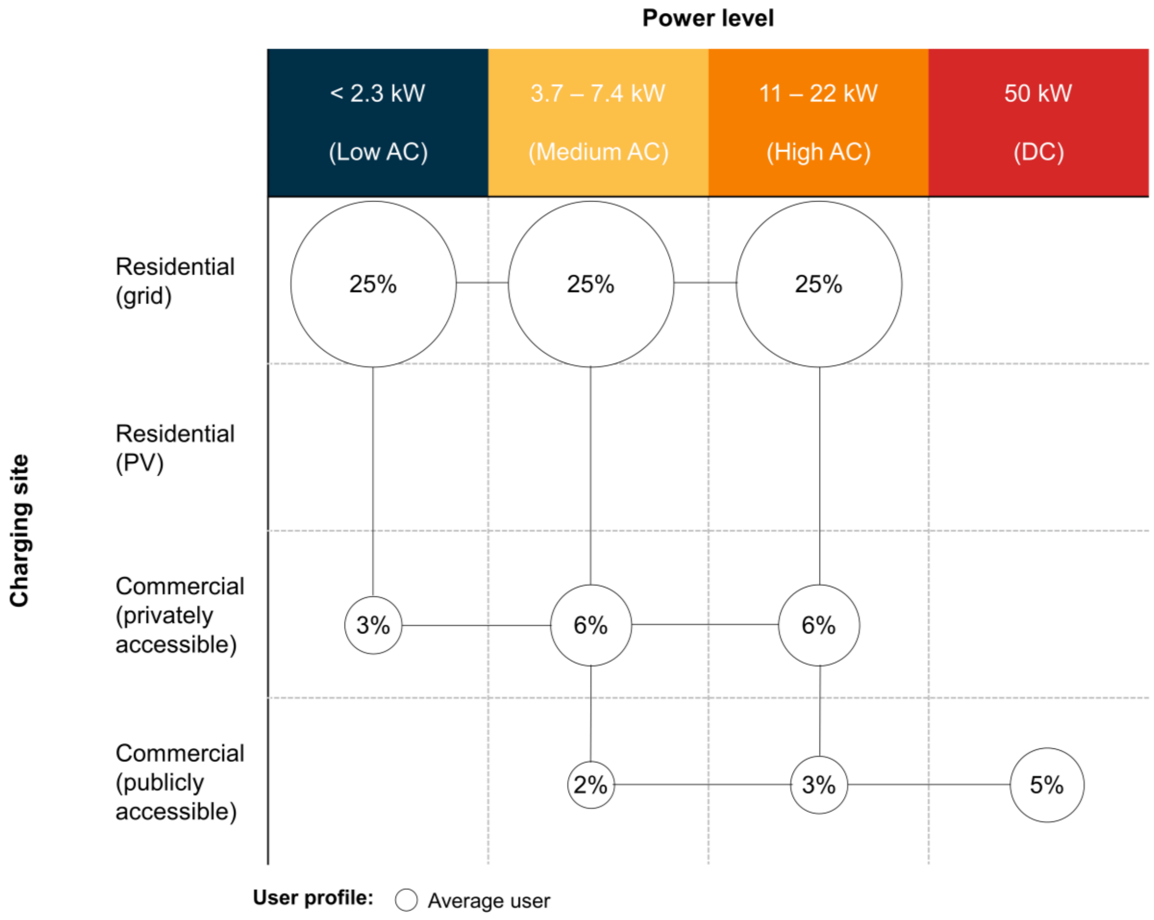

Supplementary Fig. 5. Matrix of charging options with charging shares of the defined *Average user* profile. Percentage shares of each charging option for the *Average user* profile are proportionally sized with bubbles. Shares within the user profile sum up to 100 % and are linked.

Supplementary Methods 11: Interviewees

Supplementary Table 16. List of interviewees. Correspondence was held under the Chatham House Rule[1] and is therefore anonymized.

	Organization	Expertise in EV Charging	Interviewee's Role
1	Equipment manufacturer	- Technology (technical specifications / cost)<br>- Infrastructure projects (cost)	Senior project manager
2	Equipment manufacturer	- Technology (technical specifications / cost)<br>- Infrastructure projects (cost)	Head of market field
3	Consulting	- Infrastructure projects (cost)<br>- Infrastructure operation & utilization<br>- Cost modeling	Head of market field
4	Consulting	- Infrastructure projects (cost)<br>- Infrastructure operation & utilization<br>- Cost modeling	Division head
5	Equipment redistributor	- Technology (technical specifications / cost)<br>- Infrastructure projects (cost)	CEO
6	Equipment manufacturer	- Technology (technical specifications / cost)<br>- Infrastructure projects (cost)	Division head
7	Equipment manufacturer	- Technology (technical specifications / cost)<br>- Infrastructure projects (cost)	Division head
8	Consulting	- Infrastructure projects (cost)<br>- Infrastructure operation & utilization	Head of market field
9	Infrastructure project developer	- Technology (technical specifications / cost)<br>- Infrastructure projects (cost)<br>- Infrastructure operation & utilization	Division head
10	Research	- Technology (technical specifications / cost)<br>- Infrastructure projects (cost)<br>- Infrastructure operation & utilization	Researcher
11	Research	- Technology (technical specifications / cost)<br>- Infrastructure projects (cost)<br>- Infrastructure operation & utilization	Group leader
12	Research	- Technology (technical specifications / cost)<br>- Infrastructure projects (cost)<br>- Infrastructure operation & utilization	Group leader

[1] "When a meeting, or part thereof, is held under the Chatham House Rule, participants are free to use the information received, but neither the identity nor the affiliation of the speaker(s), nor that of any other participant, may be revealed." (see: https://www.chathamhouse.org/about/chatham-house-rule)

Supplementary Discussion 1: General sensitivity analysis

All results presented in this study are deterministic and capture the LCOC of the specific parameter set that was chosen as the model base case (see Supplementary Methods 5). To account for uncertainty of input data and to understand the most influential model factors, we conduct a sensitivity analysis of the LCOC model. The results of the sensitivity analysis informed the selection of certain modeling parameters that were investigated in more depth (e.g. for Figure 5 and Figure 6 in the main text).

Supplementary Fig. 6-18 show the impact of a 20 % increase and decrease of each LCOC input parameter (everything else held constant) on the European average LCOC at the different charging options. The yearly charging energy and electricity costs emerge as key determinants of the LCOC.

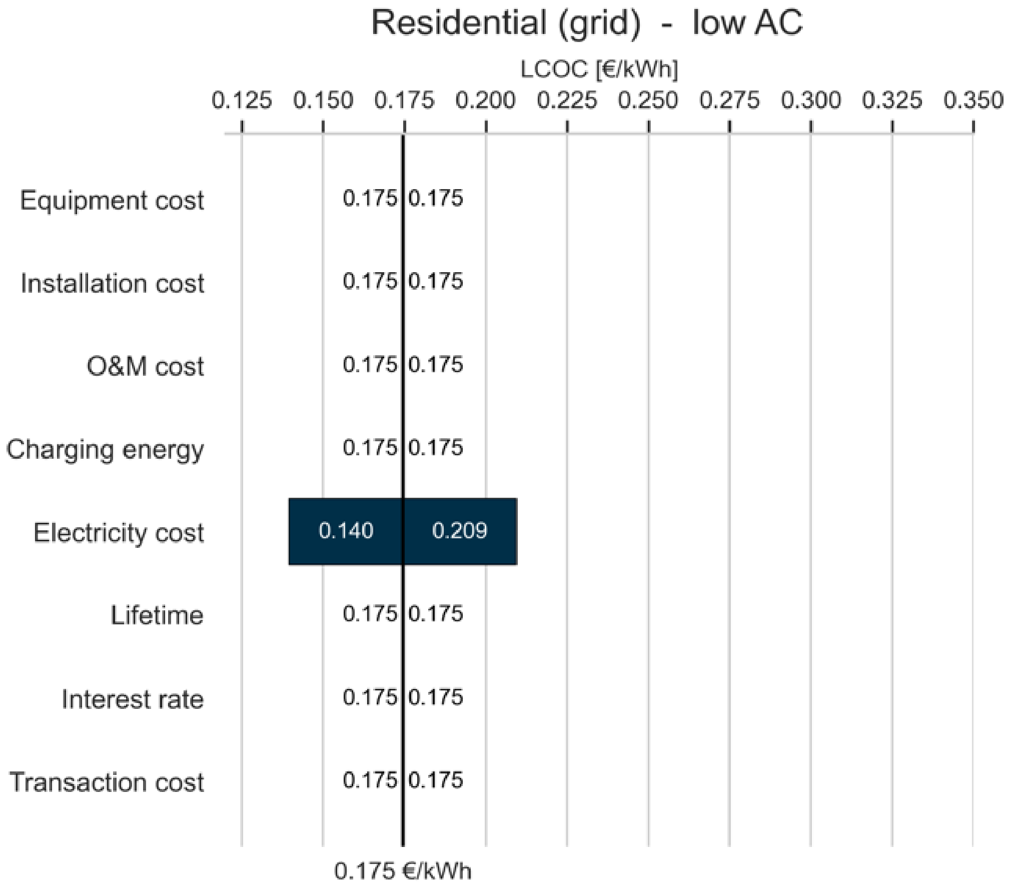

Supplementary Fig. 6. Sensitivity analysis tornado chart for Residential (grid) – low AC. Impact of a +/- 20 % change in the model input parameters (everything else held constant) on the European average LCOC in € per kWh energy charged at Residential (grid) – low AC. The efficiency parameter is not included in the sensitivity analysis because it is primarily a fixed technical property.

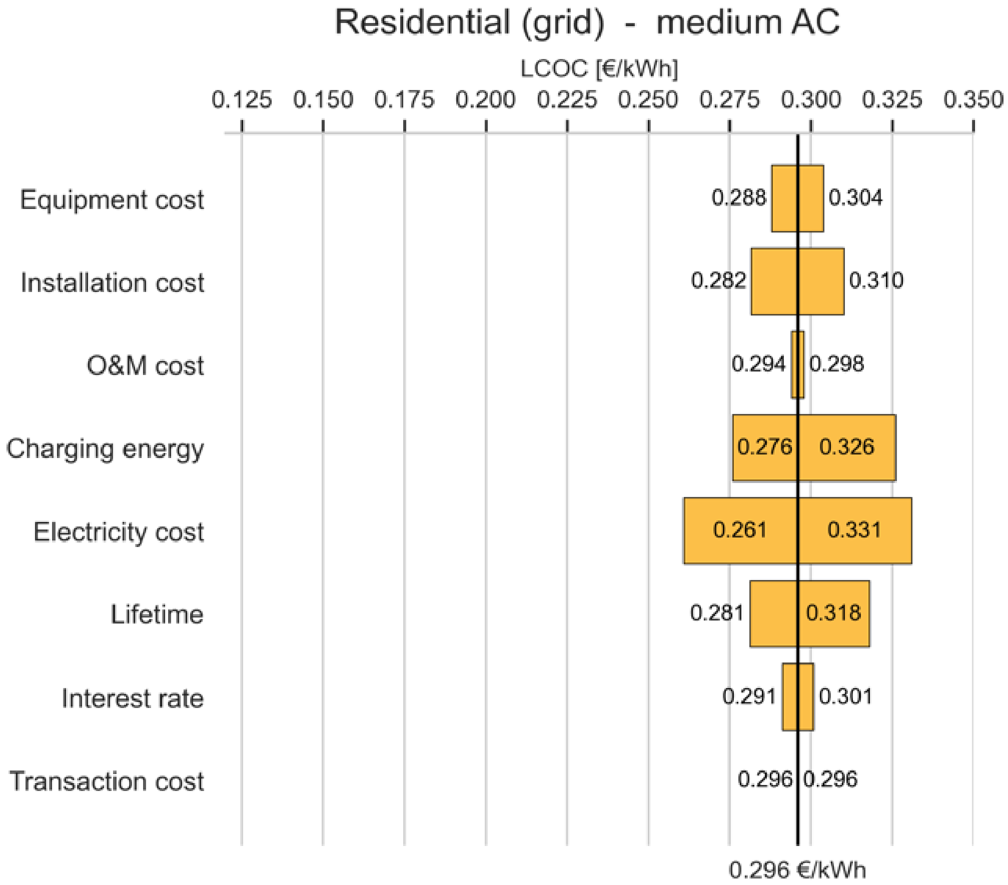

Supplementary Fig. 7. Sensitivity analysis tornado chart for Residential (grid) – medium AC. Impact of a +/- 20 % change in the model input parameters (everything else held constant) on the European average LCOC in € per kWh energy charged at Residential (grid) – medium AC. The efficiency parameter is not included in the sensitivity analysis because it is primarily a fixed technical property.

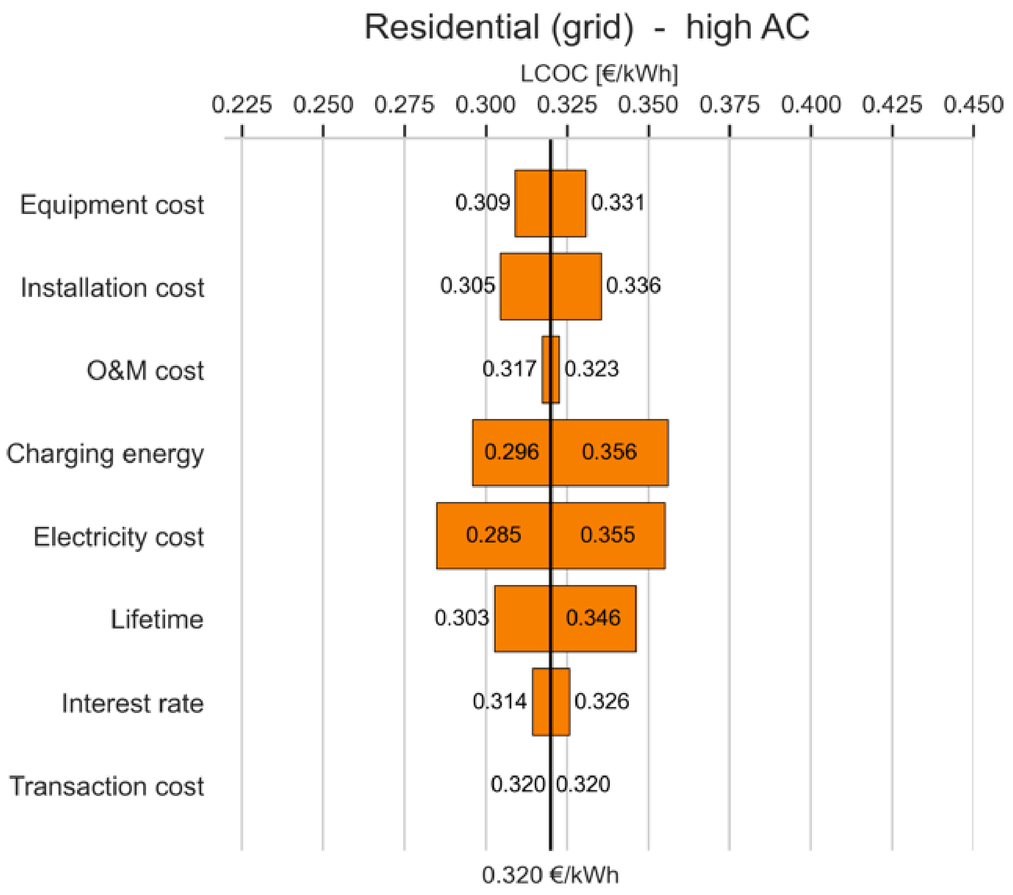

Supplementary Fig. 8. Sensitivity analysis tornado chart for Residential (grid) – high AC. Impact of a +/- 20 % change in the model input parameters (everything else held constant) on the European average LCOC in € per kWh energy charged at Residential (grid) – high AC. Note that for the high AC charging option, the axis is shifted by 0.1 € kWh^{-1}, however it is not scaled. The efficiency parameter is not included in the sensitivity analysis because it is primarily a fixed technical property.

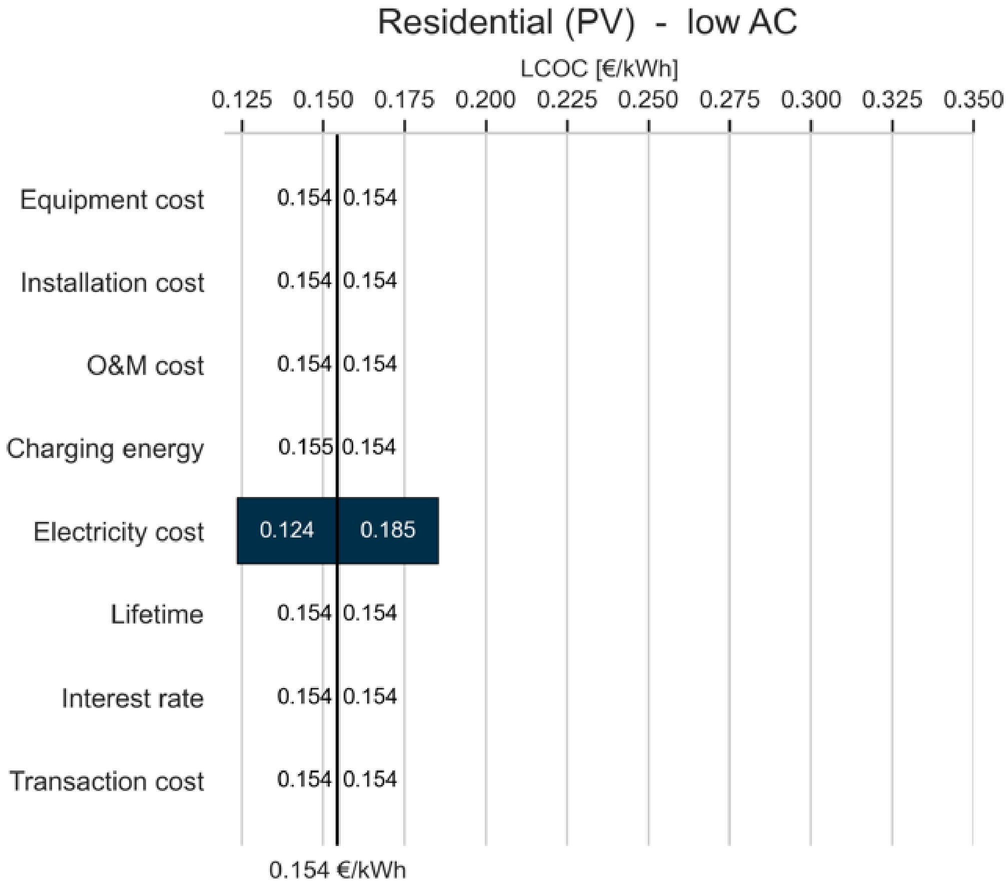

Supplementary Fig. 9. Sensitivity analysis tornado chart for Residential (PV) – low AC. Impact of a +/- 20 % change in the model input parameters (everything else held constant) on the European average LCOC in € per kWh energy charged at Residential (PV) – low AC. The efficiency parameter is not included in the sensitivity analysis because it is primarily a fixed technical property.

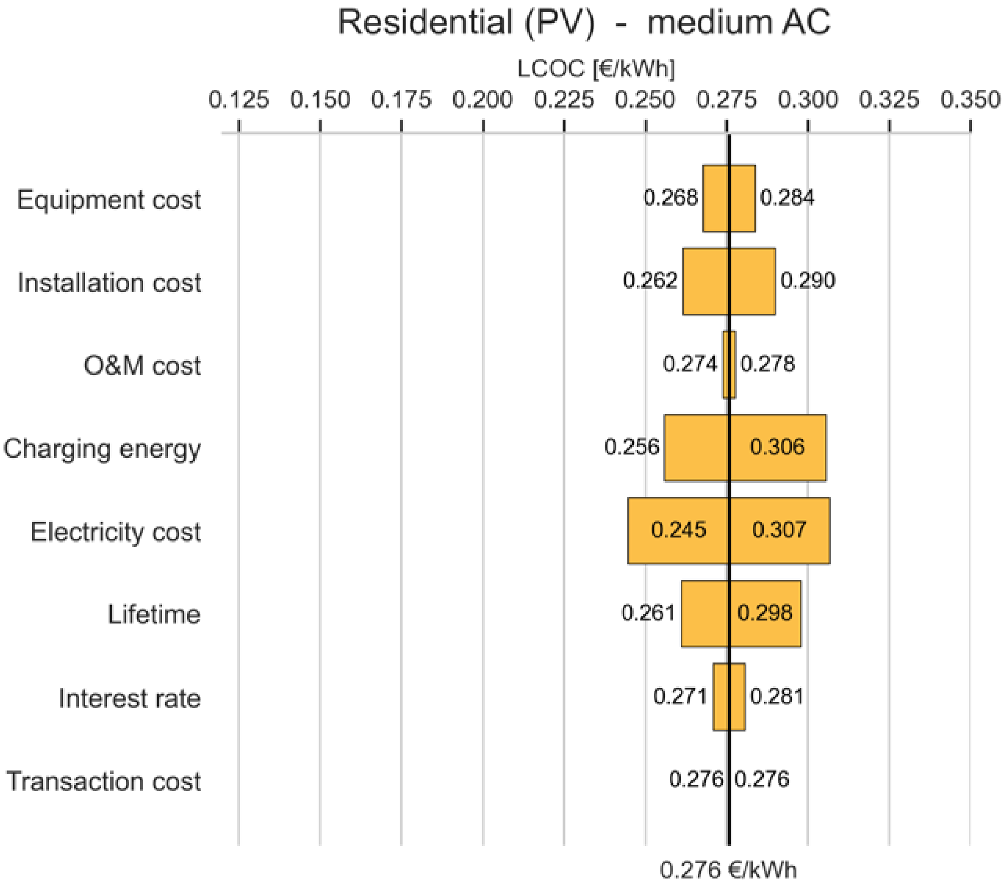

Supplementary Fig. 10. Sensitivity analysis tornado chart for Residential (PV) – medium AC. Impact of a +/- 20 % change in the model input parameters (everything else held constant) on the European average LCOC in € per kWh energy charged at Residential (PV) – medium AC. The efficiency parameter is not included in the sensitivity analysis because it is primarily a fixed technical property.

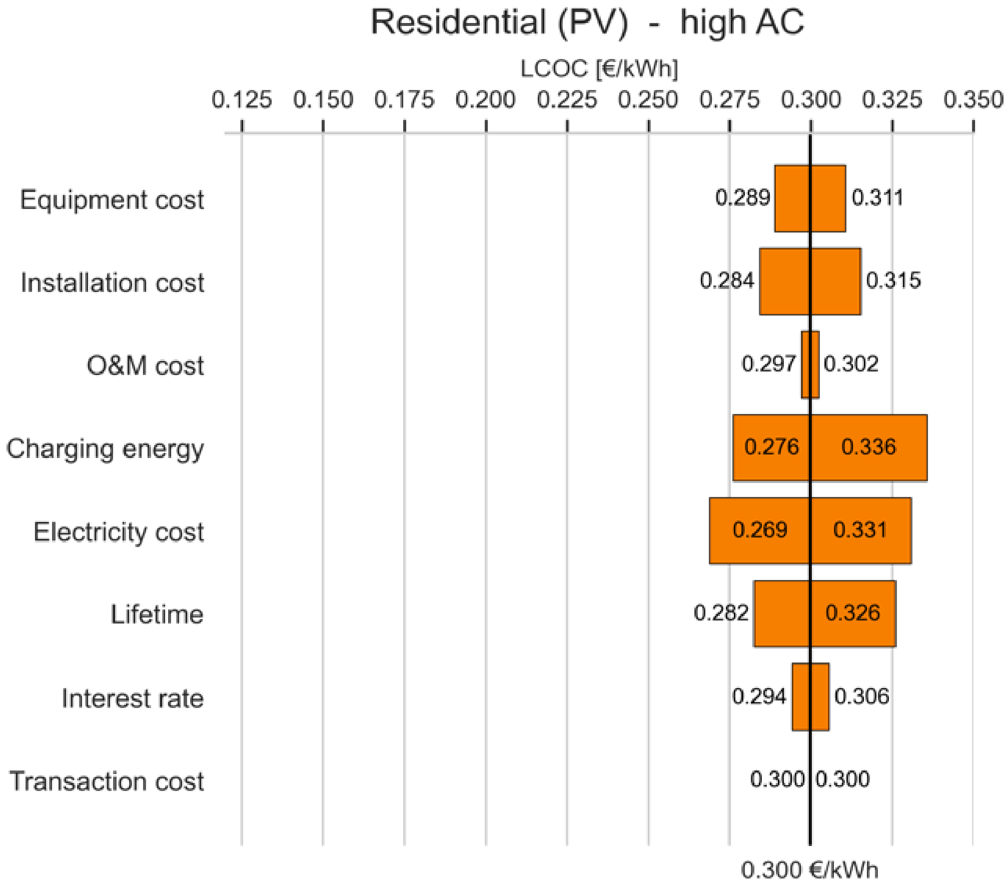

Supplementary Fig. 11. Sensitivity analysis tornado chart for Residential (PV) – high AC. Impact of a +/- 20 % change in the model input parameters (everything else held constant) on the European average LCOC in € per kWh energy charged at Residential (PV) – high AC. The efficiency parameter is not included in the sensitivity analysis because it is primarily a fixed technical property.

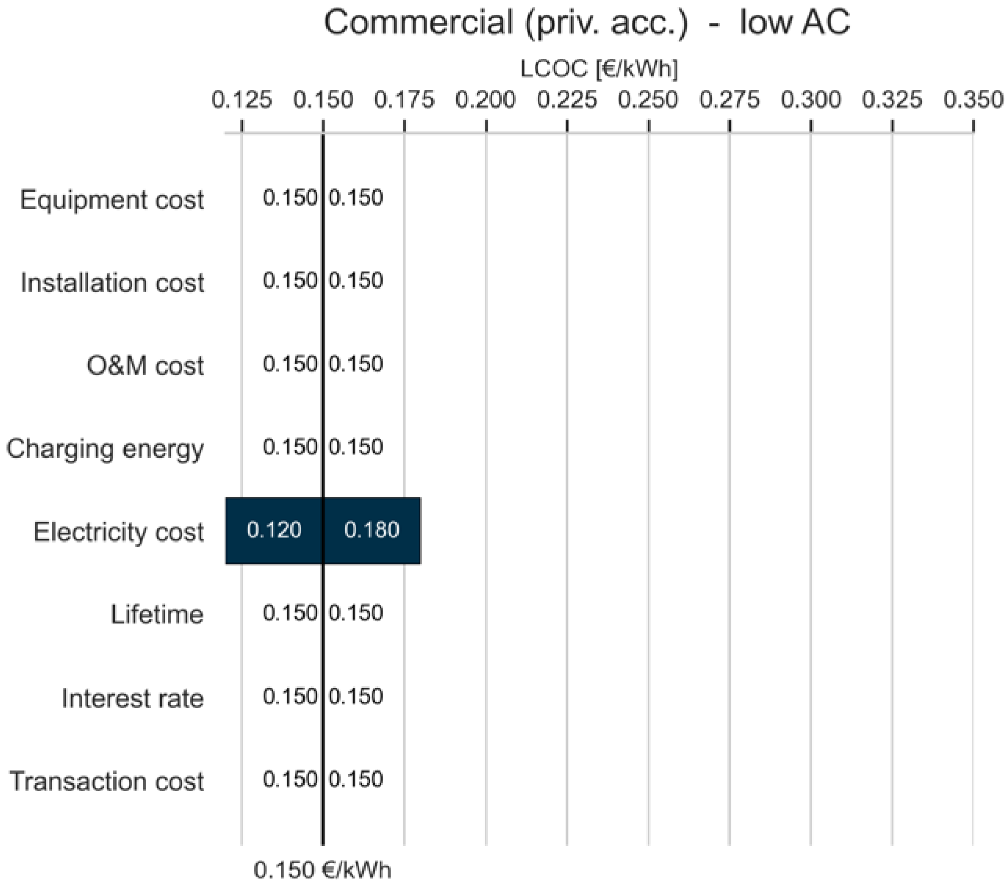

Supplementary Fig. 12. Sensitivity analysis tornado chart for Commercial (priv. acc.) – low AC. Impact of a +/- 20 % change in the model input parameters (everything else held constant) on the European average LCOC in € per kWh energy charged at Commercial (priv. acc.) – low AC. The efficiency parameter is not included in the sensitivity analysis because it is primarily a fixed technical property.

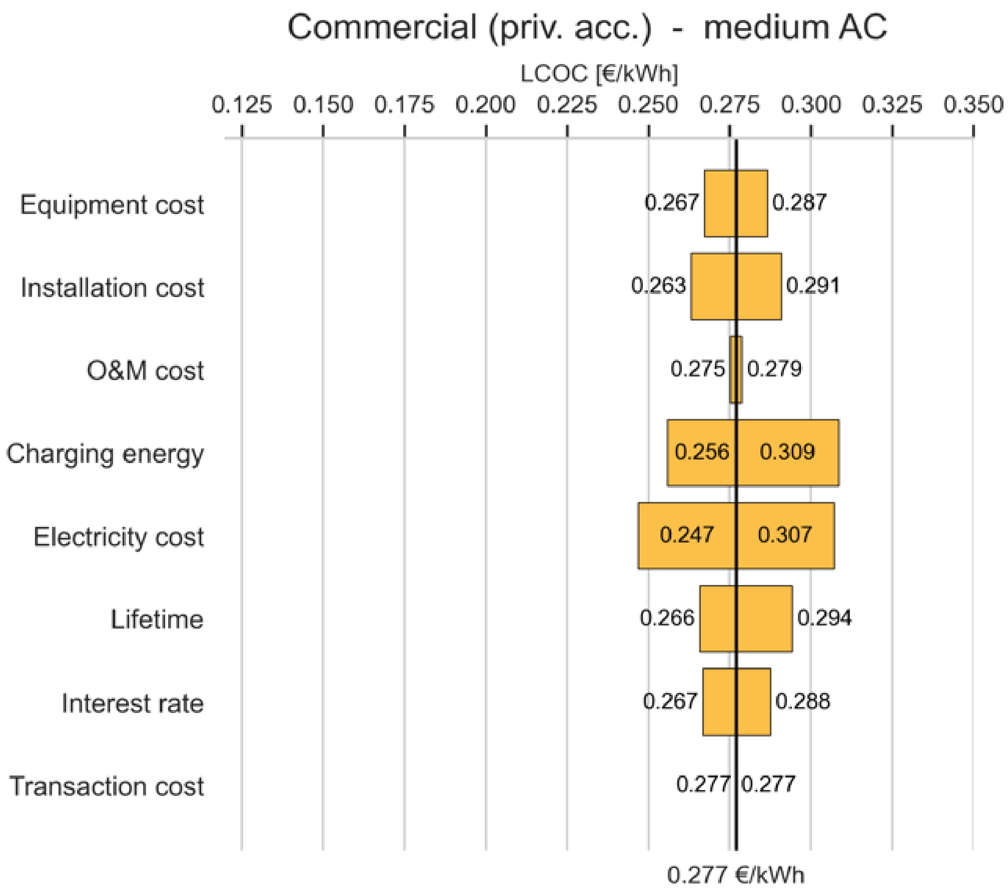

Supplementary Fig. 13. Sensitivity analysis tornado chart for Commercial (priv. acc.) – medium AC. Impact of a +/- 20 % change in the model input parameters (everything else held constant) on the European average LCOC in € per kWh energy charged at Commercial (priv. acc.) – medium AC. The efficiency parameter is not included in the sensitivity analysis because it is primarily a fixed technical property.

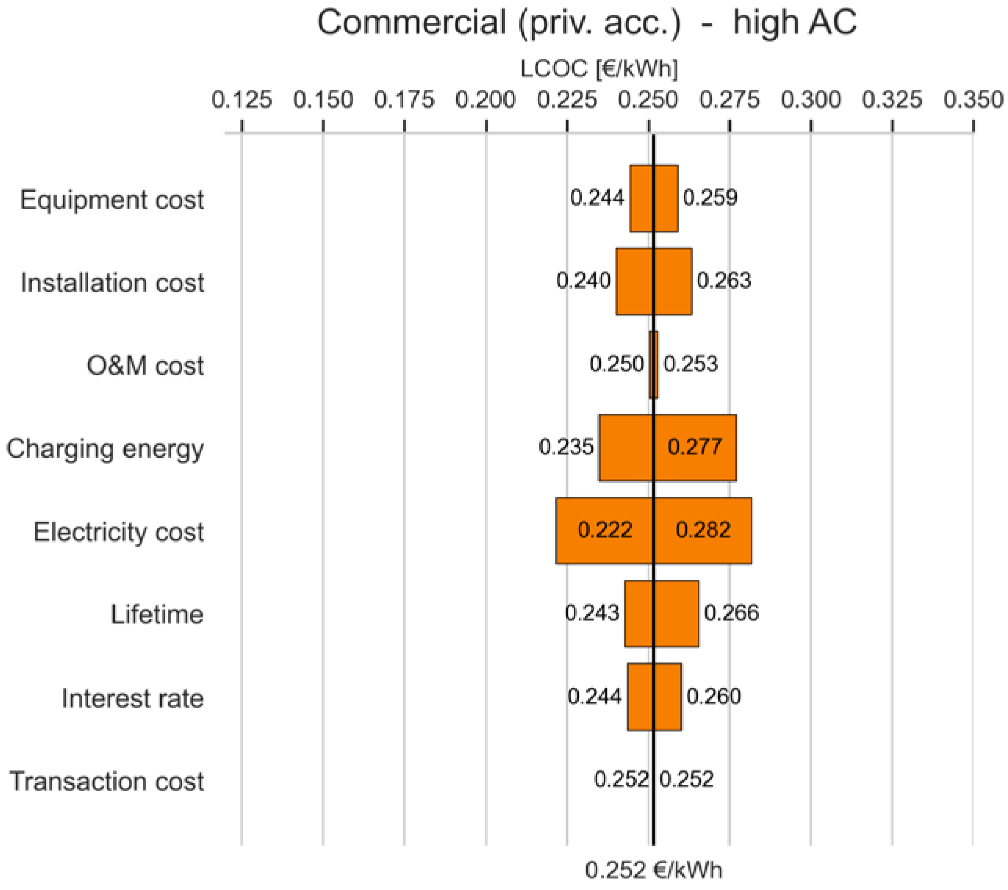

Supplementary Fig. 14. Sensitivity analysis tornado chart for Commercial (priv. acc.) – high AC. Impact of a +/- 20 % change in the model input parameters (everything else held constant) on the European average LCOC in € per kWh energy charged at Commercial (priv. acc.) – high AC. The efficiency parameter is not included in the sensitivity analysis because it is primarily a fixed technical property.

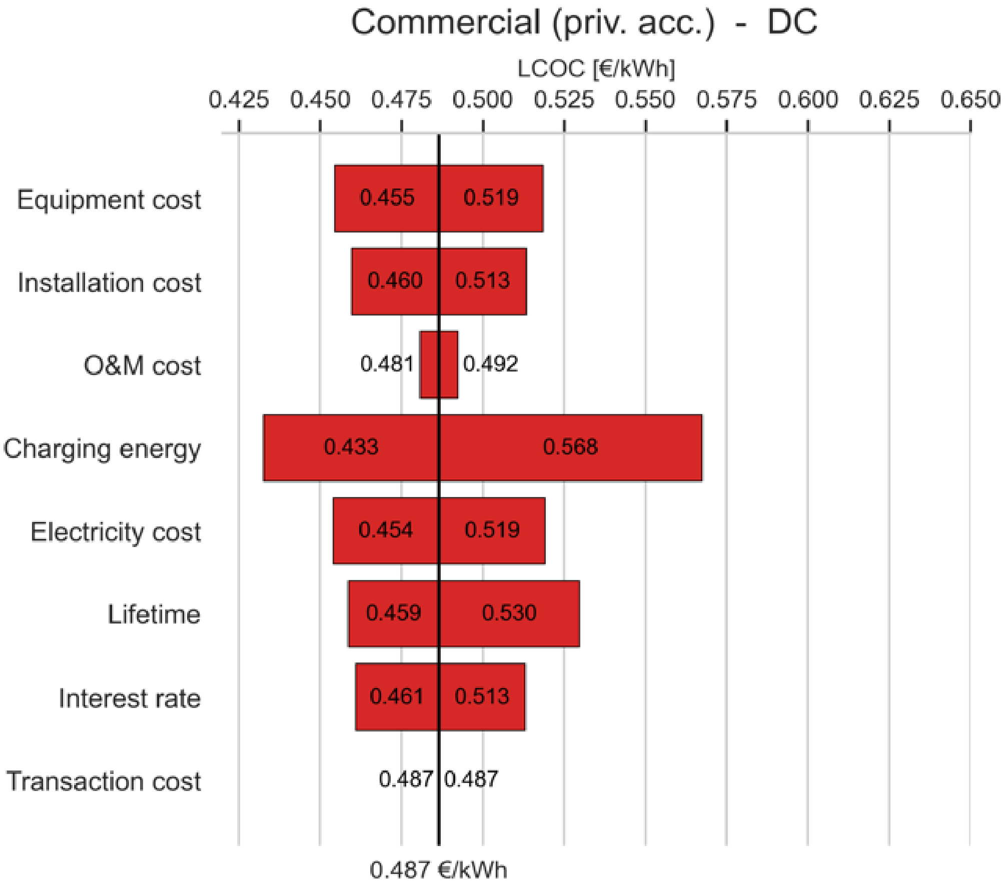

Supplementary Fig. 15. Sensitivity analysis tornado chart for Commercial (priv. acc.) – DC. Impact of a +/- 20 % change in the model input parameters (everything else held constant) on the European average LCOC in € per kWh energy charged at Commercial (priv. acc.) – DC. Note that for the DC charging option, the axis is shifted by 0.3 € kWh^{-1}, however it is not scaled. The efficiency parameter is not included in the sensitivity analysis because it is primarily a fixed technical property.

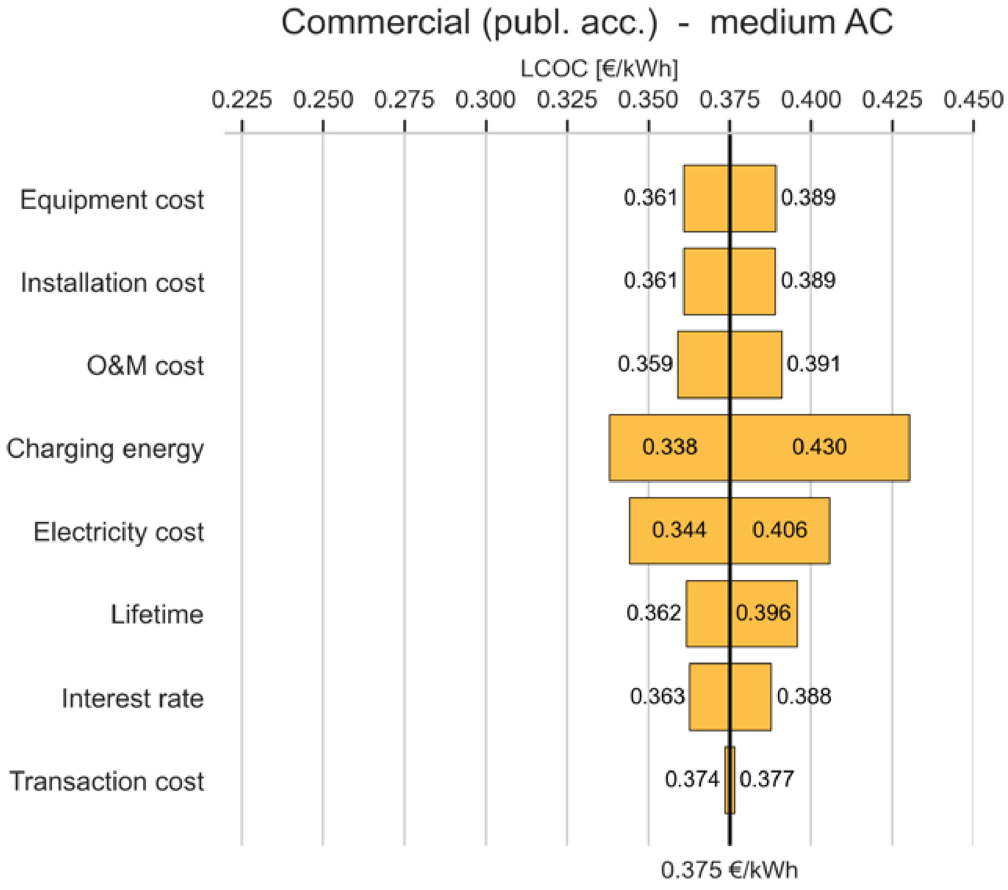

Supplementary Fig. 16. Sensitivity analysis tornado chart for Commercial (priv. acc.) – medium AC. Impact of a +/- 20 % change in the model input parameters (everything else held constant) on the European average LCOC in € per kWh energy charged at Commercial (priv. acc.) – medium AC. The efficiency parameter is not included in the sensitivity analysis because it is primarily a fixed technical property.

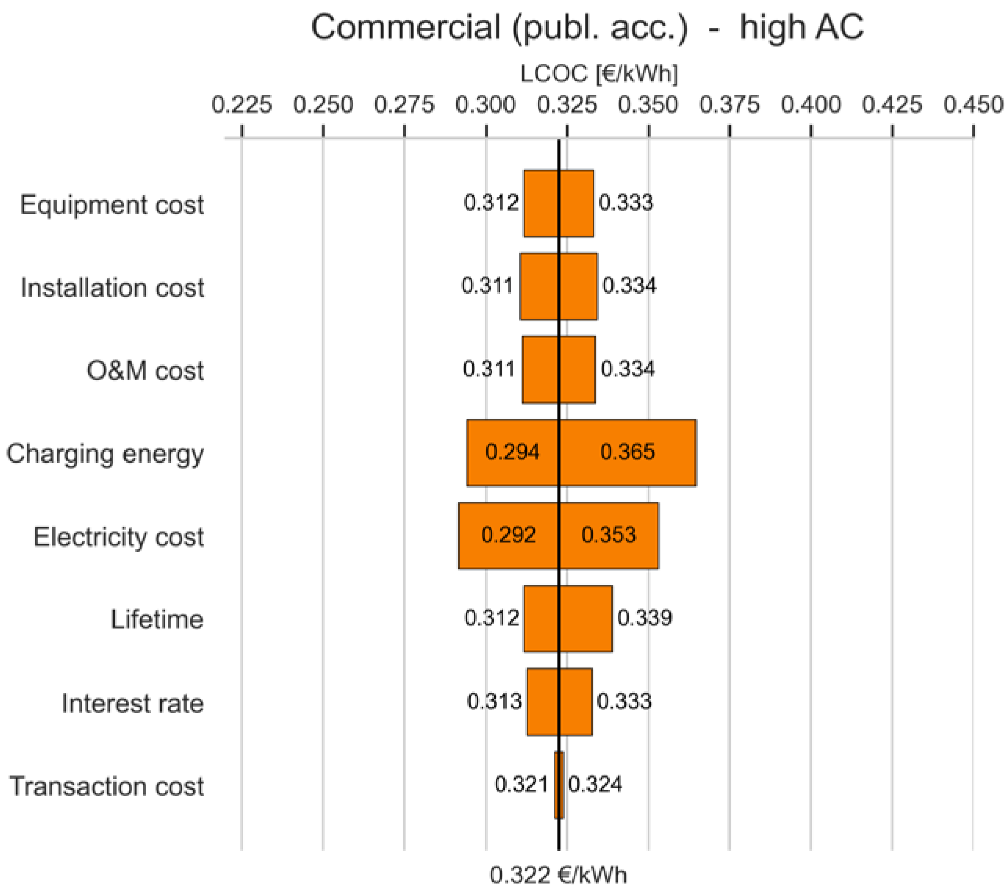

Supplementary Fig. 17. Sensitivity analysis tornado chart for Commercial (priv. acc.) – high AC. Impact of a +/- 20 % change in the model input parameters (everything else held constant) on the European average LCOC in € per kWh energy charged at Commercial (priv. acc.) – high AC. The efficiency parameter is not included in the sensitivity analysis because it is primarily a fixed technical property.

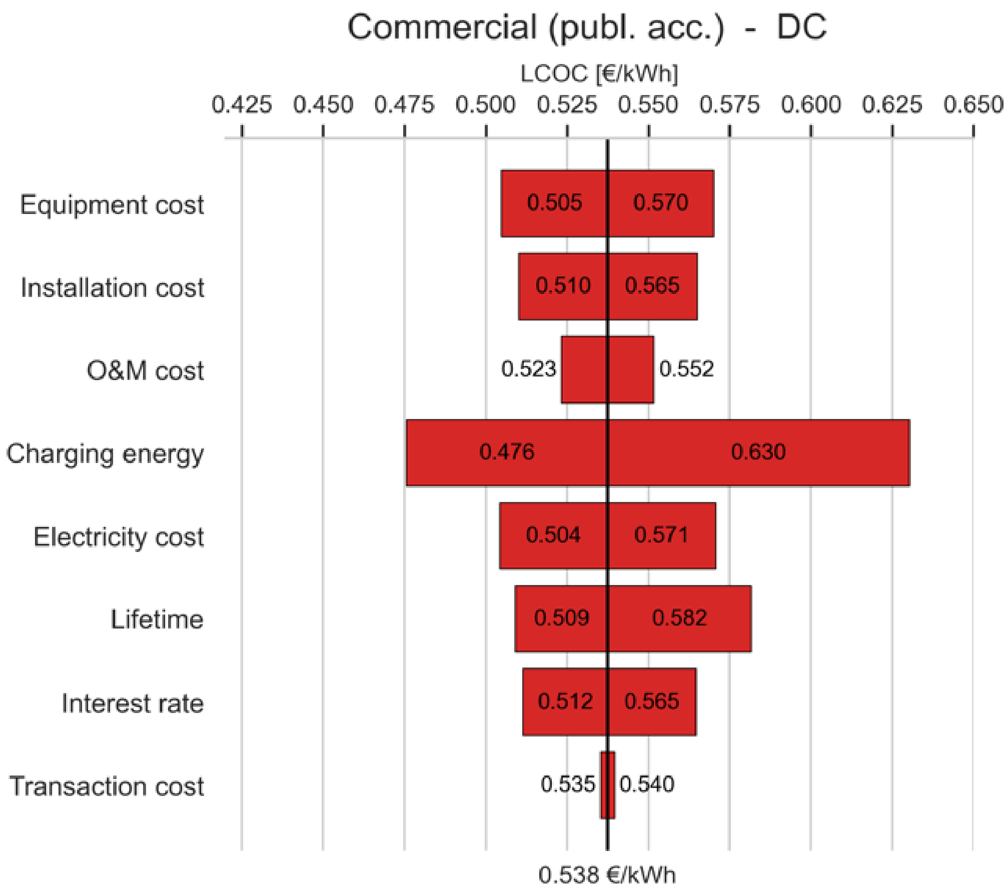

Supplementary Fig. 18. Sensitivity analysis tornado chart for Commercial (priv. acc.) – DC. Impact of a +/- 20 % change in the model input parameters (everything else held constant) on the European average LCOC in € per kWh energy charged at Commercial (priv. acc.) – DC. Note that for the DC charging option, the axis is shifted by 0.2 € kWh^{-1}, however it is not scaled. The efficiency parameter is not included in the sensitivity analysis because it is primarily a fixed technical property.

Supplementary Discussion 2: Impact of electricity price variability

In addition to a general sensitivity analysis of all input parameters (see Supplementary Discussion 1), we also investigate in more detail the impact of electricity price variability. In Supplementary Data 4, we show the impact of a 20 % increase or decrease of electricity prices on the LCOC in all analyzed countries, respectively (also see Supplementary Fig. 19-22)[2]. As EV charging happens mainly overnight, in the main results we assume a residential electricity price 10 % lower than the average price. In Supplementary Data 4, the impact of a 20 and 30 % time-of-use (TOU) reduction on average residential electricity prices on the results is presented as well (also see Supplementary Fig. 23-26).

[2] Eurostat reports the average EU electricity price (all taxes and levies included) in the household consumption band DD (our assumption for home charging sites) for 2019, 2020 and 2021 are 0.198, 0.196, and 0.208 EURO per kWh respectively. For the non-household consumption band IB (our assumption for commercial charging sites), the average EU electricity prices for 2019, 2020 and 2021 are 0.179, 0.183 and 0.196 EURO per kWh respectively.

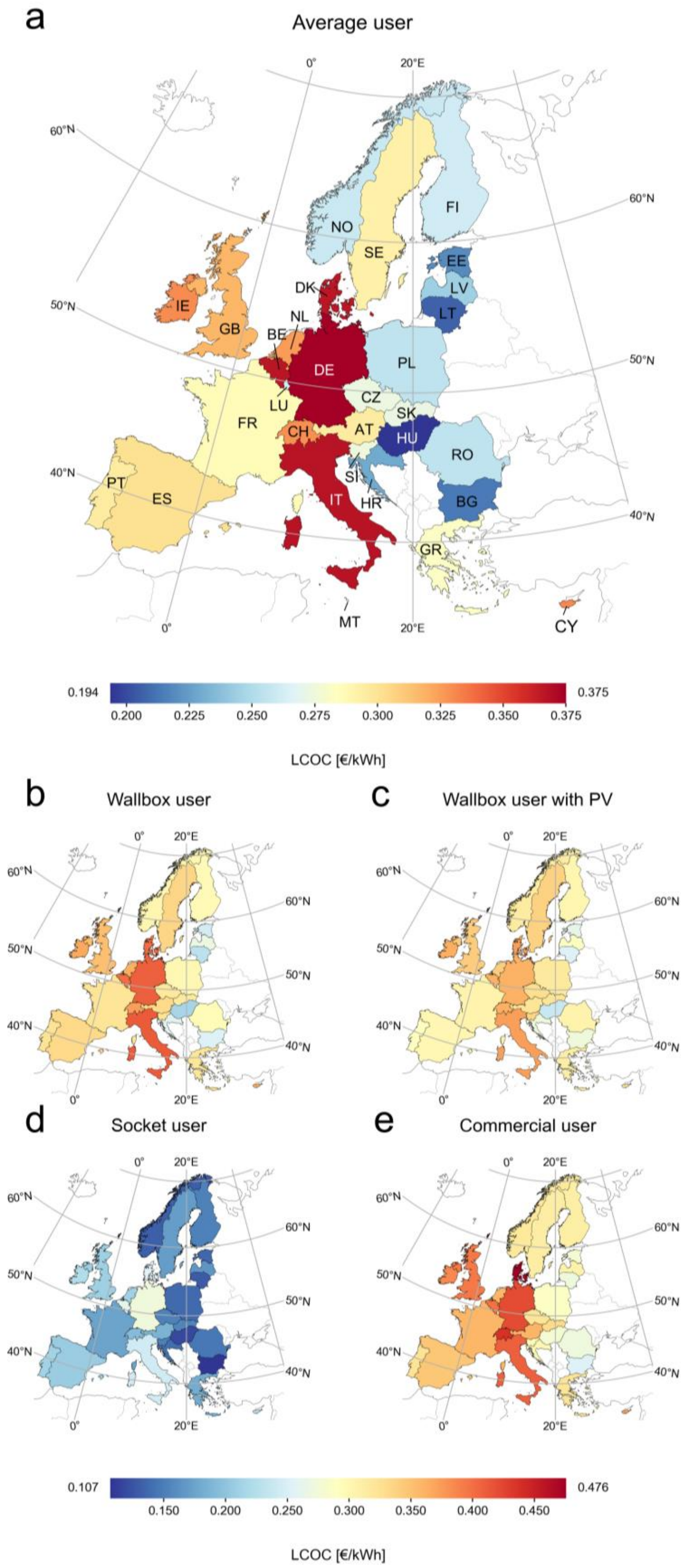

Supplementary Fig. 19. Electricity price sensitivity analysis map for electricity high scenario. Maps for national LCOC in € per kWh of energy charged for five user profiles for the *high* electricity price sensitivity (+20%). **a** *Average user* LCOC results. For full names of countries, please see Supplementary Table 10. **b** *Wallbox user* LCOC results. **c** *Wallbox user with PV* LCOC results. **d** *Socket user* LCOC results. **e** *Commercial user* LCOC results. Results are displayed for all 30 modelled European countries and all units are in € kWh^{-1}. The maps are created with the Cartopy package for Python[58] and use open-source basemap data[59].

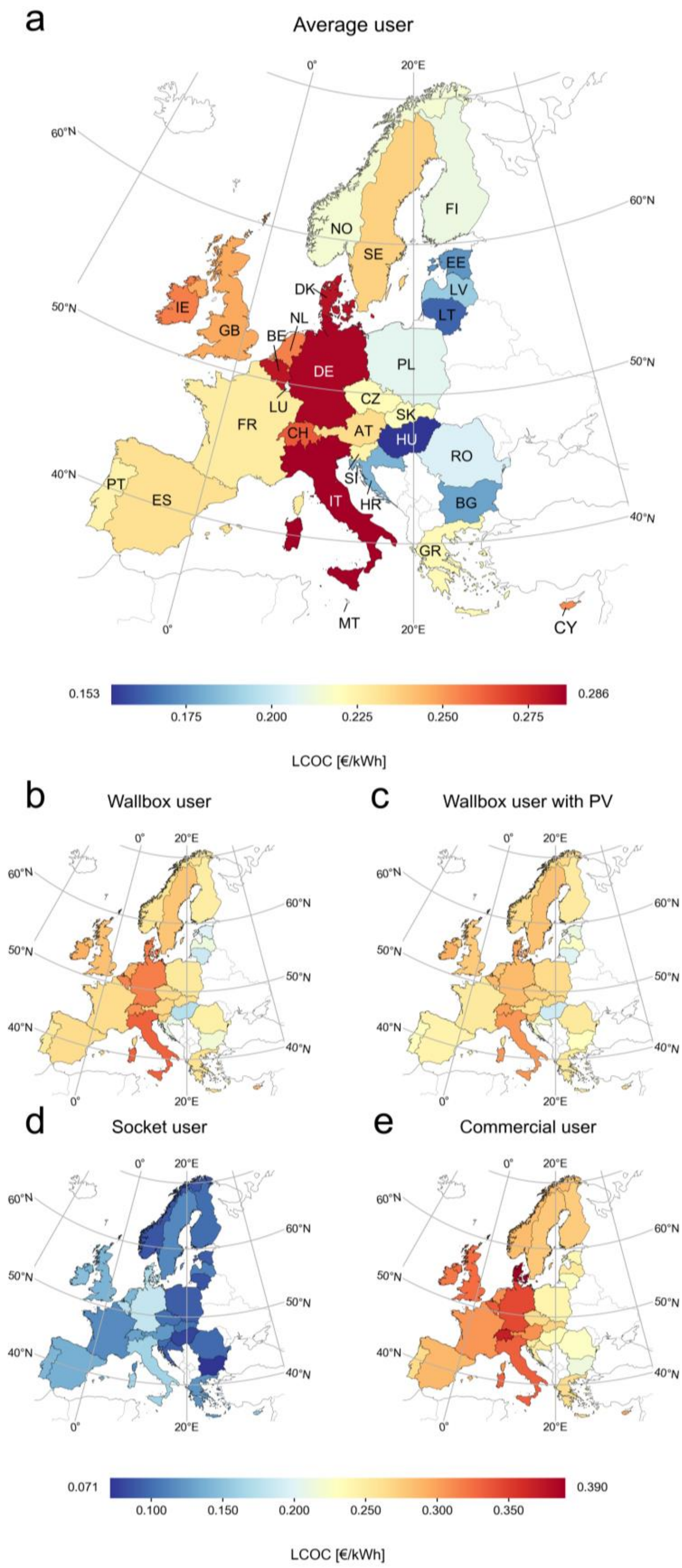

Supplementary Fig. 20. Electricity price sensitivity analysis map for electricity low scenario. Maps for national LCOC in € per kWh of energy charged for five user profiles for the *low* electricity price sensitivity (-20%). **a** *Average user* LCOC results. For full names of countries, please see Supplementary Table 10. **b** *Wallbox user* LCOC results. **c** *Wallbox user with PV* LCOC results. **d** *Socket user* LCOC results. **e** *Commercial user* LCOC results. Results are displayed for all 30 modelled European countries and all units are in € kWh^{-1}. The maps are created with the Cartopy package for Python[58] and use open-source basemap data[59].

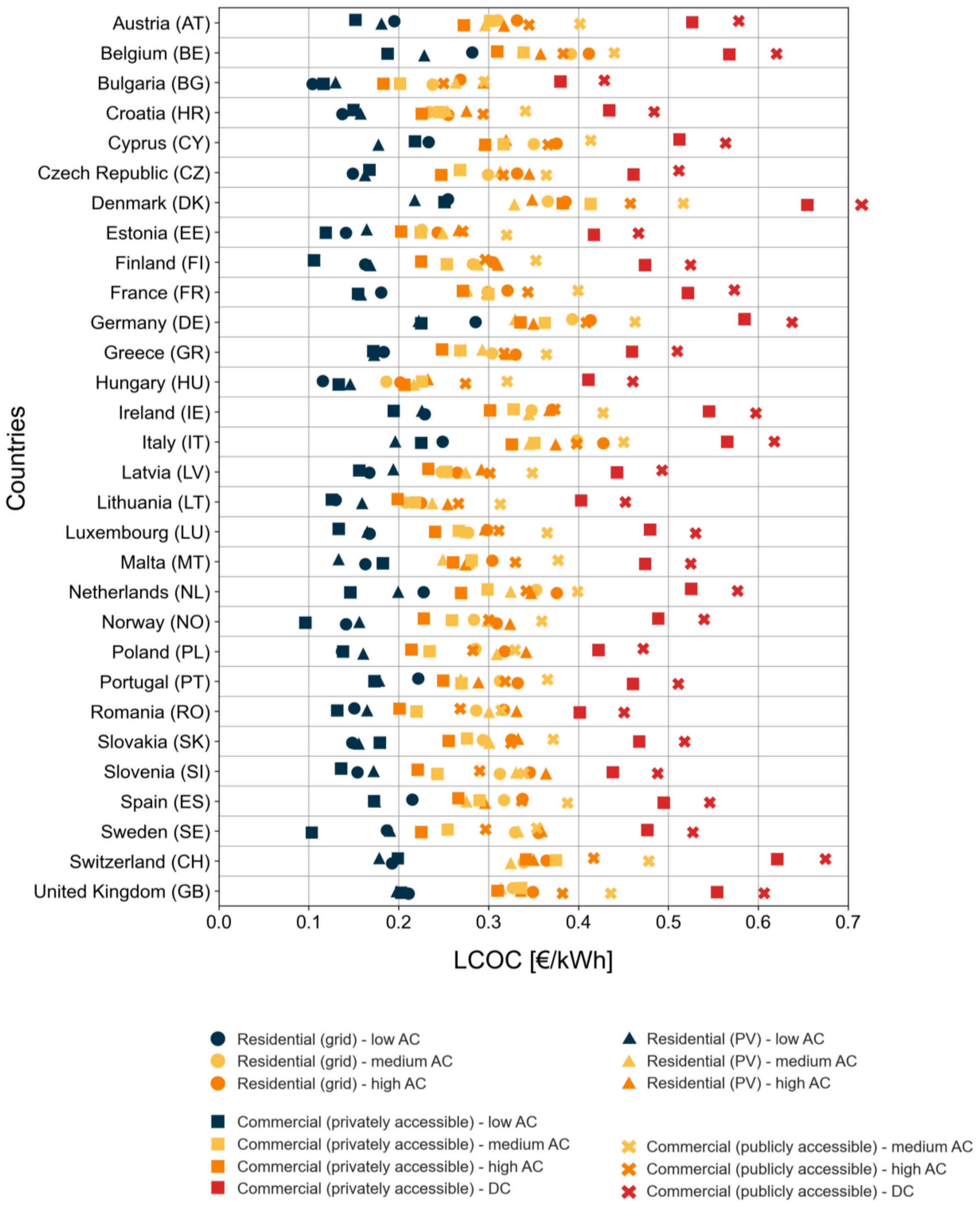

Supplementary Fig. 21. Electricity price sensitivity analysis chart for electricity high scenario. Chart for LCOC in € per kWh of energy charged of different charging options in all analyzed countries. This figure shows the chart results from the *high* electricity price sensitivity (+20 %).

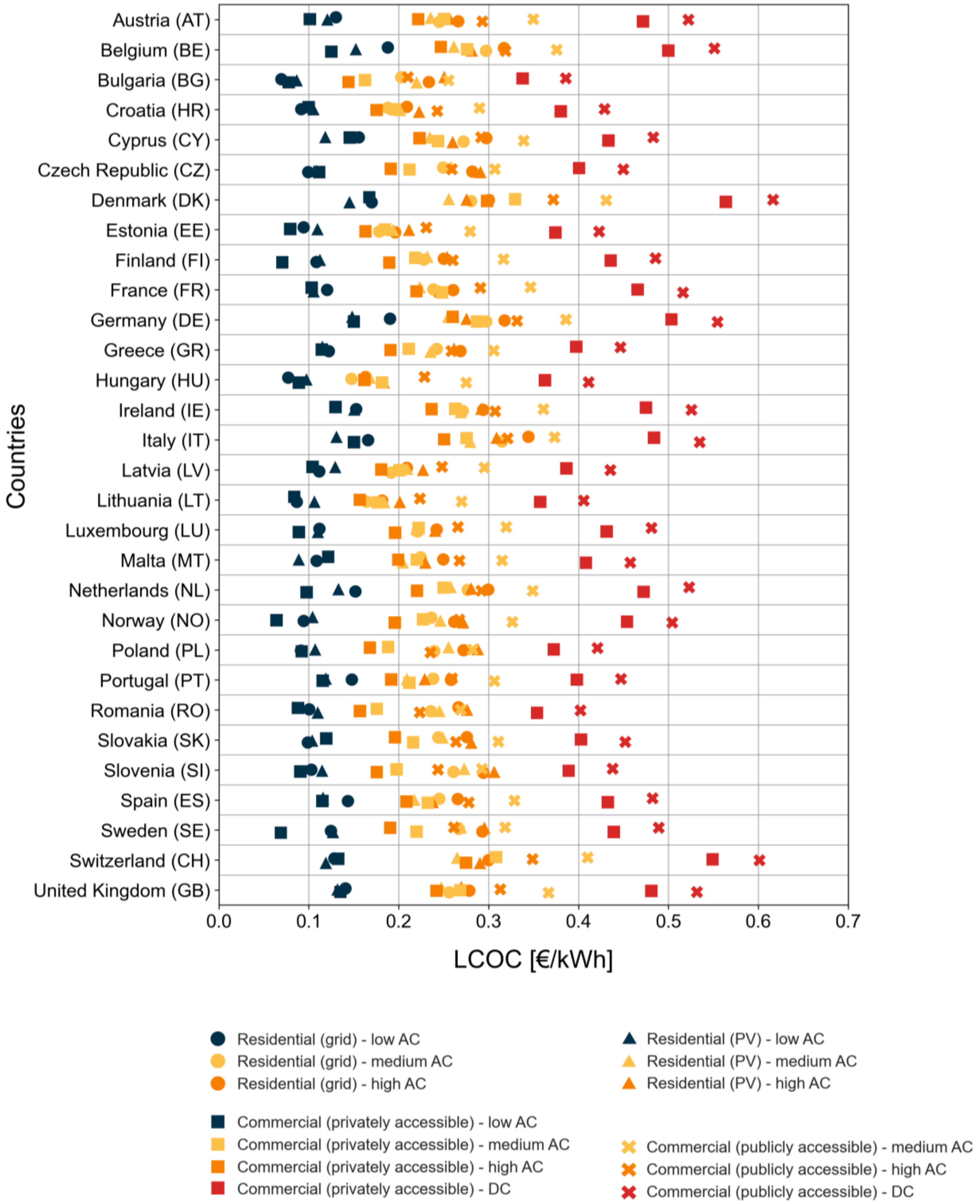

Supplementary Fig. 22. Electricity price sensitivity analysis chart for electricity low scenario. Chart for LCOC in € per kWh of energy charged of different charging options in all analyzed countries. This figure shows the chart results from the *low* electricity price sensitivity (-20 %).

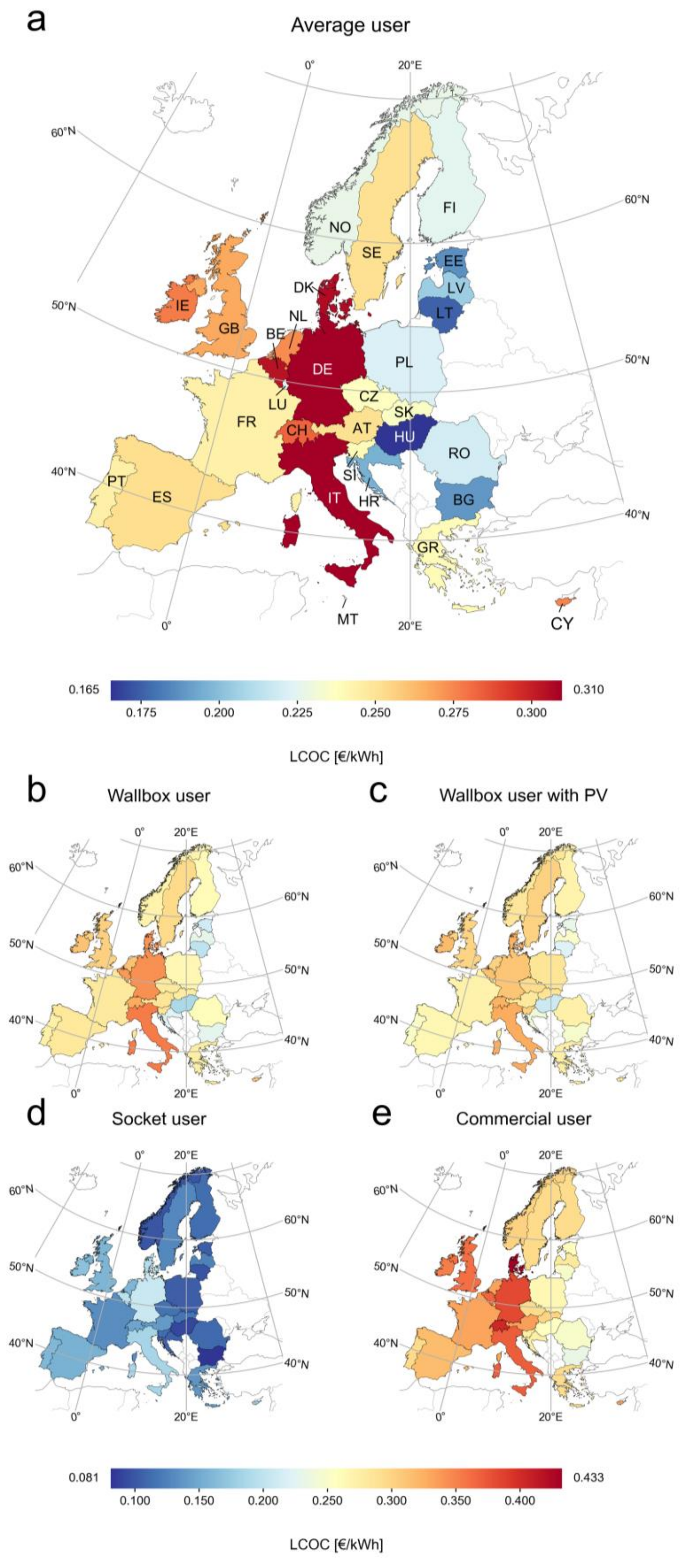

Supplementary Fig. 23. Electricity TOU tariff sensitivity analysis map for TOU tariff medium discount scenario. Maps for national LCOC in € per kWh of energy charged for five user profiles for the *medium* TOU tariff sensitivity (-20 % compared to average residential grid electricity prices) **a** *Average user* LCOC results. For full names of countries, please see Supplementary Table 10. **b** *Wallbox user* LCOC results. **c** *Wallbox user with PV* LCOC results. **d** *Socket user* LCOC results. **e** *Commercial user* LCOC results. Results are displayed for all 30 modelled European countries and all units are in € kWh^{-1}. The maps are created with the Cartopy package for Python[58] and use open-source basemap data[59].

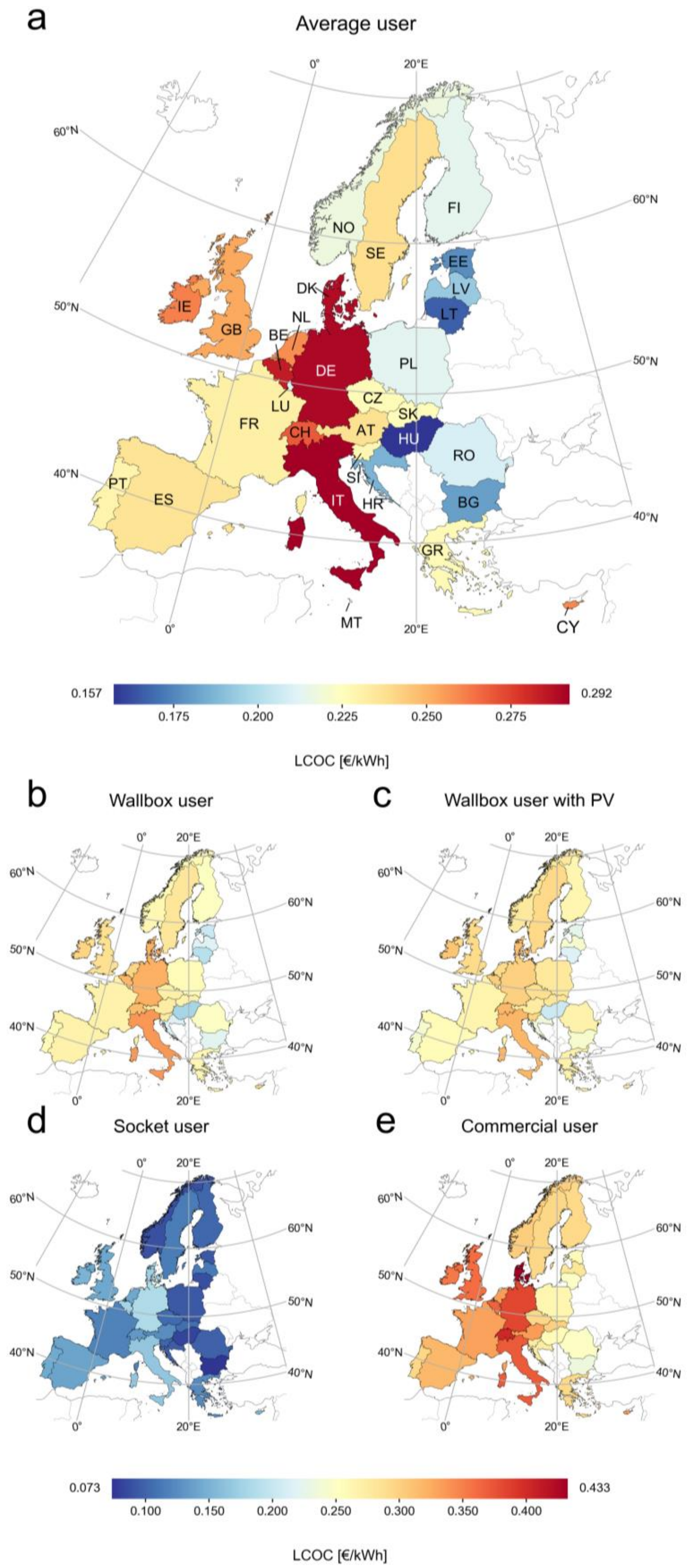

Supplementary Fig. 24. Electricity TOU tariff sensitivity analysis map for TOU tariff high discount scenario. Maps for national LCOC in € per kWh of energy charged for five user profiles for the *high* TOU tariff sensitivity (-30 % compared to average residential grid electricity prices) **a** *Average user* LCOC results. For full names of countries, please see Supplementary Table 10. **b** *Wallbox user* LCOC results. **c** *Wallbox user with PV* LCOC results. **d** *Socket user* LCOC results. **e** *Commercial user* LCOC results. Results are displayed for all 30 modelled European countries and all units are in € kWh^{-1}. The maps are created with the Cartopy package for Python[58] and use open-source basemap data[59].

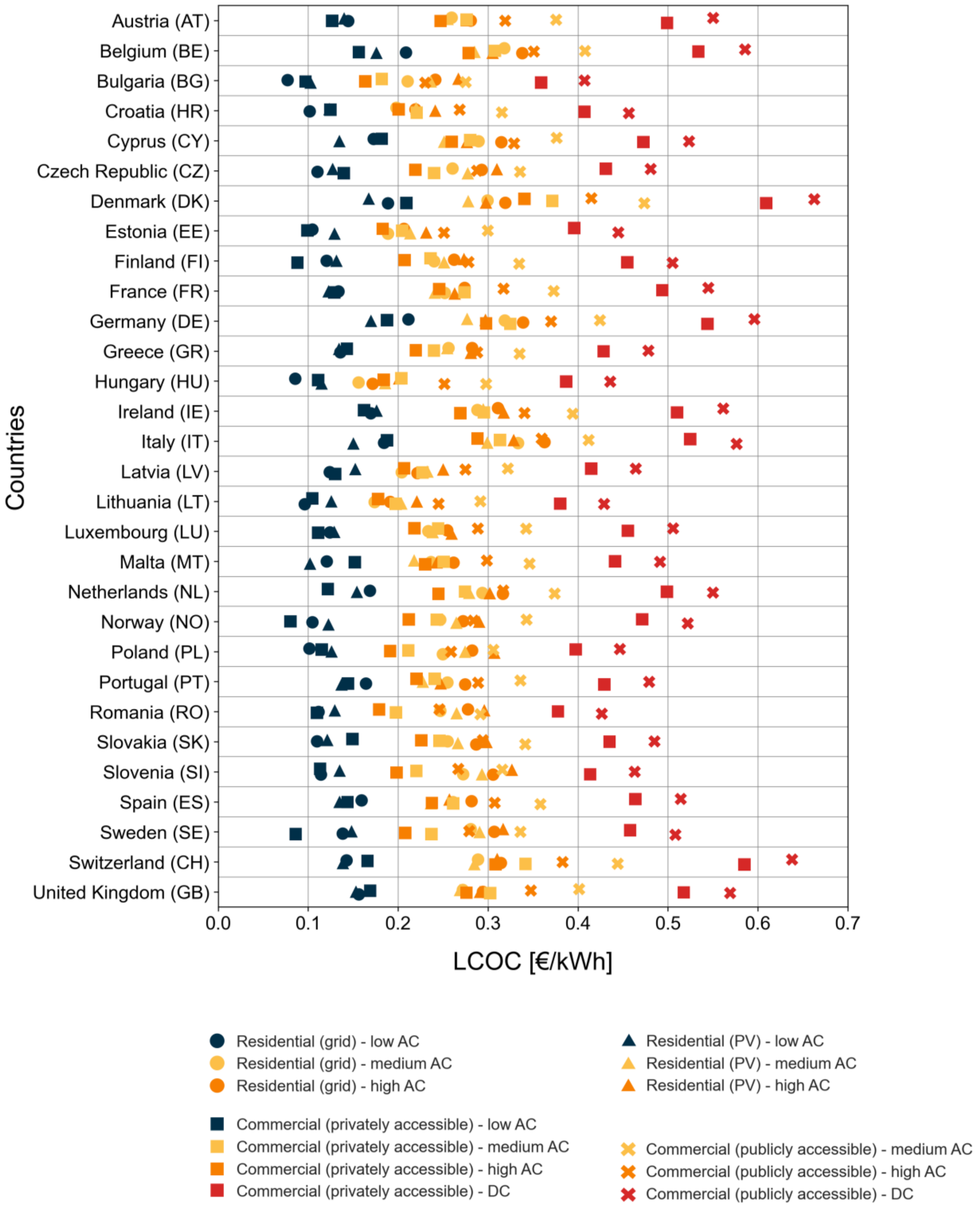

Supplementary Fig. 25. Electricity TOU tariff sensitivity analysis chart for TOU tariff medium discount scenario. Chart for LCOC in € per kWh of energy charged of different charging options in all analyzed countries. This figure shows the results from the *medium* TOU tariff sensitivity (-20 % compared to average residential grid electricity prices).

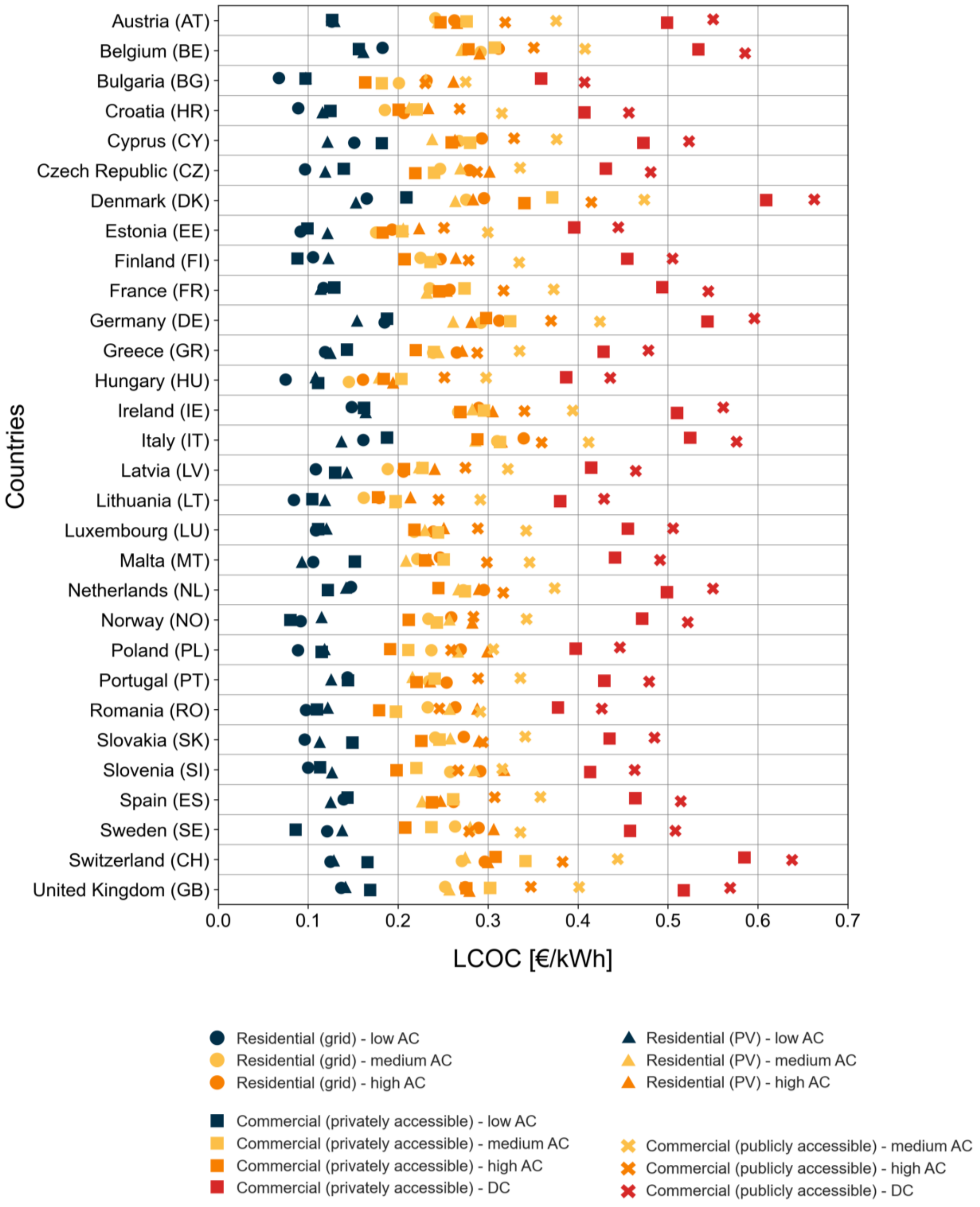

Supplementary Figure 26. Electricity TOU tariff sensitivity analysis chart for TOU tariff high discount scenario. Chart LCOC in € per kWh of energy charged of different charging options in all analyzed countries. This figure shows the results from the *high* TOU tariff sensitivity (-30 % compared to average residential grid electricity prices).

Supplementary Table 17. National LCOC and European average LCOC of the five user profiles (€ kWh^{-1}).

	Average user	Wallbox user	Wallbox user with PV	Commercial user	Socket user
**Austria**	0.265	0.300	0.291	0.334	0.155
**Belgium**	0.324	0.358	0.324	0.367	0.219
**Bulgaria**	0.196	0.235	0.251	0.233	0.089
**Croatia**	0.206	0.235	0.248	0.274	0.117
**Cyprus**	0.292	0.327	0.292	0.335	0.192
**Czech Republic**	0.247	0.292	0.300	0.294	0.127
**Denmark**	0.324	0.358	0.335	0.433	0.212
**Estonia**	0.197	0.223	0.237	0.258	0.114
**Finland**	0.237	0.273	0.277	0.293	0.126
**France**	0.257	0.293	0.279	0.332	0.146
**Germany**	0.330	0.363	0.323	0.383	0.228
**Greece**	0.252	0.288	0.282	0.293	0.151
**Hungary**	0.173	0.195	0.214	0.256	0.099
**Ireland**	0.293	0.329	0.327	0.353	0.185
**Italy**	0.327	0.371	0.338	0.370	0.204
**Latvia**	0.217	0.242	0.258	0.280	0.138
**Lithuania**	0.185	0.209	0.227	0.250	0.108
**Luxembourg**	0.237	0.270	0.269	0.301	0.134
**Malta**	0.240	0.275	0.256	0.305	0.139
**Netherlands**	0.290	0.328	0.310	0.333	0.176
**Norway**	0.237	0.280	0.289	0.301	0.110
**Poland**	0.232	0.275	0.290	0.264	0.114
**Portugal**	0.260	0.288	0.261	0.294	0.177
**Romania**	0.230	0.270	0.279	0.250	0.122
**Slovakia**	0.246	0.289	0.293	0.300	0.129
**Slovenia**	0.250	0.296	0.308	0.274	0.125
**Spain**	0.267	0.298	0.272	0.317	0.172
**Sweden**	0.264	0.307	0.309	0.294	0.142
**Switzerland**	0.296	0.341	0.332	0.403	0.162
**United Kingdom**	0.282	0.317	0.309	0.360	0.175
**European average**	0.278	0.315	0.299	0.334	0.170

Supplementary Table 18. National and European average charging cost for the five user profiles compared to fuel costs of conventional gasoline cars in 2019[60] (€ 100km^{-1}). An efficient gasoline car is assumed to consume 5 L 100km^{-1}, an inefficient car 10 L 100km^{-1}, representing around 90 % of the fleet.

	Average user	Wallbox user	Wallbox user with PV	Commercial user	Socket user	Efficient gasoline car	Inefficient gasoline car
**Austria**	4.60	5.22	5.06	5.82	2.70	6.19	12.38
**Belgium**	5.63	6.22	5.64	6.38	3.81	6.99	13.98
**Bulgaria**	3.41	4.10	4.38	4.06	1.55	5.46	10.93
**Croatia**	3.58	4.09	4.31	4.76	2.03	6.55	13.10
**Cyprus**	5.08	5.69	5.08	5.82	3.34	5.94	11.88
**Czech Republic**	4.31	5.08	5.22	5.11	2.21	6.28	12.55
**Denmark**	5.63	6.23	5.83	7.53	3.68	7.94	15.88
**Estonia**	3.43	3.88	4.13	4.49	1.98	6.70	13.40
**Finland**	4.12	4.75	4.82	5.10	2.19	7.58	15.15
**France**	4.47	5.10	4.85	5.77	2.54	7.54	15.08
**Germany**	5.74	6.31	5.62	6.67	3.97	7.01	14.03
**Greece**	4.39	5.02	4.90	5.11	2.63	8.05	16.10
**Hungary**	3.02	3.40	3.73	4.46	1.73	5.83	11.65
**Ireland**	5.10	5.73	5.69	6.14	3.22	7.08	14.15
**Italy**	5.69	6.46	5.88	6.45	3.54	7.89	15.78
**Latvia**	3.77	4.20	4.50	4.88	2.39	6.28	12.55
**Lithuania**	3.21	3.63	3.95	4.34	1.87	5.78	11.55
**Luxembourg**	4.12	4.70	4.67	5.23	2.33	6.11	12.23
**Malta**	4.18	4.78	4.45	5.30	2.42	6.93	13.85
**Netherlands**	5.04	5.71	5.40	5.79	3.07	8.23	16.45
**Norway**	4.12	4.87	5.03	5.24	1.92	8.56	17.13
**Poland**	4.03	4.79	5.05	4.60	1.99	5.81	11.63
**Portugal**	4.53	5.01	4.53	5.12	3.08	7.48	14.95
**Romania**	4.00	4.70	4.85	4.34	2.13	5.81	11.63
**Slovakia**	4.28	5.03	5.10	5.21	2.24	6.60	13.20
**Slovenia**	4.35	5.16	5.35	4.77	2.18	6.43	12.85
**Spain**	4.64	5.18	4.73	5.51	3.00	6.48	12.95
**Sweden**	4.60	5.35	5.38	5.12	2.47	7.46	14.93
**Switzerland**	5.15	5.92	5.77	7.01	2.81	6.98	13.95
**United Kingdom**	4.91	5.52	5.37	6.26	3.04	7.18	14.35
**European average**	4.84	5.47	5.21	5.80	2.95	7.00	14.00

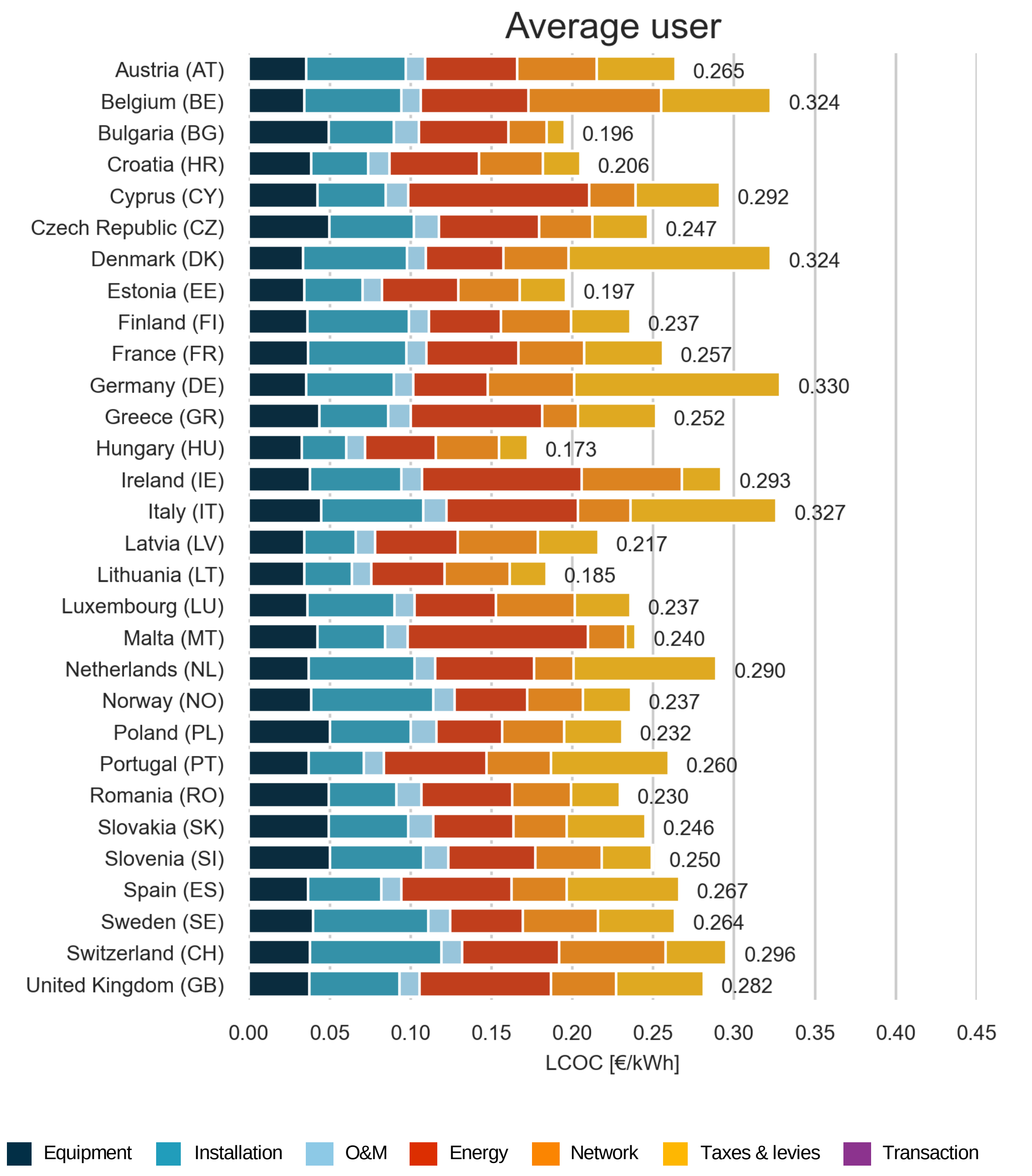

Supplementary Fig. 27. National LCOC in € per kWh of energy charged for user profile *Average user* by components. Each bar gives the total LCOC in € kWh^{-1} for a given country segmented into the main LCOC parameters (equipment, installation, O&M, energy, network, taxes & levies, transaction fees).

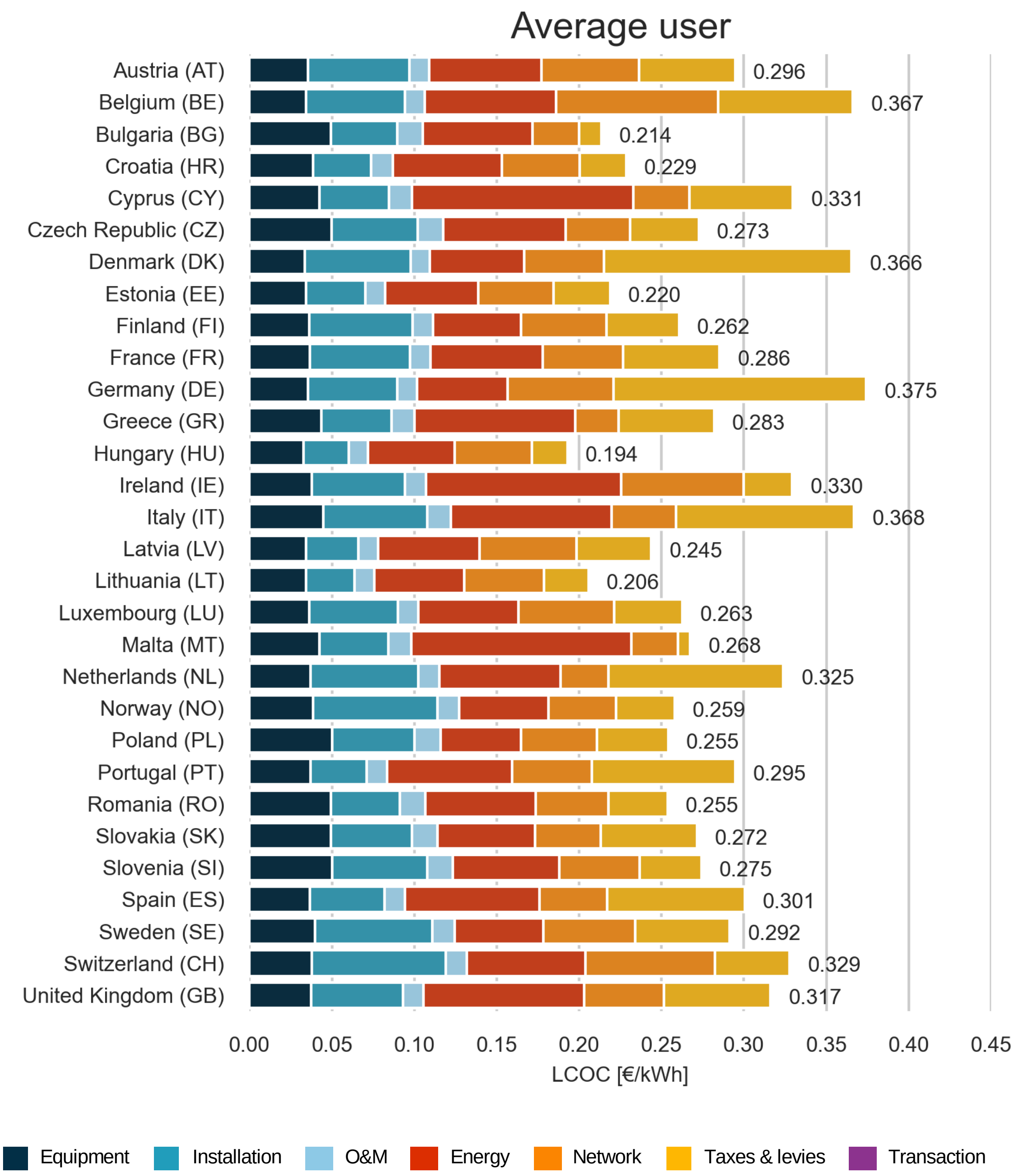

Supplementary Fig. 27. National LCOC in € per kWh of energy charged for user profile *Average user* by components for the *high* electricity price sensitivity (+20 %). Each bar gives the total LCOC in € kWh^{-1} for a given country segmented into the main LCOC parameters (equipment, installation, O&M, energy, network, taxes & levies, transaction fees).

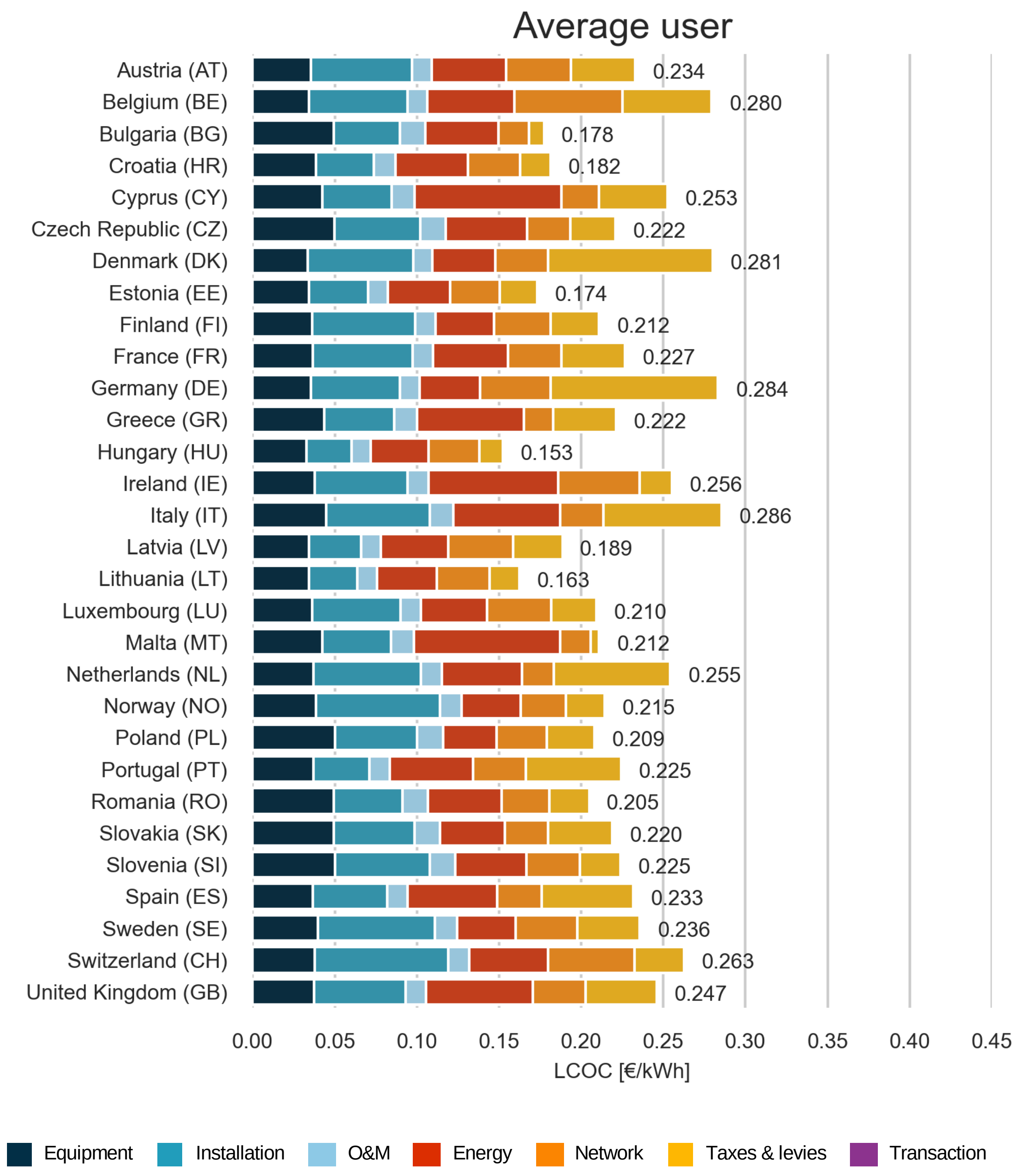

Supplementary Fig. 28. National LCOC in € per kWh of energy charged for user profile *Average user* by components for the *low* electricity price sensitivity (-20 %). Each bar gives the total LCOC in € kWh^{-1} for a given country segmented into the main LCOC parameters (equipment, installation, O&M, energy, network, taxes & levies, transaction fees).

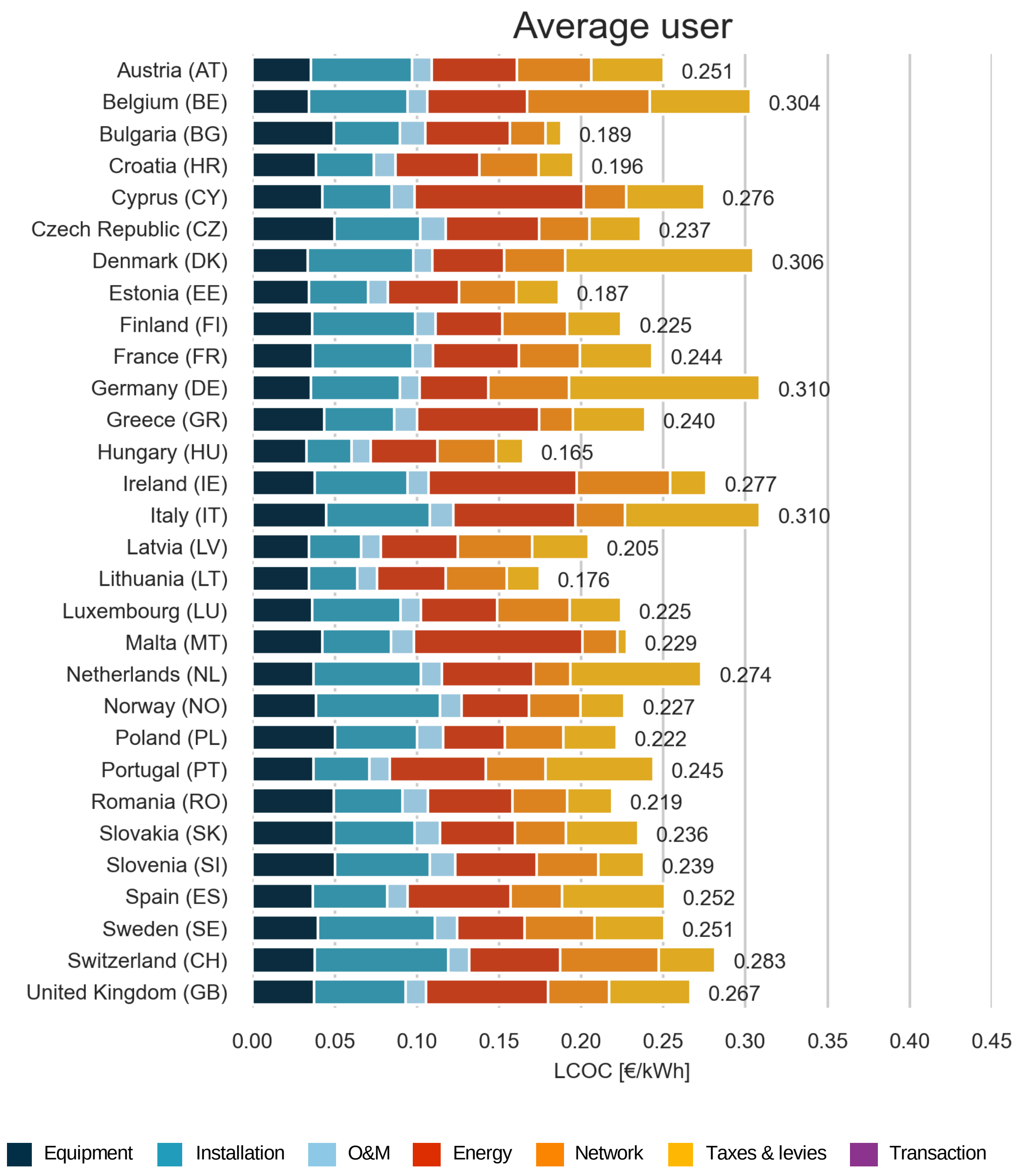

Supplementary Fig. 29. National LCOC in € per kWh of energy charged for user profile *Average user* by components for the *medium* TOU tariff sensitivity (-20 % compared to average residential grid electricity prices). Each bar gives the total LCOC in € kWh^{-1} for a given country segmented into the main LCOC parameters (equipment, installation, O&M, energy, network, taxes & levies, transaction fees).

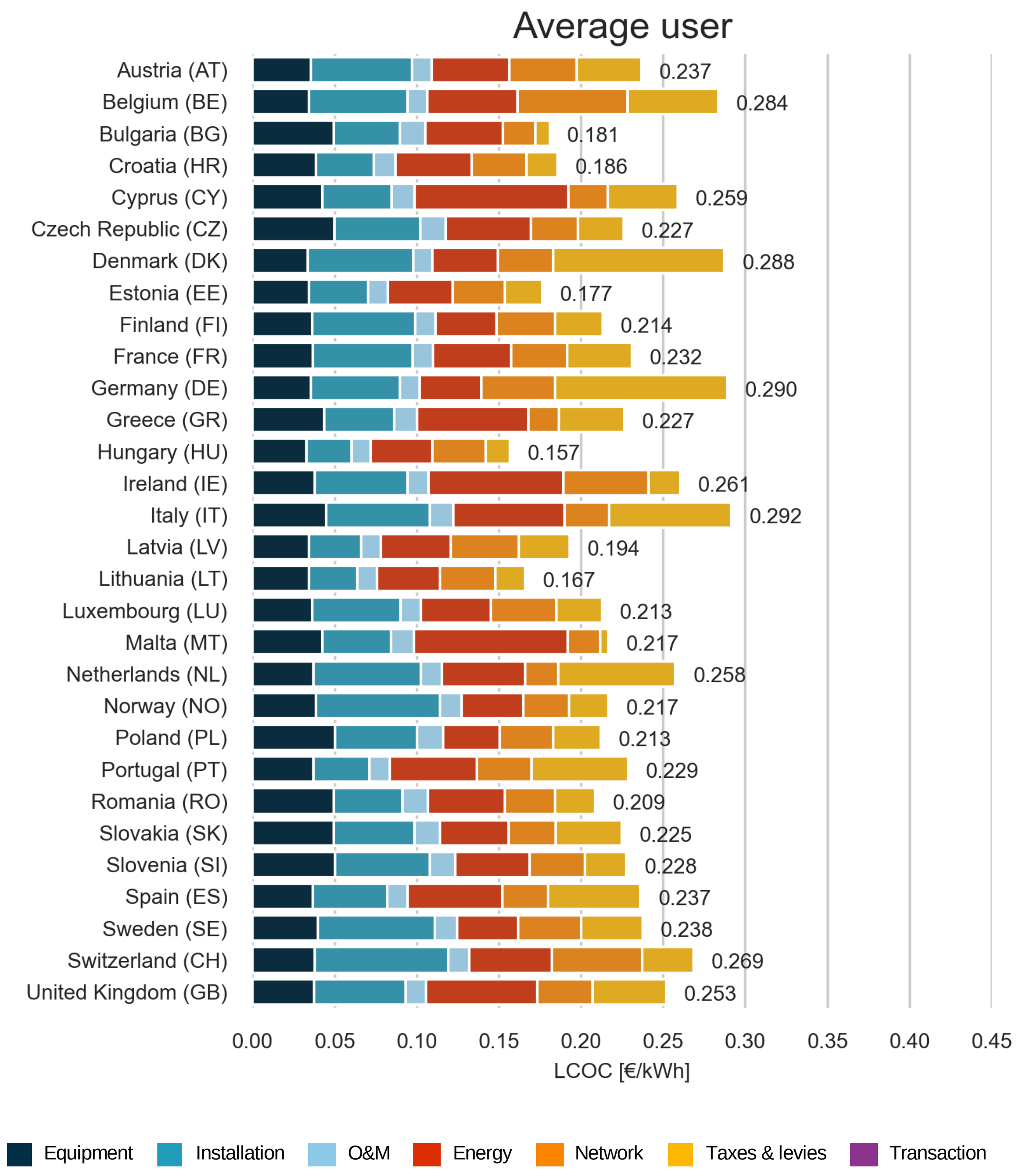

Supplementary Fig. 30. National LCOC in € per kWh of energy charged for user profile *Average user* by components for the *high* TOU tariff sensitivity (-30 % compared to average residential grid electricity prices). Each bar gives the total LCOC in € kWh^{-1} for a given country segmented into the main LCOC parameters (equipment, installation, O&M, energy, network, taxes & levies, transaction fees).

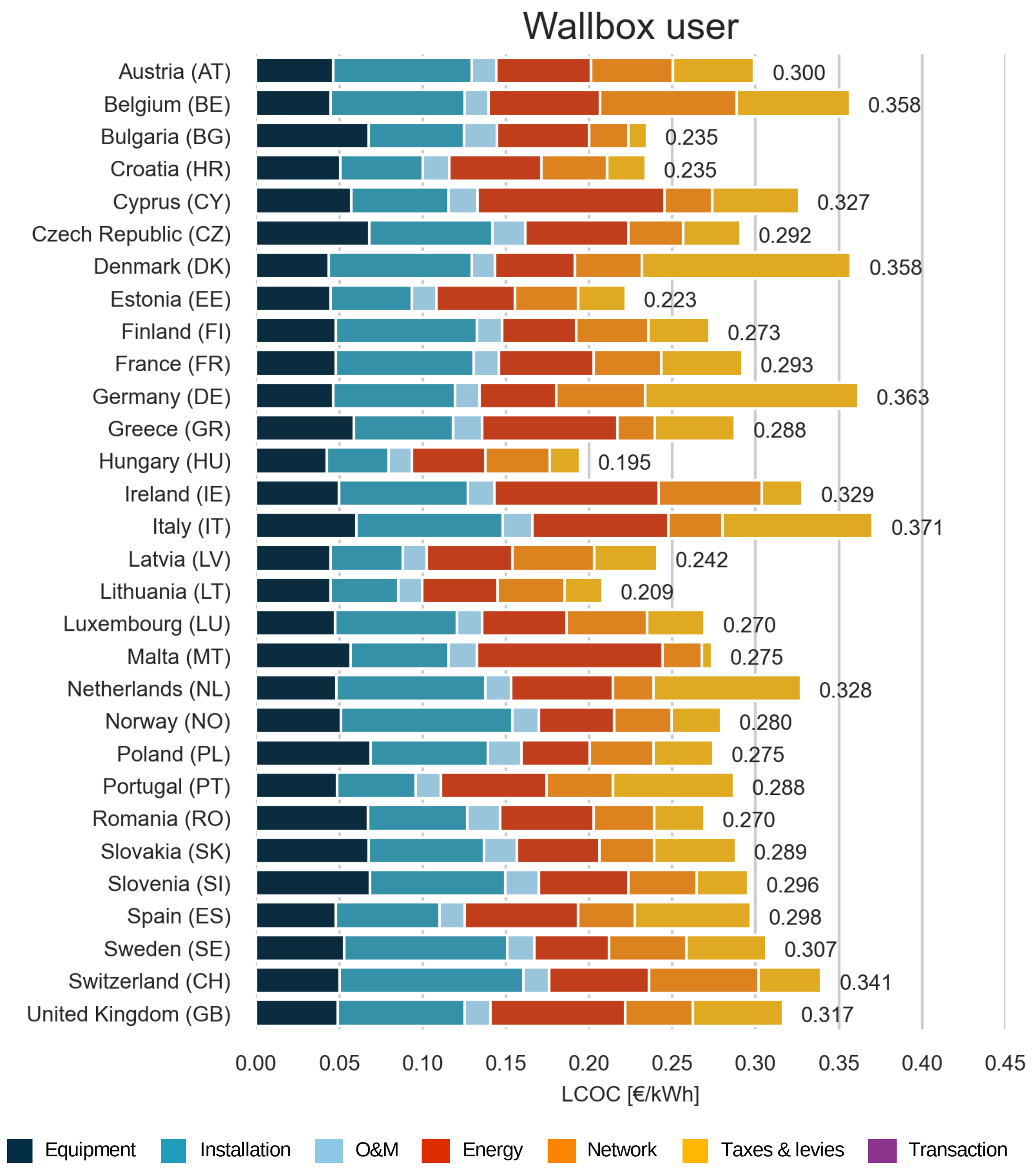

Supplementary Fig. 31. National LCOC in € per kWh of energy charged for user profile *Wallbox user* by components. Each bar gives the total LCOC in € kWh^{-1} for a given country segmented into the main LCOC parameters (equipment, installation, O&M, energy, network, taxes & levies, transaction fees).

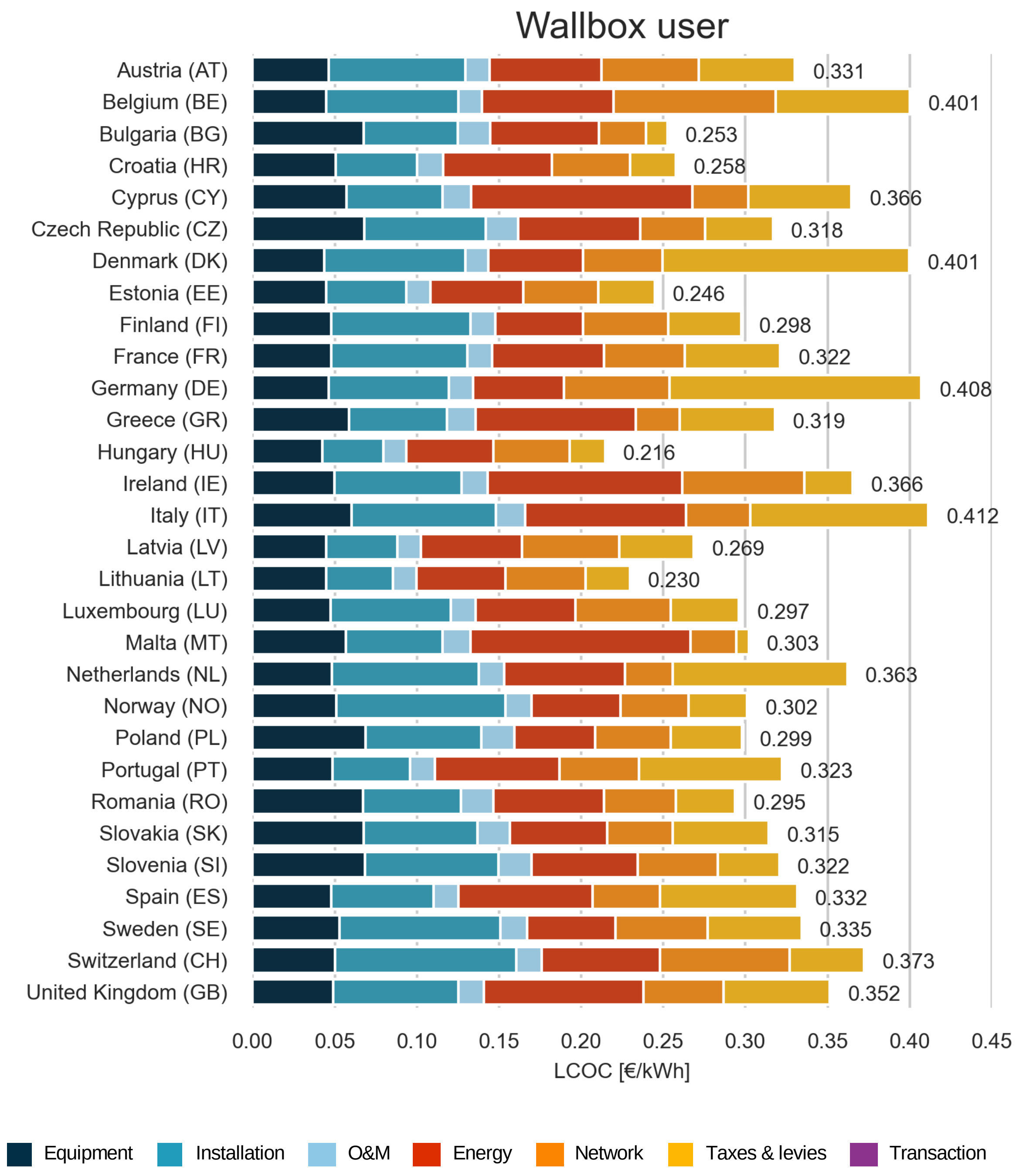

Supplementary Fig. 32. National LCOC in € per kWh of energy charged for user profile *Wallbox user* by components for the *high* electricity price sensitivity (+20 %). Each bar gives the total LCOC in € kWh^{-1} for a given country segmented into the main LCOC parameters (equipment, installation, O&M, energy, network, taxes & levies, transaction fees).

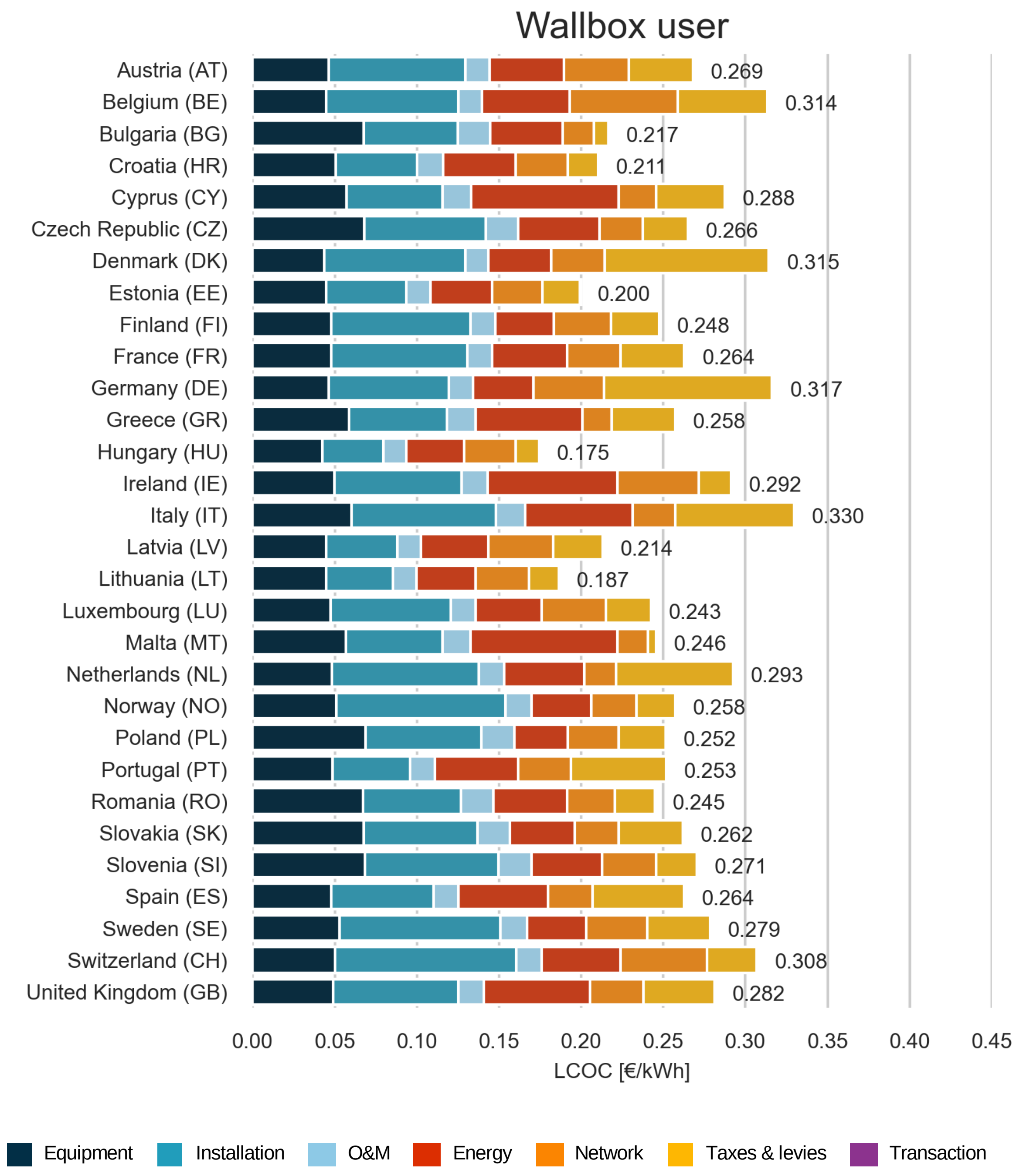

Supplementary Fig. 33. National LCOC in € per kWh of energy charged for user profile *Wallbox user* by components for the *low* electricity price sensitivity (-20 %). Each bar gives the total LCOC in € kWh^{-1} for a given country segmented into the main LCOC parameters (equipment, installation, O&M, energy, network, taxes & levies, transaction fees).

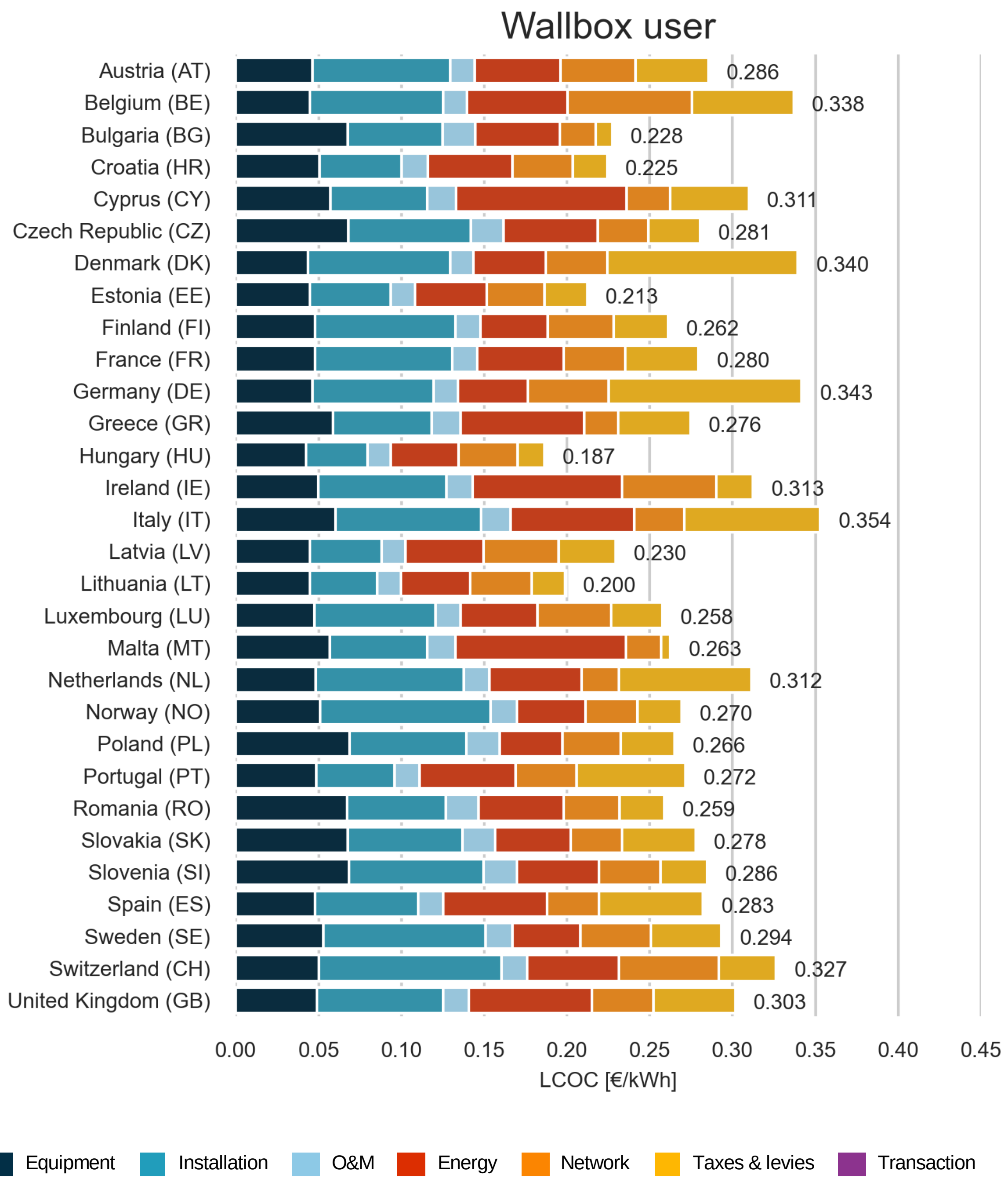

Supplementary Fig. 34. National LCOC in € per kWh of energy charged for user profile *Wallbox user* by components for the *medium* TOU tariff sensitivity (-20 % compared to average residential grid electricity prices). Each bar gives the total LCOC in € kWh^{-1} for a given country segmented into the main LCOC parameters (equipment, installation, O&M, energy, network, taxes & levies, transaction fees).

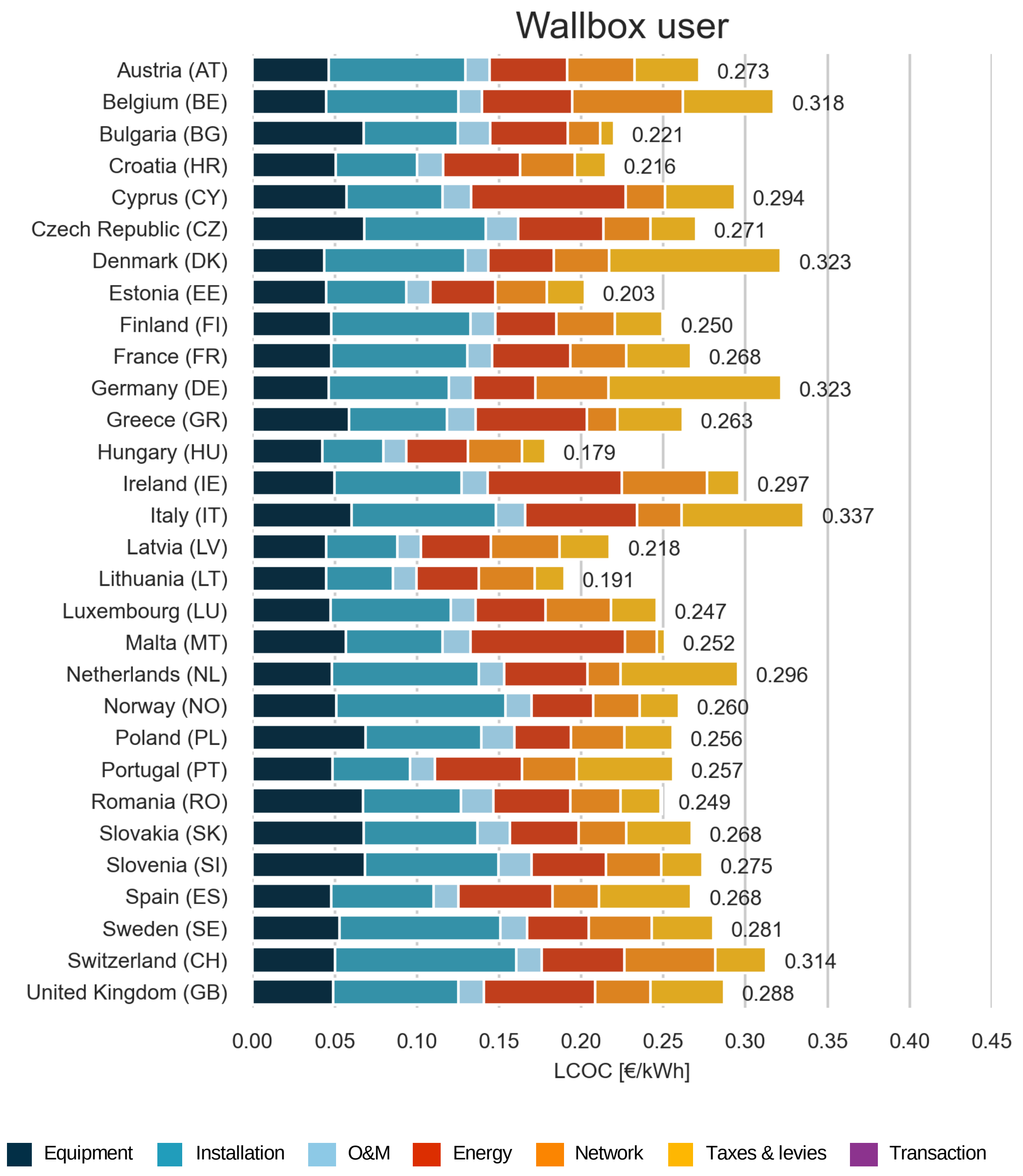

Supplementary Fig. 35. National LCOC in € per kWh of energy charged for user profile *Wallbox user* by components for the *high* TOU tariff sensitivity (-30 % compared to average residential grid electricity prices). Each bar gives the total LCOC in € kWh^{-1} for a given country segmented into the main LCOC parameters (equipment, installation, O&M, energy, network, taxes & levies, transaction fees).

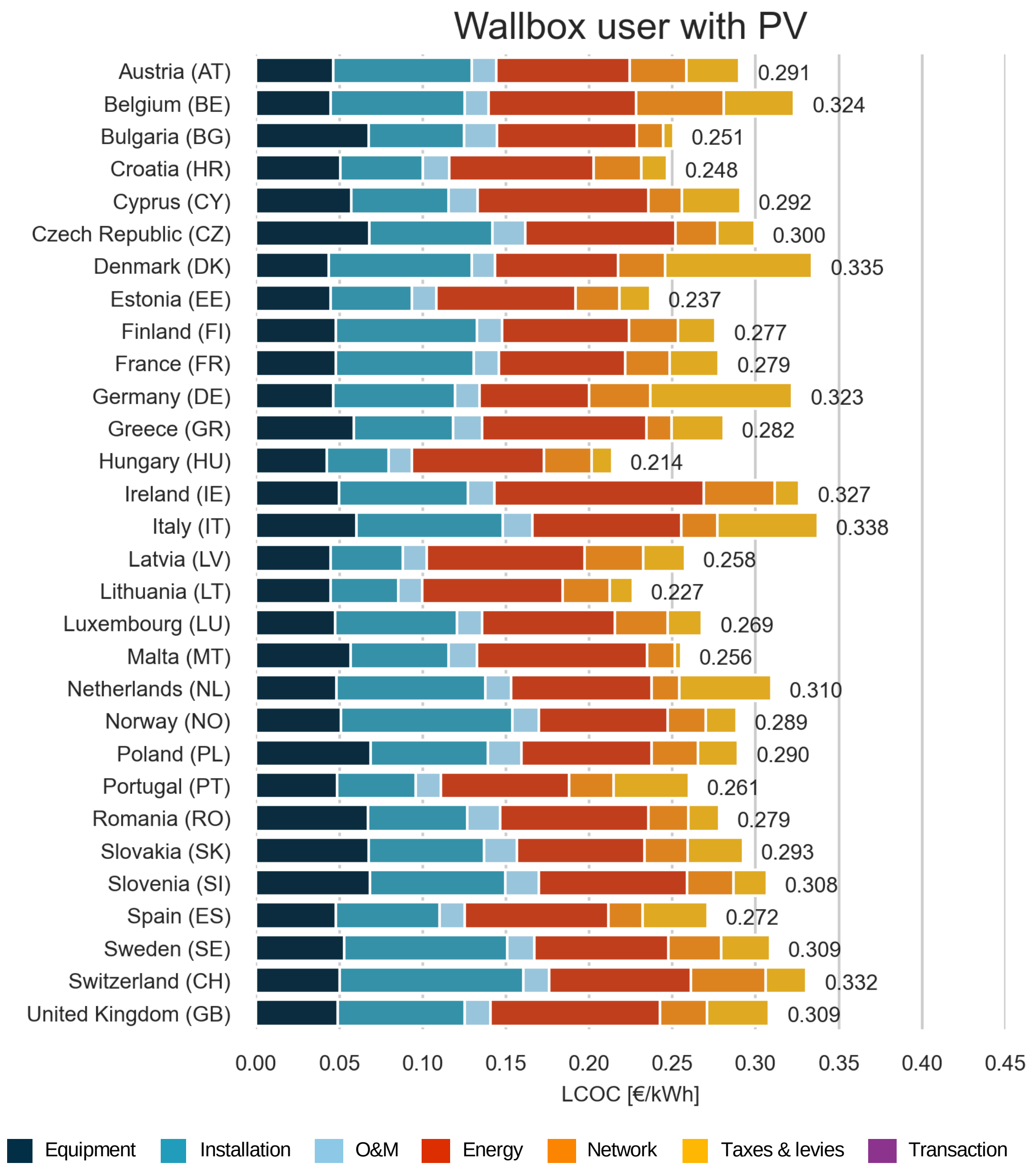

Supplementary Fig. 36. National LCOC in € per kWh of energy charged for user profile *Wallbox user with PV* by components. Each bar gives the total LCOC in € kWh^{-1} for a given country segmented into the main LCOC parameters (equipment, installation, O&M, energy, network, taxes & levies, transaction fees).

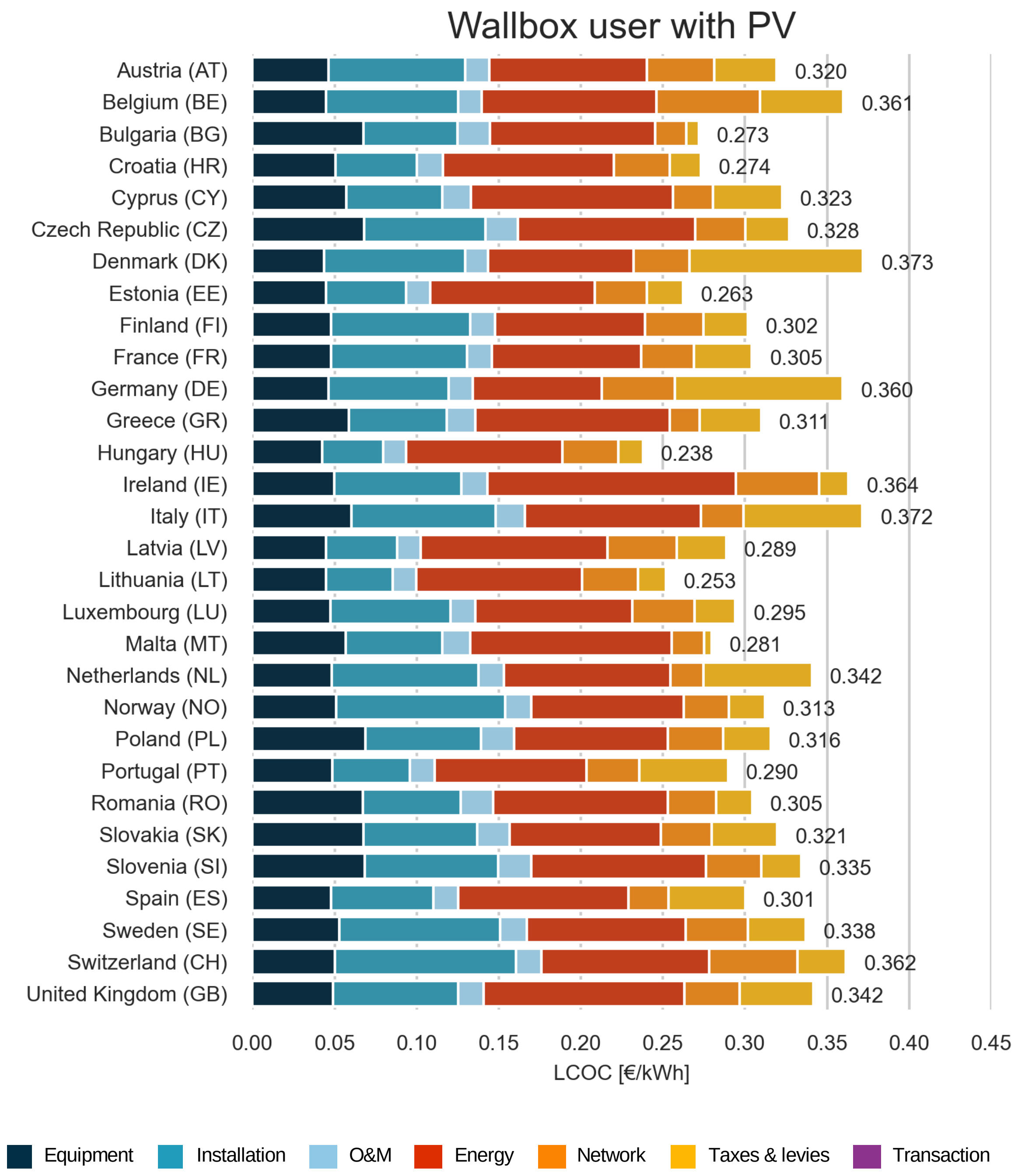

Supplementary Fig. 37. National LCOC in € per kWh of energy charged for user profile *Wallbox user with PV* by components for the *high* electricity price sensitivity (+20 %). Each bar gives the total LCOC in € kWh^{-1} for a given country segmented into the main LCOC parameters (equipment, installation, O&M, energy, network, taxes & levies, transaction fees).

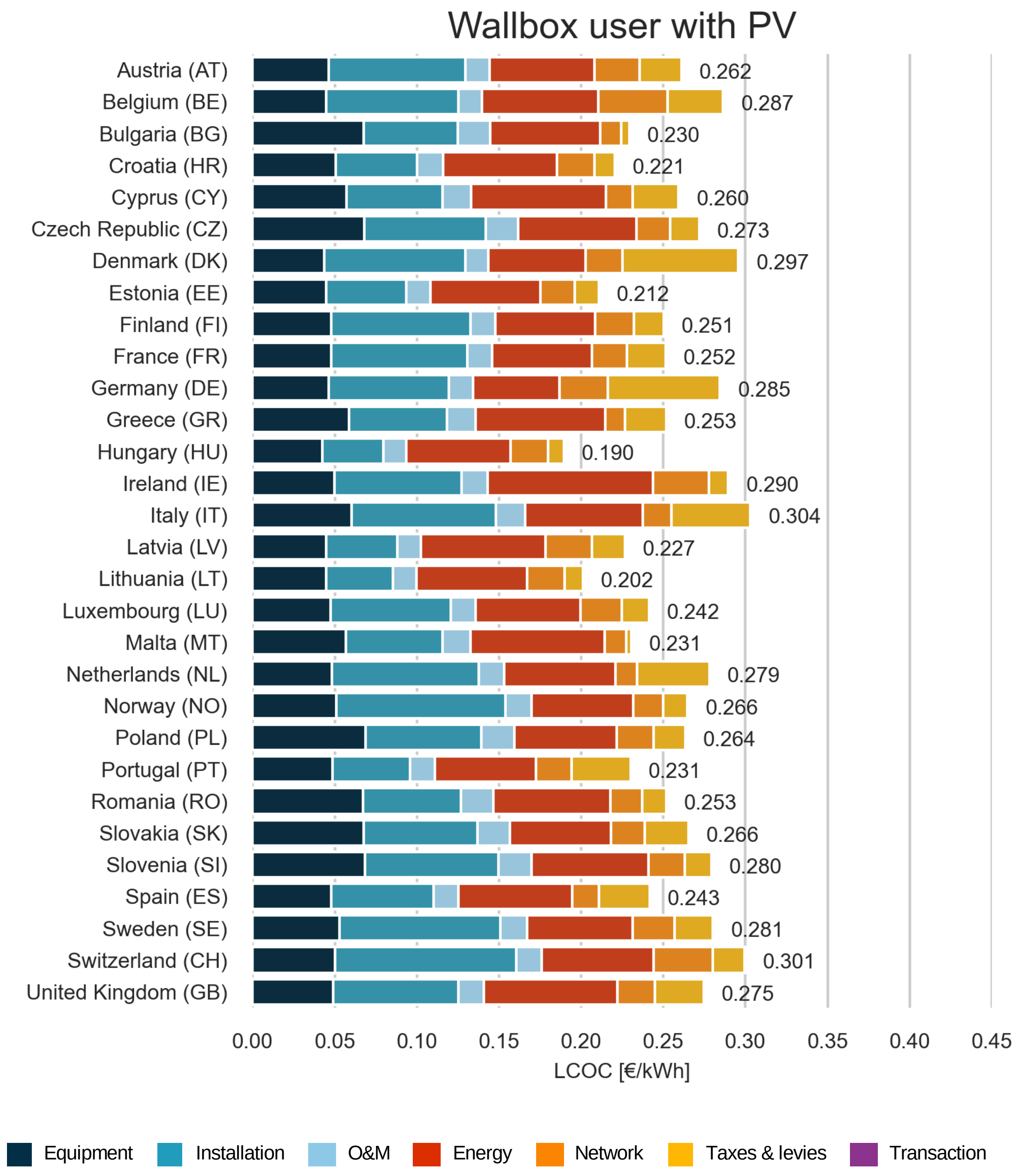

Supplementary Fig. 38. National LCOC in € per kWh of energy charged for user profile *Wallbox user with PV* by components for the *low* electricity price sensitivity (-20 %). Each bar gives the total LCOC in € kWh^{-1} for a given country segmented into the main LCOC parameters (equipment, installation, O&M, energy, network, taxes & levies, transaction fees).

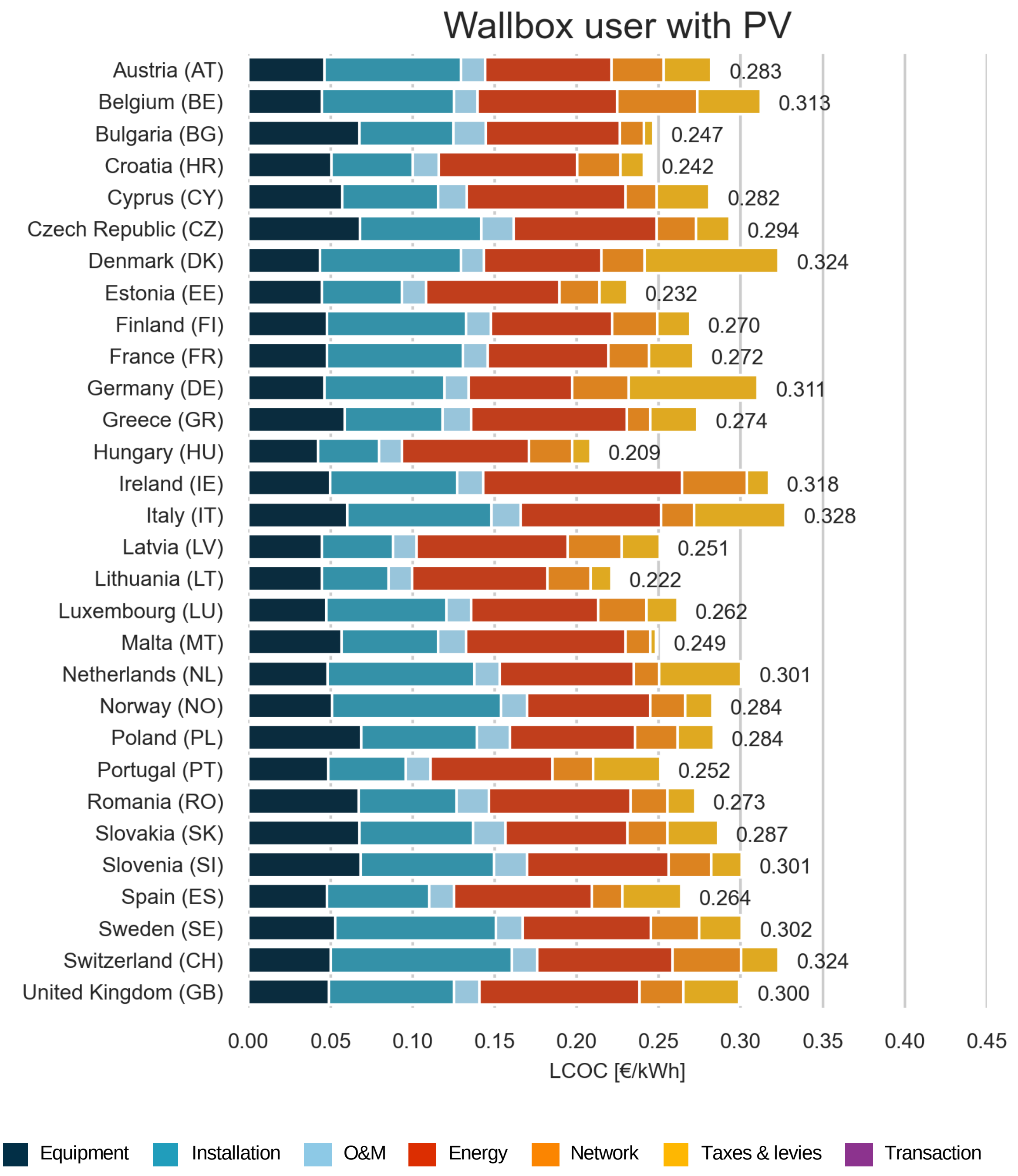

Supplementary Fig. 39. National LCOC in € per kWh of energy charged for user profile *Wallbox user with PV* by components for the *medium* TOU tariff sensitivity (-20 % compared to average residential grid electricity prices). Each bar gives the total LCOC in € kWh^{-1} for a given country segmented into the main LCOC parameters (equipment, installation, O&M, energy, network, taxes & levies, transaction fees).

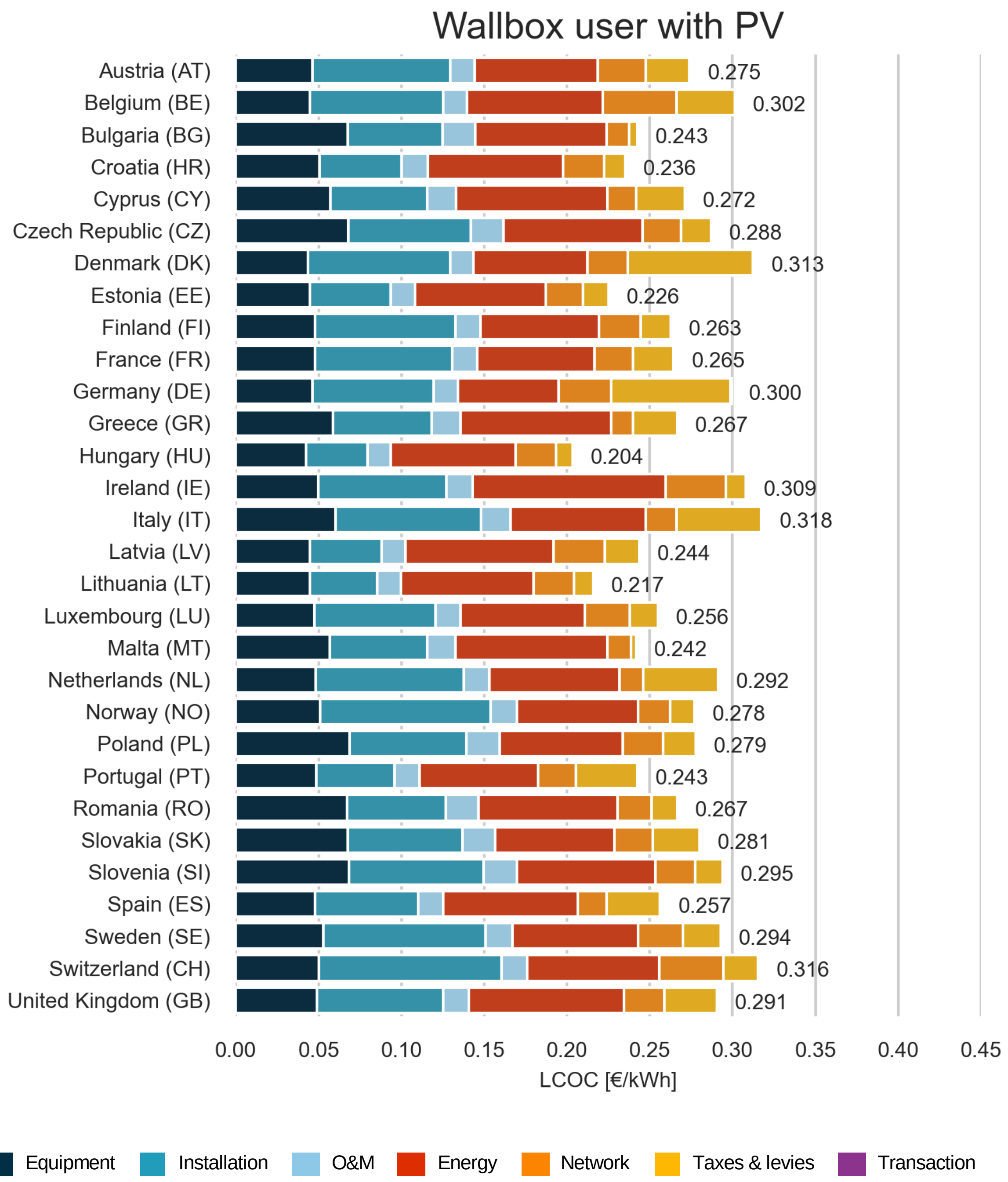

Supplementary Fig. 40. National LCOC in € per kWh of energy charged for user profile *Wallbox user with PV* by components for the *high* TOU tariff sensitivity (-30 % compared to average residential grid electricity prices). Each bar gives the total LCOC in € kWh^{-1} for a given country segmented into the main LCOC parameters (equipment, installation, O&M, energy, network, taxes & levies, transaction fees).

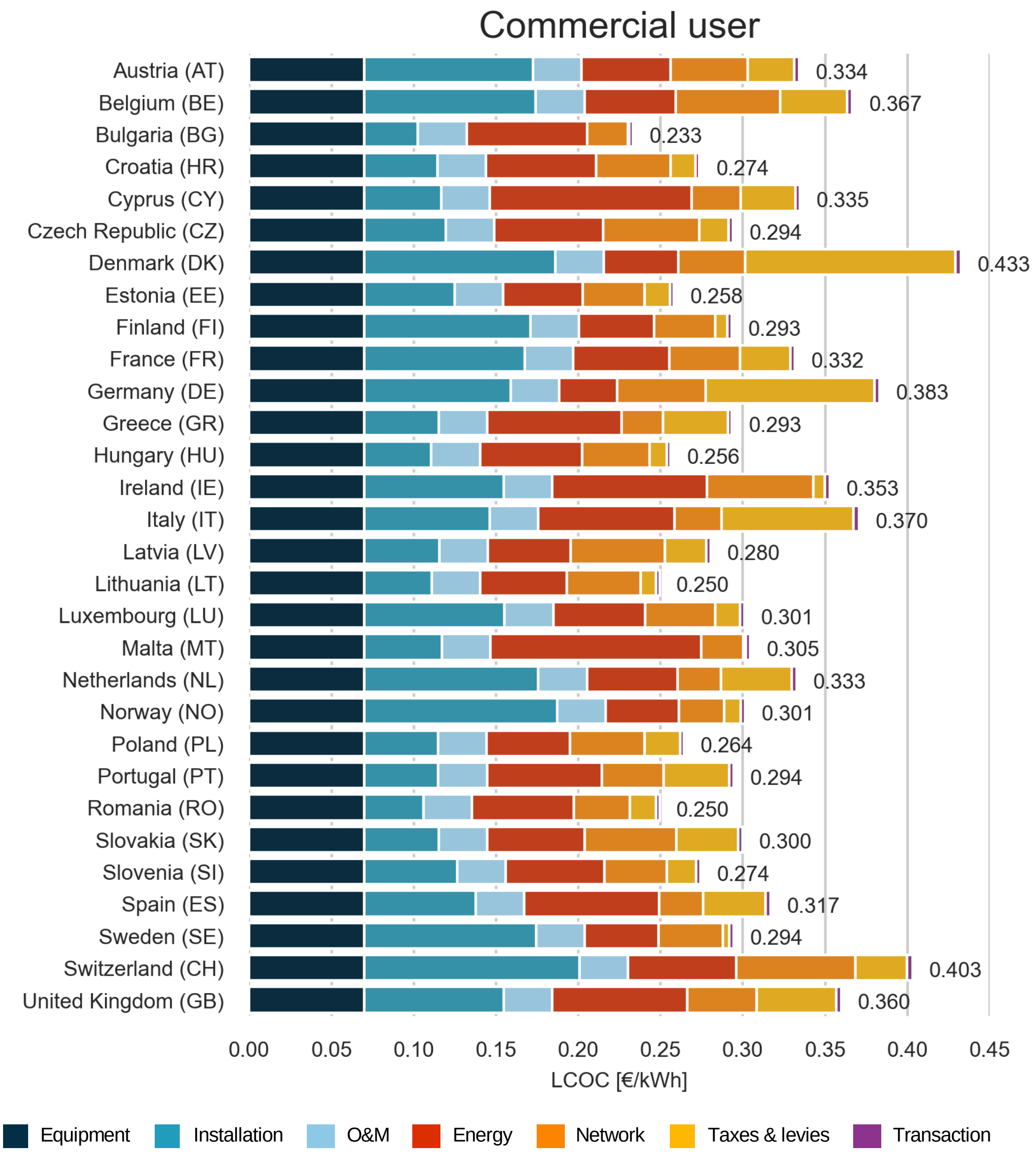

Supplementary Fig. 41. National LCOC in € per kWh of energy charged for user profile *Commercial user* by components. Each bar gives the total LCOC in € kWh^{-1} for a given country segmented into the main LCOC parameters (equipment, installation, O&M, energy, network, taxes & levies, transaction fees).

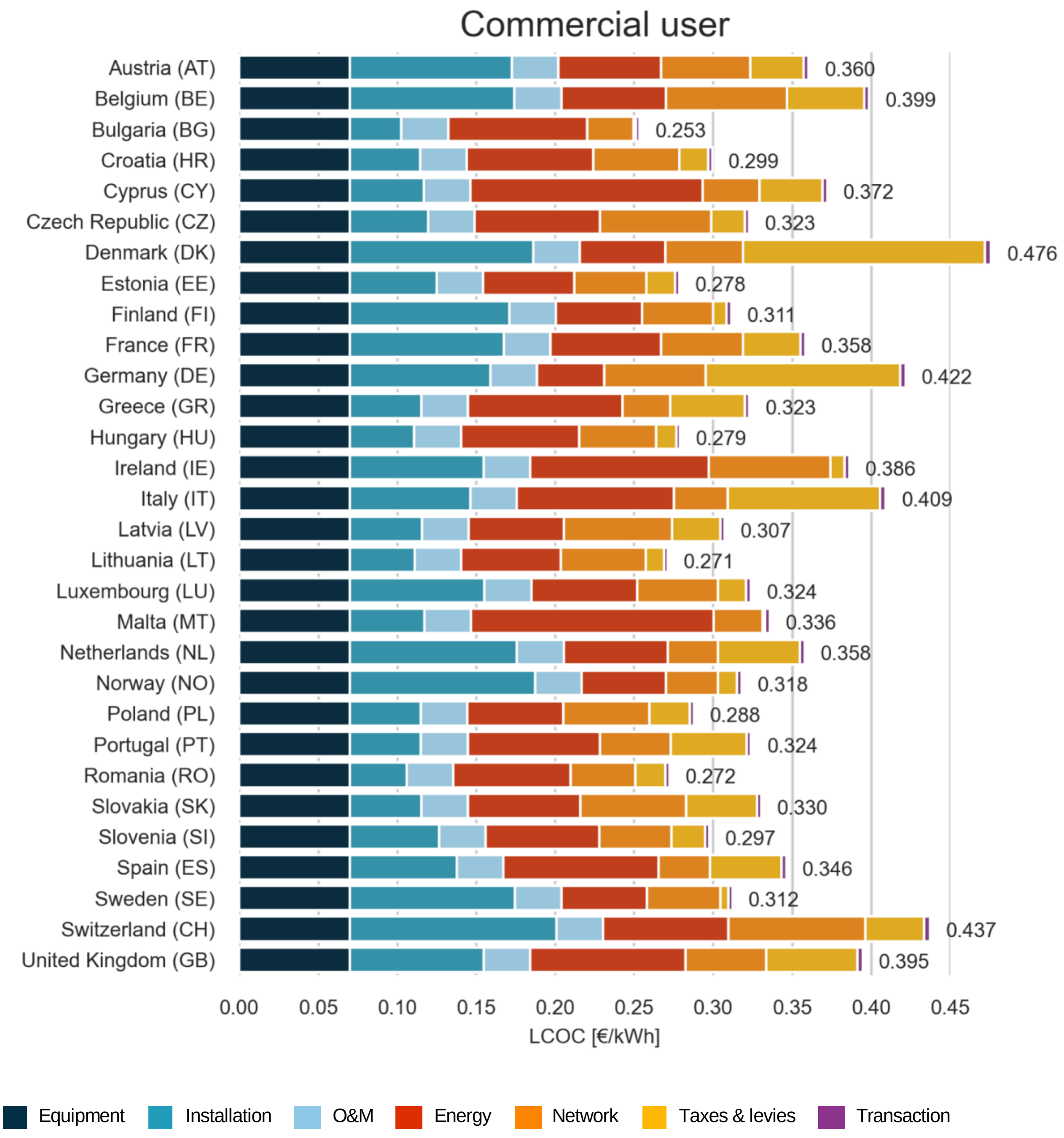

Supplementary Fig. 42. National LCOC in € per kWh of energy charged for user profile *Commercial user* by components for the *high* electricity price sensitivity (+20 %). Each bar gives the total LCOC in € kWh^{-1} for a given country segmented into the main LCOC parameters (equipment, installation, O&M, energy, network, taxes & levies, transaction fees).

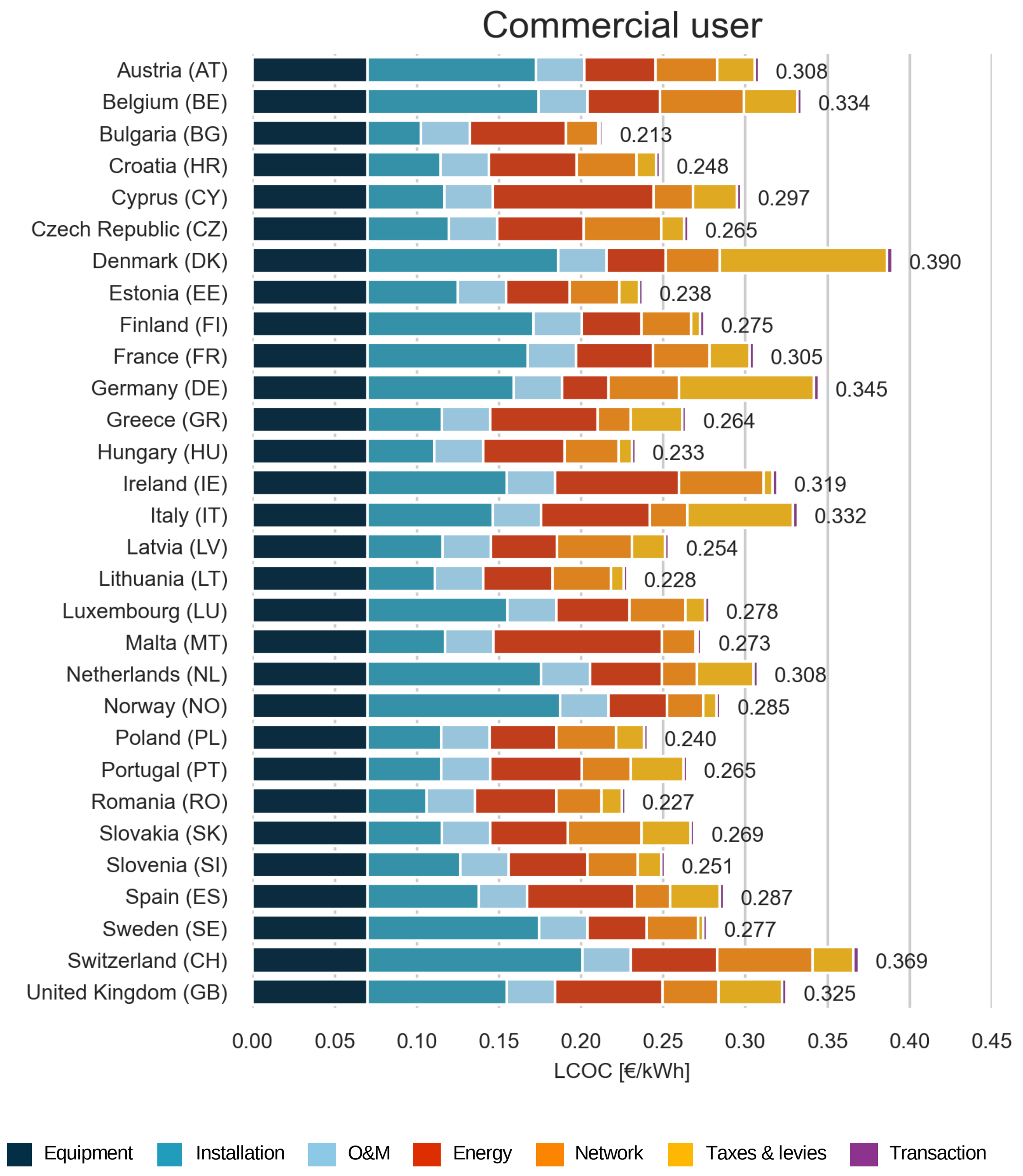

Supplementary Fig. 43. National LCOC in € per kWh of energy charged for user profile *Commercial user* by components for the *low* electricity price sensitivity (-20 %). Each bar gives the total LCOC in € kWh^{-1} for a given country segmented into the main LCOC parameters (equipment, installation, O&M, energy, network, taxes & levies, transaction fees).

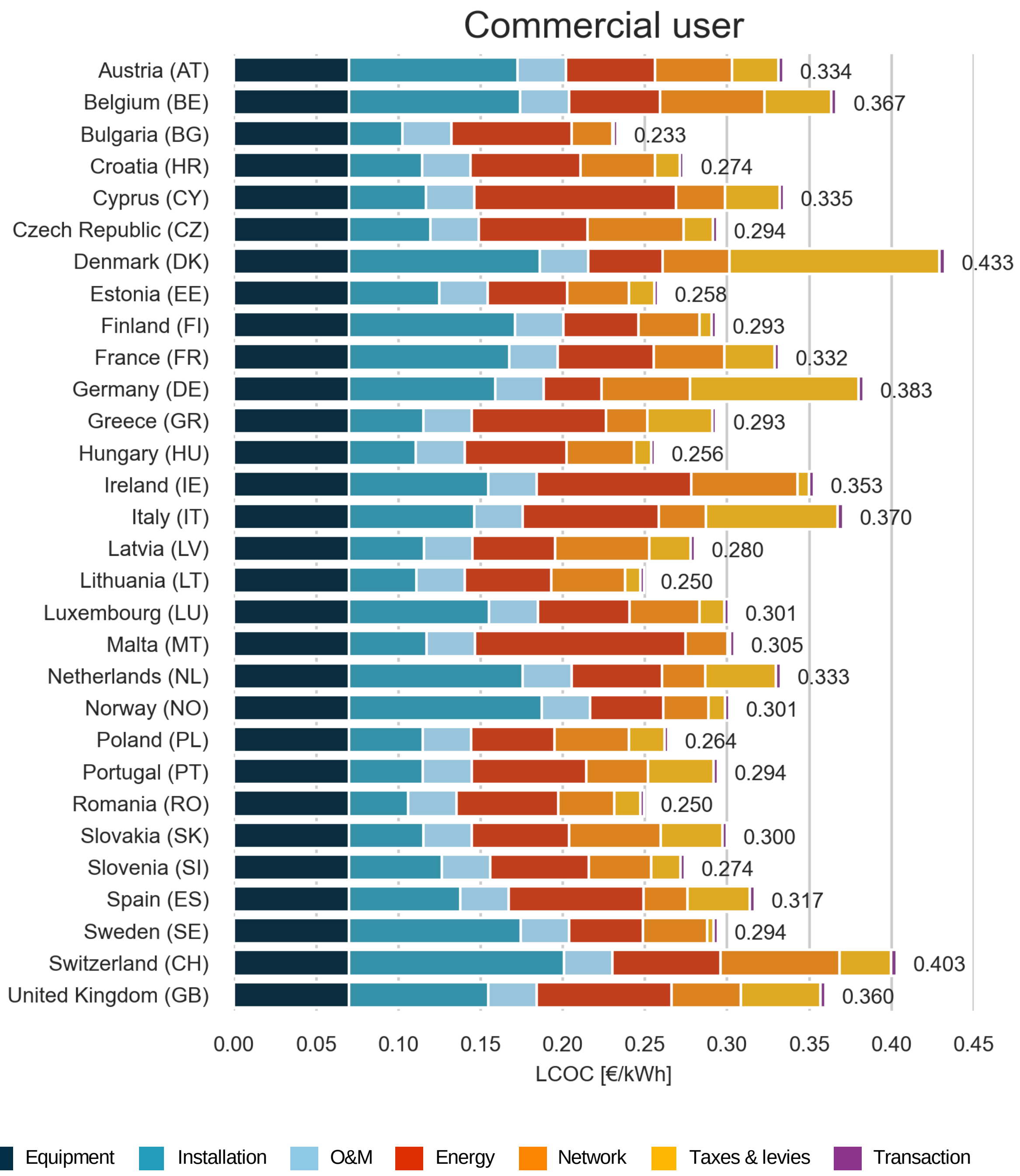

Supplementary Fig. 44. National LCOC in € per kWh of energy charged for user profile *Commercial user* by components for the *medium* TOU tariff sensitivity (-20 % compared to average residential grid electricity prices). Each bar gives the total LCOC in € kWh^{-1} for a given country segmented into the main LCOC parameters (equipment, installation, O&M, energy, network, taxes & levies, transaction fees).

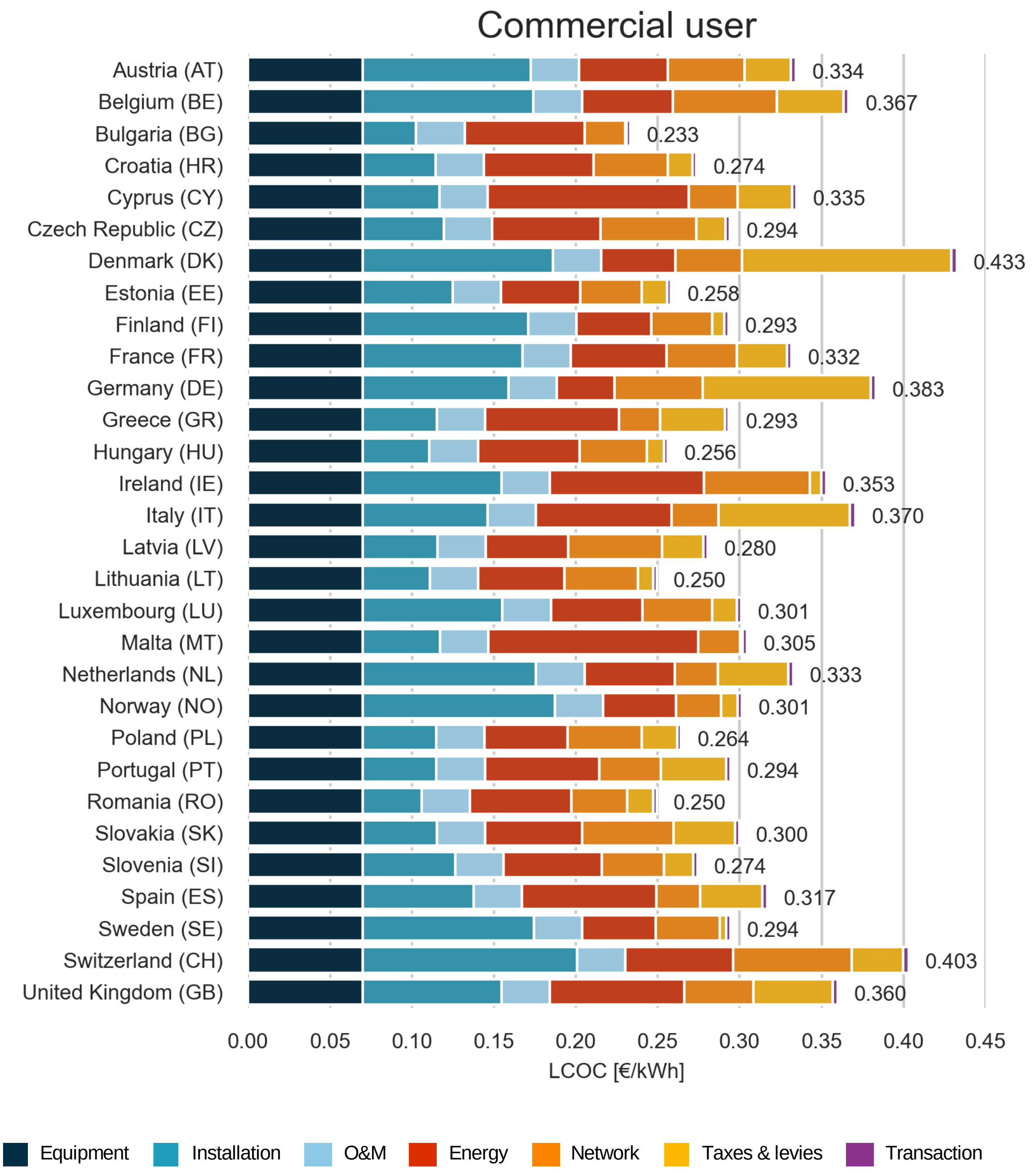

Supplementary Fig. 45. National LCOC in € per kWh of energy charged for user profile *Commercial user* by components for the *high* TOU tariff sensitivity (-30 % compared to average residential grid electricity prices). Each bar gives the total LCOC in € kWh^{-1} for a given country segmented into the main LCOC parameters (equipment, installation, O&M, energy, network, taxes & levies, transaction fees).

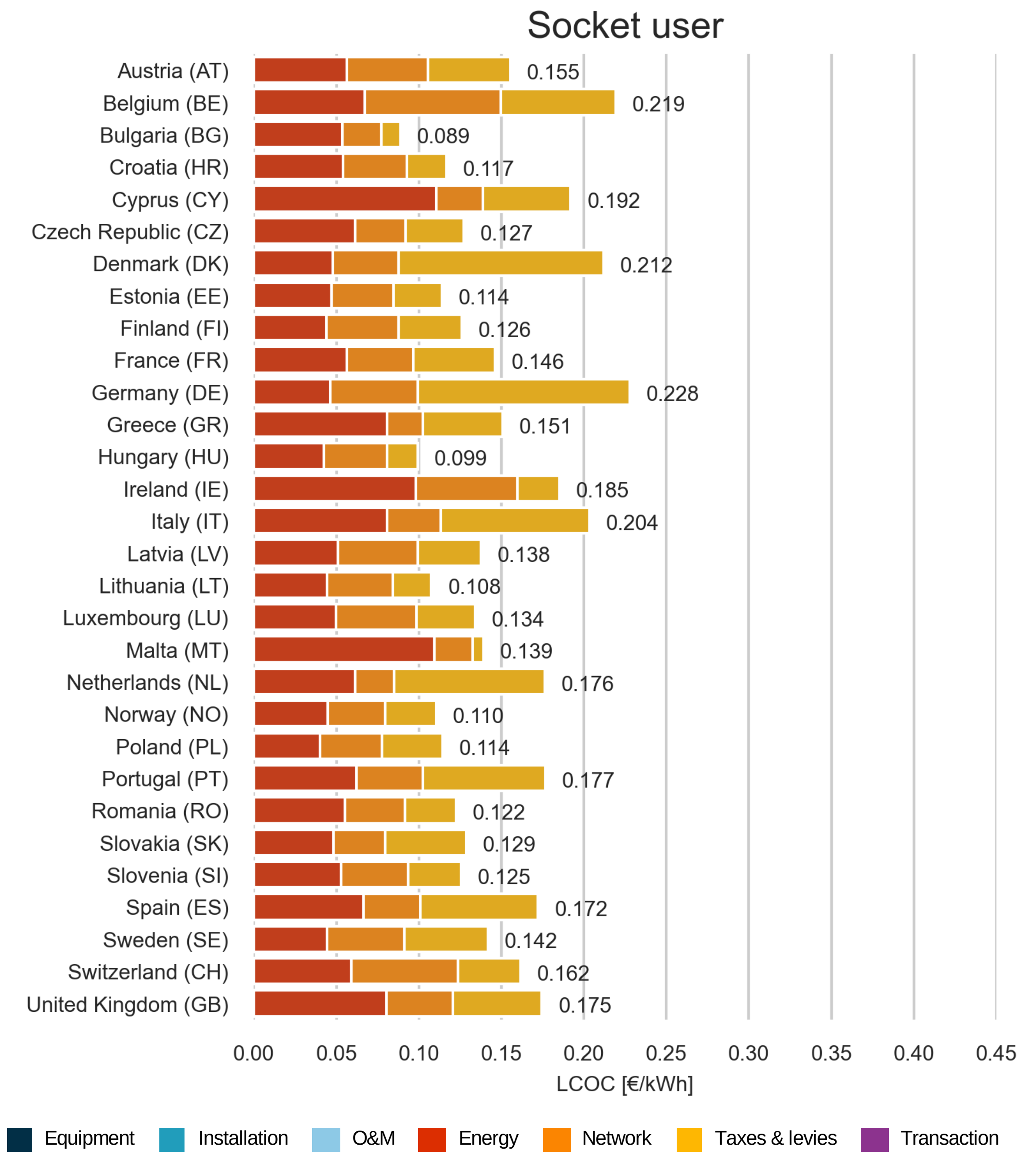

Supplementary Fig. 46. National LCOC in € per kWh of energy charged for user profile *Socket user* by components. Each bar gives the total LCOC in € kWh^{-1} for a given country segmented into the main LCOC parameters (equipment, installation, O&M, energy, network, taxes & levies, transaction fees).

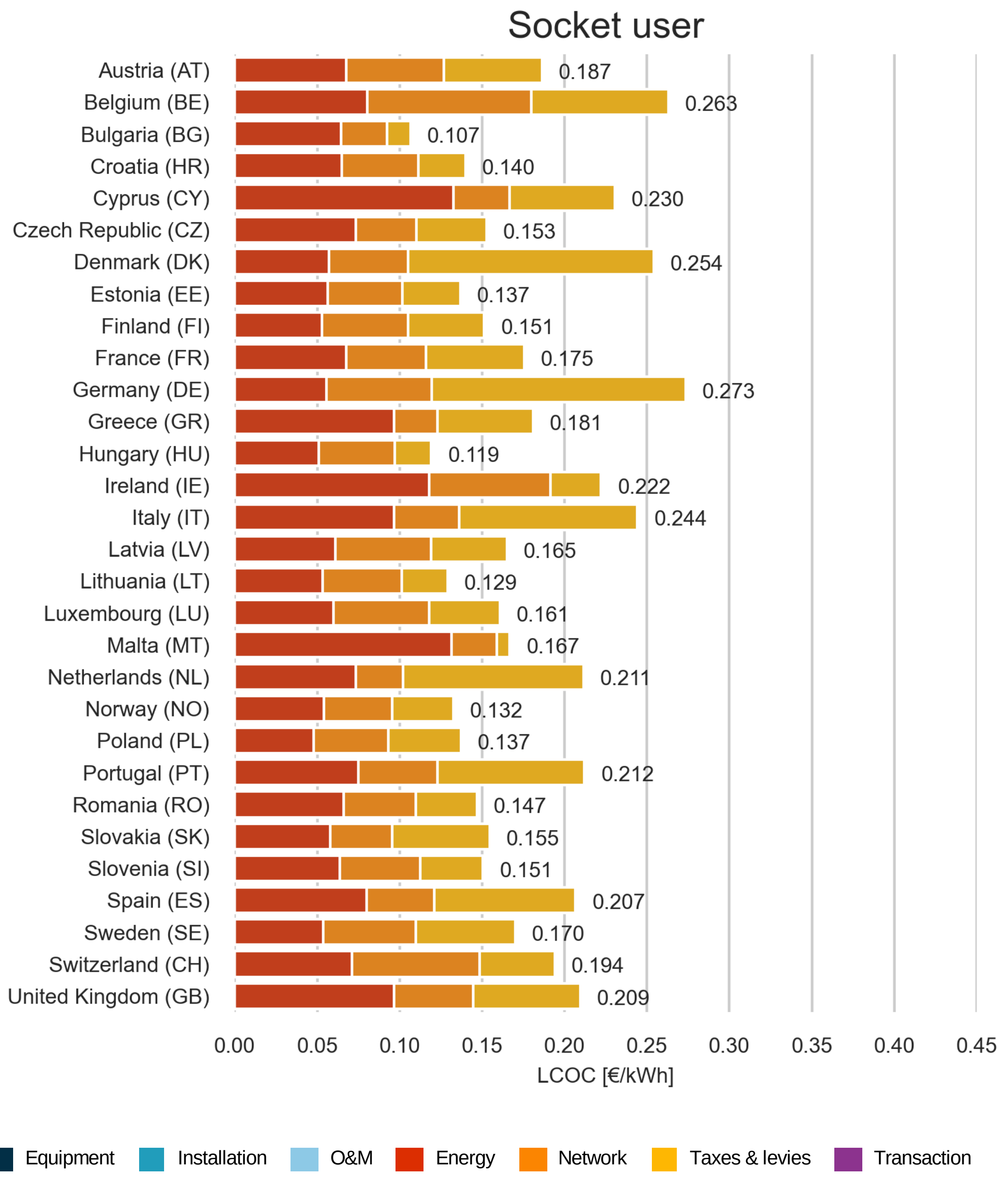

Supplementary Fig. 47. National LCOC in € per kWh of energy charged for user profile *Socket user* by components for the *high* electricity price sensitivity (+20 %). Each bar gives the total LCOC in € kWh^{-1} for a given country segmented into the main LCOC parameters (equipment, installation, O&M, energy, network, taxes & levies, transaction fees).

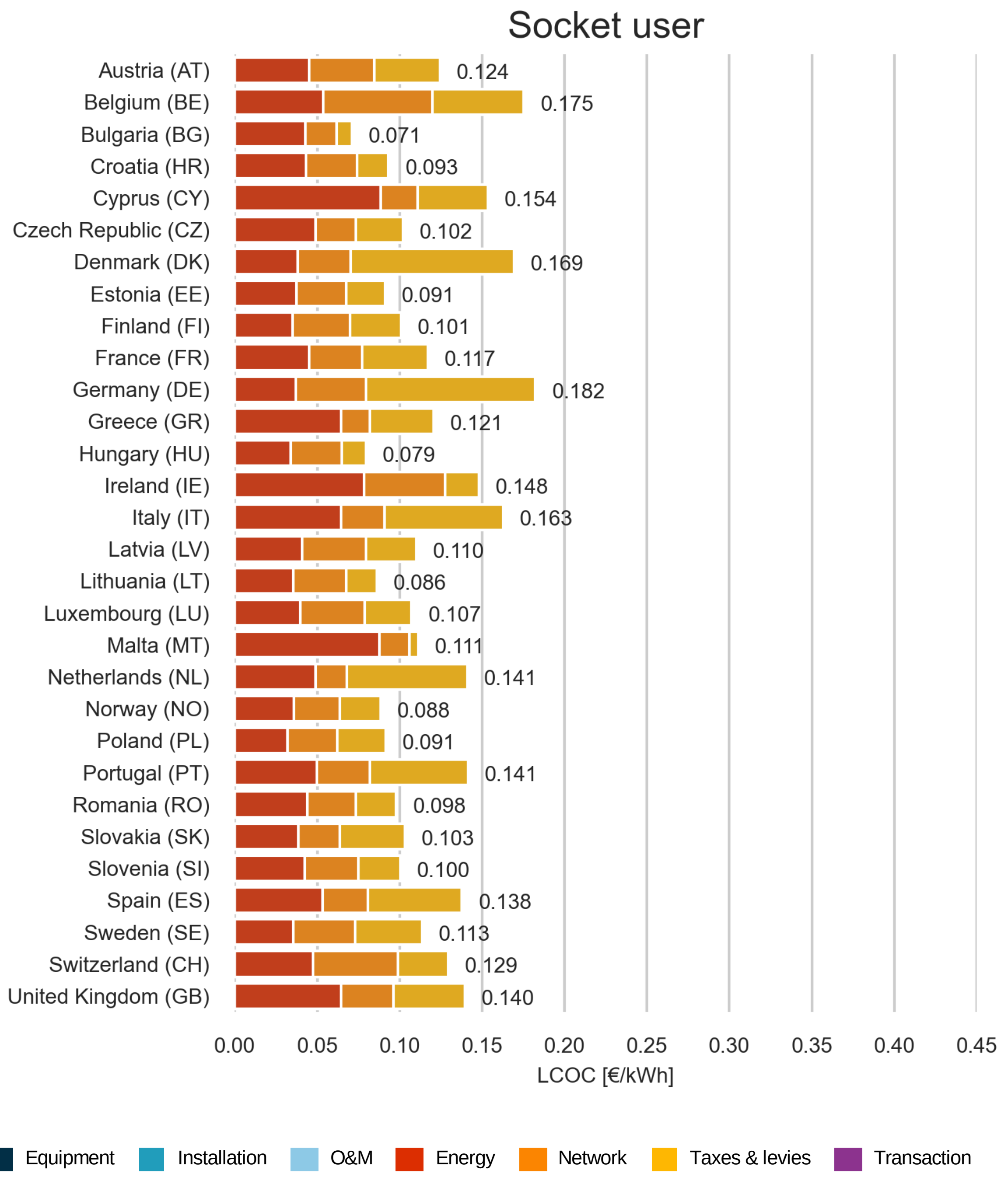

Supplementary Fig. 48. National LCOC in € per kWh of energy charged for user profile *Socket user* by components for the *low* electricity price sensitivity (-20 %). Each bar gives the total LCOC in € kWh^{-1} for a given country segmented into the main LCOC parameters (equipment, installation, O&M, energy, network, taxes & levies, transaction fees).

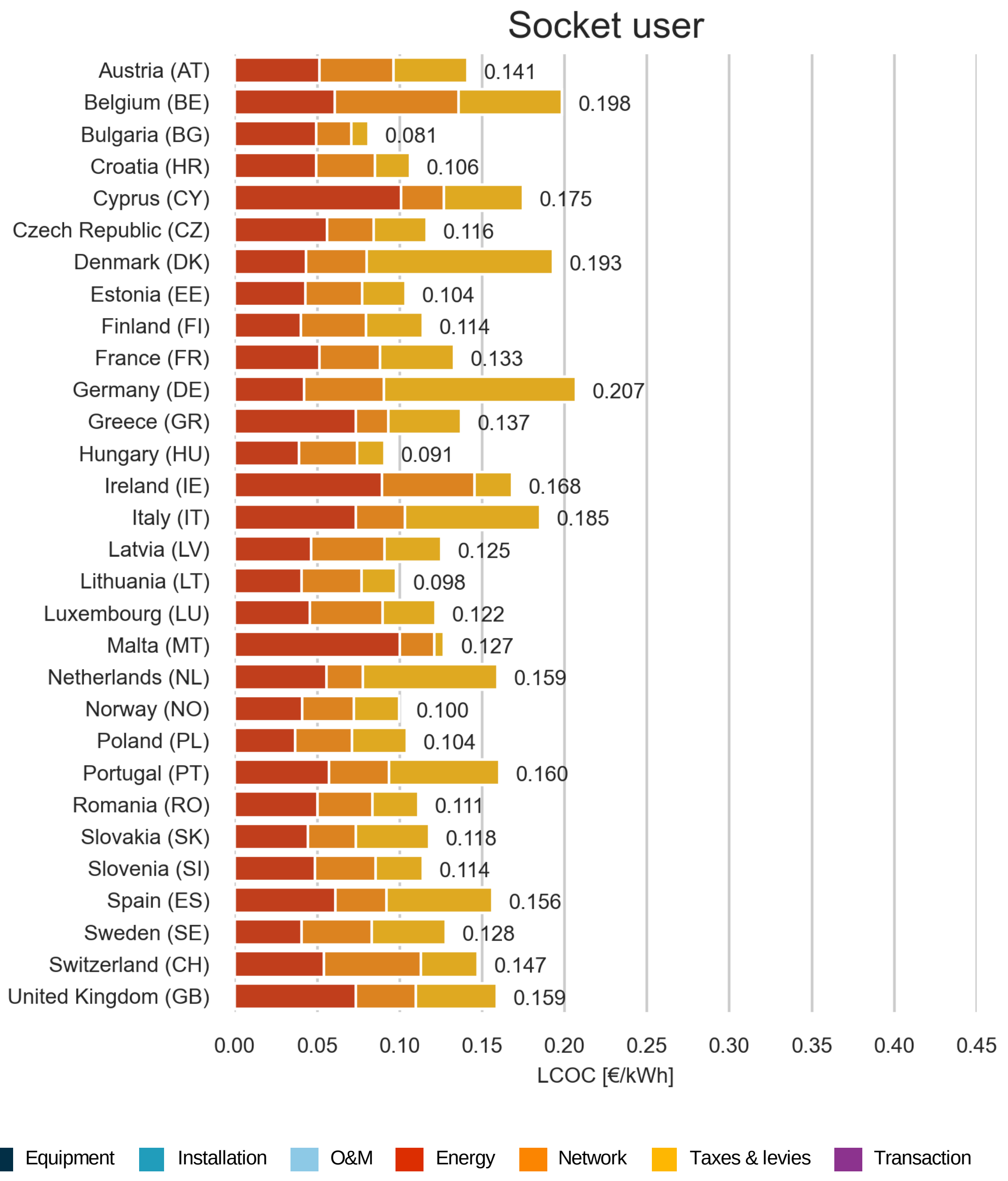

Supplementary Fig. 49. National LCOC in € per kWh of energy charged for user profile *Socket user* by components for the *medium* TOU tariff sensitivity (-20 % compared to average residential grid electricity prices). Each bar gives the total LCOC in € kWh^{-1} for a given country segmented into the main LCOC parameters (equipment, installation, O&M, energy, network, taxes & levies, transaction fees).

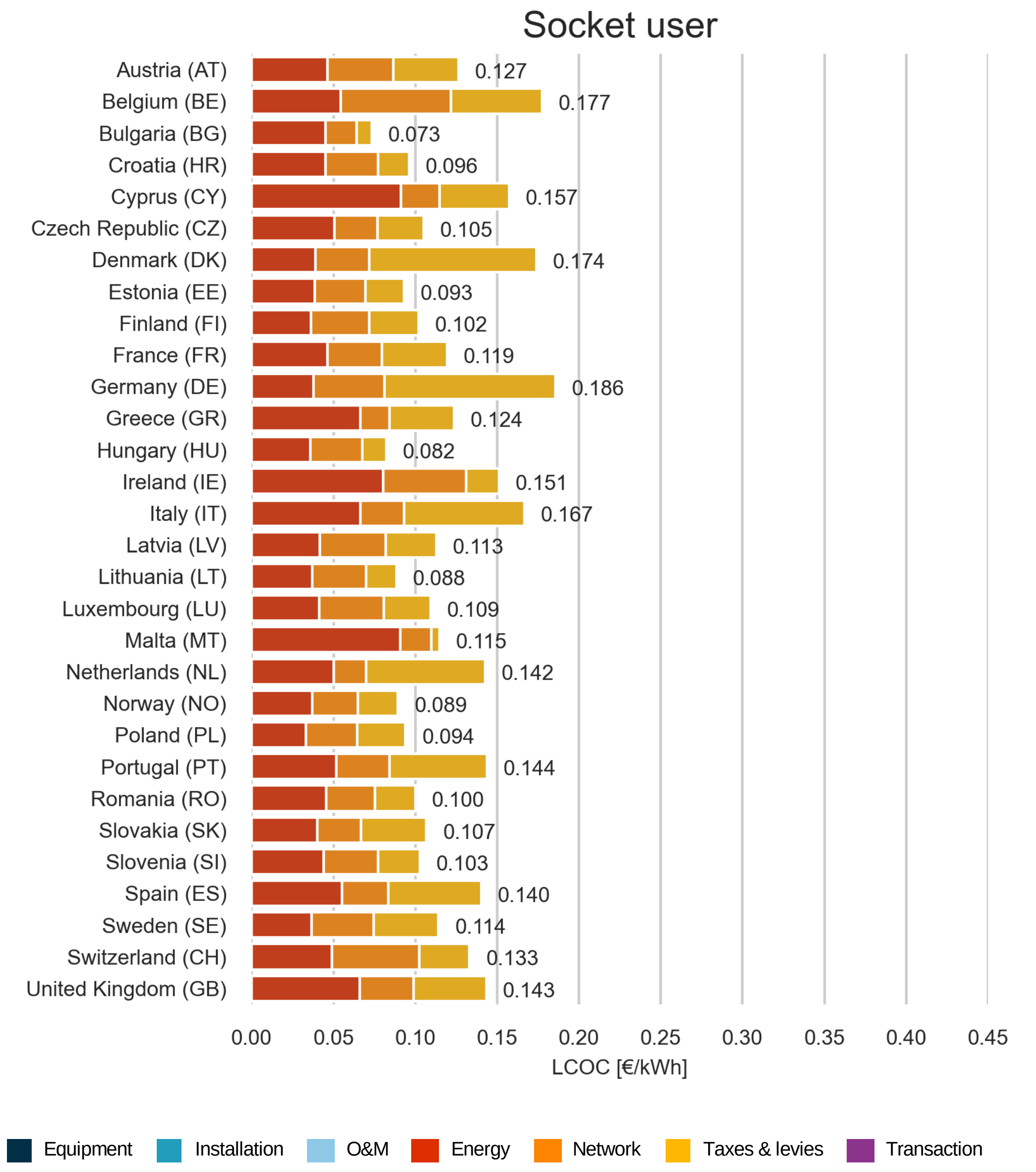

Supplementary Fig. 50. National LCOC in € per kWh of energy charged for user profile *Socket user* by components for the *high* TOU tariff sensitivity (-30 % compared to average residential grid electricity prices). Each bar gives the total LCOC in € kWh^{-1} for a given country segmented into the main LCOC parameters (equipment, installation, O&M, energy, network, taxes & levies, transaction fees).

SUPPLEMENTARY REFERENCES

1. Bódis, K., Kougias, I., Jäger-Waldau, A., Taylor, N. & Szabó, S. A high-resolution geospatial assessment of the rooftop solar photovoltaic potential in the European Union. *Renew. Sustain. Energy Rev.* **114**, (2019).

2. Element Energy. *Electric Vehicle Charging Behaviour Study - Final report for National Grid ESO*. (2019).

3. Zhang, J., Yan, J., Liu, Y., Zhang, H. & Lv, G. Daily electric vehicle charging load profiles considering demographics of vehicle users. *Appl. Energy* **274**, (2020).

4. Heinz, D. Erstellung und Auswertung repräsentativer Mobilitäts- und Ladeprofile für Elektrofahrzeuge in Deutschland. *Work. Pap. Ser. Prod. Energy 30* (2018).

5. Noussan, M. & Neirotti, F. Cross-country comparison of hourly electricity mixes for EV charging profiles. *Energies* **13**, 2527 (2020).

6. Pfenninger, S. & Staffell, I. Long-term patterns of European PV output using 30 years of validated hourly reanalysis and satellite data. *Energy* **114**, 1251–1265 (2016).

7. European Commission. Standard time. https://ec.europa.eu/transport/themes/summertime_en (2021).

8. Bauer, C., Cox, B., Heck, T. & Zhang, X. *Potentials, costs and environmental assessment of electricity generation technologies - An update of electricity generation costs and potentials*. (2019).

9. Nelder, C. & Rogers, E. *Reducing EV Charging Infrastructure Costs*. (2019).

10. Morgan, D. Do electric cars come with a charging cable? https://www.smarthomecharge.co.uk/guides/what-cables-come-with-an-electric-car/ (2019).

11. Borlaug, B., Salisbury, S., Gerdes, M. & Muratori, M. Levelized Cost of Charging Electric Vehicles in the United States. *Joule* **4**, 1–16 (2020).

12. Lee, H. & Clark, A. Charging the Future: Challenges and Opportunities for Electric Vehicle Adoption. *SSRN Electron. J.* (2018) doi:10.2139/ssrn.3251551.

13. Cregger, J. Charging Infrastructure Required to Support U.S. Electric Vehicle Deployment: A Cost Estimate through 2025. in *2015 IEEE Vehicle Power and Propulsion Conference, VPPC 2015 - Proceedings* (2015). doi:10.1109/VPPC.2015.7352909.

14. Electric Power Research Institute (EPRI). *Electric Vehicle Supply Equipment Installed Cost Analysis*. *3002000577* (2013).

15. The Mobility House. Installationskosten für Ladestationen - Konkrete Anwendungsbeispiele. (2021).

16. Novavolt. Grobkosten für Ihre Ladeinfrastruktur - Ready2Charge. https://novavolt.ch/ready2charge-rechner/ (2020).

17. Sun, L., Lubkeman, D. & Baran, M. Levelized Cost Analysis of Medium Voltage DC Fast Charging

Station. in *IEEE Power and Energy Society General Meeting* vols 2019-August (2019).

18. Nicholas, M. & Hall, D. *Lessons learnes on early electric vehicle fast-charging deployments*. *ICCT White Paper* (2018).

19. European Central Bank (ECB); national central banks (NCBs) of the Eurosystem. Euro area statistics. (2021).

20. Drupp, M. A., Freeman, M. C., Groom, B. & Nesje, F. Discounting disentangled. *Am. Econ. J. Econ. Policy* **10**, 109–134 (2018).

21. Norris, J. & Escher, G. *Heavy Duty Vehicles Technology Potential and Cost Study*. (2017).

22. Feng, W. & Figliozzi, M. A. Conventional vs Electric Commercial Vehicle Fleets: A Case Study of Economic and Technological Factors Affecting the Competitiveness of Electric Commercial Vehicles in the USA. *Procedia - Soc. Behav. Sci.* **39**, 702–711 (2012).

23. Cambridge Econometrics. *Trucking into a Greener Future: the economic impact of decarbonizing goods vehicles in Europe*. (2018).

24. Egli, F., Steffen, B. & Schmidt, T. S. Bias in energy system models with uniform cost of capital assumption. *Nat. Commun.* **10**, 4588 (2019).

25. Ortner, A., Welisch, M., Busch, S. & Resch, G. *Policy Dialogue on the assessment and convergence of RES Policy in EU Member States*. (2016).

26. Francfort, J. *Efficiency and Security Testing of EVSE, DC Fast Chargers, and Wireless Charging Systems*. (2013).

27. De Keulenaer, H., Chapman, D. & Fassbinder, S. The scope for energy saving in the EU through the use of energy-efficient electricity distribution transformers. in *IEE Conference Publication* vol. 4 (2001).

28. ABB. Terra 54 multi-standard DC charging station - Product Leaflet. https://library.e.abb.com/public/9f72c044f8ab4ff29c5ae0eb915c0843/4EVC901707-LFEN_Terra 54_11_19.pdf (2019).

29. ISO. Online Browsing Platform (OBP). https://www.iso.org/obp/ui/#search (2021).

30. Eurostat. *Population on 1 January 2019*. (2019).

31. Norwegian Tax Administration. Value Added Tax - VAT. https://www.skatteetaten.no/en/rates/value-added-tax/ (2021).

32. Eidgenössische Steuerverwaltung (ESTV). Was ist die Mehrwertsteuer. https://www.estv.admin.ch/estv/de/home/mehrwertsteuer/fachinformationen/was-ist-die-mehrwertsteuer.html (2021).

33. European Commission. VAT rates applied in the Member States of the European Union, Situation at 1st January 2020. (2020).

34. Bundesamt für Statistik. *Struktur der Arbeitskosten je geleistete Stunde (inklusive Lehrlinge), nach Wirtschaftsabschnitten (1/2). Construction (Section F), Arbeitskosten, CHF*. (2019).

35. Eidgenössische Steuerverwaltung (ESTV). Jahresmittelkurse 2019. https://www.estv.admin.ch/estv/de/home/direkte-bundessteuer/wehrpflichtersatzabgabe/dienstleistungen/jahresmittelkurse.html (2019).

36. Eurostat. *Labour cost levels by NACE Rev. 2 activity. Construction (Section F), Labour cost for LCI, €*. (2019).

37. Odyssee-Mure. Change in distance travelled by car. https://www.odyssee-mure.eu/publications/efficiency-by-sector/transport/distance-travelled-by-car.html.

38. Bundesamt für Statistik. *Leistungen des privaten Personenverkehrs auf der Strasse - Methodenbericht 2020 (Zeitreihe bis 2019)*. (2020).

39. Statistics Norway. Car numbers and mileage up. https://www.ssb.no/en/transport-og-reiseliv/artikler-og-publikasjoner/car-numbers-and-mileage-up (2021).

40. ACEA. Vehicles in use Europe, January 2021, ACEA Report. https://www.acea.be/uploads/publications/report-vehicles-in-use-europe-january-2021.pdf (2021).

41. Eurostat. Distribution of population by tenure status. (2020).

42. Eurostat. *Electricity prices components for household consumers. Consumption from 5 000 kWh to 14 999 kWh - band DD*. (2019).

43. Eurostat. *Electricity prices components for non-household consumers. Consumption 20 000 kWh to 499 000 kWh - band IB*. (2019).

44. Eurostat. Energy statistics - electricity prices for domestic and industrial consumers, price components - Reference Metadata in Euro SDMX Metadata Structure (ESMS). https://ec.europa.eu/eurostat/cache/metadata/en/nrg_pc_204_esms.htm (2021).

45. Eurostat. *Compilers guide on European statistics on natural gas and electricity prices*. (2017).

46. Bundesamt für Statistik. Regionalporträts 2020: Kennzahlen aller Gemeinden. https://www.bfs.admin.ch/bfs/de/home/statistiken/regionalstatistik/regionale-portraets-kennzahlen/gemeinden.assetdetail.11587763.html (2020).

47. Elcom. Basic data for tariffs of the Swiss Distribution Network Operators. https://www.elcom.admin.ch/elcom/en/home/topics/electricity-tariffs/basic-data-for-tariffs-of-the-swiss-distribution-network-operato.html (2021).

48. British Pound to Euro Spot Exchange Rates for 2019. https://www.exchangerates.org.uk/GBP-EUR-spot-exchange-rates-history-2019.html (2019).

49. Idaho National Laboratory (INL). Demand and Energy Characteristics of Non-Residential Alternating Current Level 2 Electric Vehicle Supply Equipment. (2015).

50. Neaimeh, M. *et al.* Analysing the usage and evidencing the importance of fast chargers for the adoption of battery electric vehicles. *Energy Policy* **108**, 474–486 (2017).

51. Amsterdam Roundtable Foundation / McKinsey & Company The Netherlands. *EVolution - Electric vehicles in Europe: gearing up for a new phase?*

https://www.mckinsey.com/~/media/McKinsey/Locations/Europe and Middle East/Netherlands/Our Insights/Electric vehicles in Europe Gearing up for a new phase/Electric vehicles in Europe Gearing up for a new phase.pdf (2014).

52. Lee, J. H., Chakraborty, D., Hardman, S. J. & Tal, G. Exploring electric vehicle charging patterns: Mixed usage of charging infrastructure. *Transp. Res. Part D Transp. Environ.* **79**, 102249 (2020).

53. Tal, G., Lee, J. H. & Nicholas, M. A. *Observed Charging Rates in California*. (2018).

54. Electric Power Research Institute (EPRI). Electric Vehicle Driving, Charging, and Load Shape Analysis - A Deep Dive Into Where, When, and How Much Salt River Project (SRP) Electric Vehicle Customers Charge. (2018).

55. Dena & Prognos. Privates Ladeinfrastrukturpotenzial in Deutschland. *Dtsch. Energie-Agentur GmbH* (2020).

56. Engel, H., Hensley, R., Knupfer, S. & Sahdev, S. Charging ahead: Electric-vehicle infrastructure demand. (2018).

57. Transport & Environment. *Recharge EU: how many charge points will Europe and its Member States need in the 2020s*. (2020).

58. Met Office. Cartopy: a cartographic python library with a Matplotlib interface. https://scitools.org.uk/cartopy.

59. Natural Earth. Free vector and raster map data.

60. Bloomberg. Gasoline Prices Around the World: The Real Cost of Filling Up. (2020).
