# Supplementary material for: Comparing the levelized cost of electric vehicle charging options in Europe
Source: Nat Commun. 2022 Sep 8;13:5277. doi: 10.1038/s41467-022-32835-7 (PMC9458728; doi:10.1038/s41467-022-32835-7)
Supplement: Supplementary file 3 — Description of Additional Supplementary Files [file 41467_2022_32835_MOESM3_ESM.pdf]

Title: Supplementary Dataset 1.
Description: This dataset includes all LCOC model input data organized by country and by charging option.

Title: Supplementary Dataset 2.
Description: This dataset includes all collected equipment costs for electric vehicle chargers of different power levels analyzed in the study.

Title: Supplementary Dataset 3.
Description: This dataset includes organized detailed results of the LCOC for all charging options in all countries, broken down into sub-components. The main LCOC results are included as well as the LCOC results for the four sensitivities performed in the analysis—electricity high, electricity low, medium time-of-use tariff reduction, high time-of-use tariff reduction.

Title: Supplementary Dataset 4.
Description: This dataset includes LCOC results for the five user profiles for all countries from the two sensitivities conducted to analyze electricity price sensitivity. The sheet Electricity price sensitivity includes LCOC results for the +/- 20 % electricity price sensitivity and compares them to the base case. The sheet TOU discount sensitivity includes LCOC results for the -20 % / -30 % TOU discount sensitivity and compares them to the base case.

Title: Supplementary Software 1.
Description: The Supplementary Software includes a zip file of the full Python model built and used in the study. All input data files, python scripts, and output data files are included and organized in this zip file. The zip file also includes a README.docx file with instructions on how to run the model.
