# Supplementary material for: Comparing the levelized cost of electric vehicle charging options in Europe
Source: Nat Commun. 2022 Sep 8;13:5277. doi: 10.1038/s41467-022-32835-7 (PMC9458728; doi:10.1038/s41467-022-32835-7)
Supplement: Supplementary file 8 — Software 1 [file 41467_2022_32835_MOESM8_ESM.zip › LCOC-Model/data/_other/ne_10m_admin_0_countries/ne_10m_admin_0_countries.README.html]

Admin 0 – Countries | Natural Earth


# 

Free vector and raster map data at 1:10m, 1:50m, and 1:110m scales

Search for:

- Home
- Features
- Downloads
- Blog
- Forums
- Corrections
- About

---

« 1:10m Cultural Vectors 
« Downloads

## Admin 0 – Countries

*There are 247 countries in the world. Greenland as separate from Denmark. Most users will want this file instead of sovereign states.*

Download countries (5.12 MB) version 4.0.0

Download without boundary lakes (5.26 MB) version 4.0.0

**About**

Countries distinguish between metropolitan (homeland) and independent and semi-independent portions of sovereign states. If you want to see the dependent overseas regions broken out (like in ISO codes, see France for example), use map units instead.

Each country is coded with a world region that roughly follows the United Nations setup.

Countries are coded with standard ISO and FIPS codes. French INSEE codes are also included.

Includes some thematic data from the United Nations (1), U.S. Central Intelligence Agency, and elsewhere.

**Disclaimer**

Natural Earth Vector draws boundaries of countries according to defacto status. We show who actually controls the situation on the ground. Please feel free to mashup our disputed area themes to match your particular political outlook.

**Known Problems**

None.

**Version History**

- 4.0.0
- 3.1.0
- 3.0.0
- 2.0.0
- 1.4.0
- 1.3.0
- 1.3
- 1.0.0

The master changelog is available on Github »

- ## Stay up to Date

  Know when a new version of Natural Earth is released by subscribing to our announcement list.

- ## Find a Problem?

  Submit suggestions and bug reports via our correction system and track the progress of your edits.

- ## Join Our Community

  Talk back and discuss Natural Earth in the Forums.

- ## Thank You

  Our data downloads are generously hosted by Florida State University.

- ## Recent Forum Topics

  - Natural Earth in Wagner VII
  - Downloads are 404ing
  - Disputed Territories: "type" field
  - ISO code confusion
  - Bad ADM1NAME, encoding in version 3.0.0 and missing diacritics in NAME
  - U.S. County Shape File
  - Projection / Proportion / Compatibility?
  - Download URLs – double slash
  - map soft – writer: me
  - Unicode encoding issue – ne\_10m\_lakes.dbf

- ## Forum Login

  Log in
  - or -
  Register

---

Supported by:

© 2009 - 2018. Natural Earth. All rights reserved.


Powered by WordPress

Staff Login »
