# Supplementary material for: Comparing the levelized cost of electric vehicle charging options in Europe
Source: Nat Commun. 2022 Sep 8;13:5277. doi: 10.1038/s41467-022-32835-7 (PMC9458728; doi:10.1038/s41467-022-32835-7)
Supplement: Supplementary file 8 — Software 1 [file 41467_2022_32835_MOESM8_ESM.zip › LCOC-Model/data/_other/Ninja-PV-Readme-v1.1.pdf]

The Renewables.ninja European PV data set – Version 1.1

Please cite these data and this document as:

> Stefan Pfenninger and Iain Staffell (2016). Long-term patterns of European PV output using 30 years of validated hourly reanalysis and satellite data. *Energy* 114, pp. 1251-1265. doi: 10.1016/j.energy.2016.08.060.

The data are stored in plain-text CSV format and contain 30 years of hourly capacity factors. All countries in the EU-28 plus Norway and Switzerland are included. There are two data files: one for MERRA-2 and one for CM-SAF SARAH simulations. MERRA-2 is better suited for long-term stability and overall consistency, SARAH for higher precision on hourly to daily time scales (but it suffers from some missing data). For details on these differences see the paper referenced above.

All times are given in UTC, with no shift for summer time / daylight savings time. When matching these capacity factors to other data sources, such as electricity demand from national system operators, you may need to shift the capacity factors to match the local time zone. Column headings are in the format of two letter ISO codes. Capacity factors are given in the range of [0, 1]. These can be multiplied by assumptions for installed capacity in each country to give the hourly power output from national PV fleets.

The most recent version of this data can always be found on www.renewables.ninja.

Country simulations

The *azimuth* and *tilt* columns give the mean value used for all sites simulated in the respective country.

Table 1: Aggregated metadata for simulated countries

ISO Code	Country name	Points	Azimuth	Tilt
**AL**	Albania	8	189.52	27.87
**AT**	Austria	31	177.77	31.64
**BA**	Bosnia and Herzegovina	18	178.35	29.27
**BE**	Belgium	11	191.70	24.17
**BG**	Bulgaria	39	195.81	25.61
**CH**	Switzerland	14	179.96	23.14
**CY**	Cyprus	3	180.00	35.00
**CZ**	Czech Republic	29	173.19	29.19
**DE**	Germany	135	179.50	30.94
**DK**	Denmark	21	166.63	36.26
**EE**	Estonia	22	177.50	31.99
**EL**	Greece	40	182.24	16.93
**ES**	Spain	157	178.35	24.36
**FI**	Finland	115	180.37	39.73
**FR**	France	197	178.39	28.44
**HR**	Croatia	17	184.79	25.08
**HU**	Hungary	34	186.91	28.37
**IE**	Ireland	28	174.23	33.47
**IT**	Italy	104	179.63	24.13
**LT**	Lithuania	27	180.95	30.54
**LU**	Luxembourg	2	207.33	28.06
**LV**	Latvia	31	160.76	32.49
**MD**	Moldova	10	172.55	23.27
**ME**	Montenegro	6	179.63	18.67
**MK**	Macedonia	8	192.79	28.78
**MT**	Malta	3	180.00	36.00
**NL**	Netherlands	14	170.71	30.62
**NO**	Norway	108	176.50	37.51
**PL**	Poland	122	180.43	32.81
**PT**	Portugal	27	177.53	23.66
**RO**	Romania	82	191.78	27.08
**RS**	Serbia	31	173.66	24.02
**SE**	Sweden	164	183.14	39.27
**SI**	Slovenia	7	191.52	21.59
**SK**	Slovakia	18	182.57	31.79
**UK**	United Kingdom	102	177.82	32.89

Version history

1.1 (June 2017)

- Extends v1.0 data to include 2015 and 2016 for MERRA-2, and 2015 for SARAH (the current version of SARAH does not extend beyond 2015)

1.0 (September 2016)

- 1985-2014 dataset described in Stefan Pfenninger and Iain Staffell (2016)

License

Creative Commons Attribution-NonCommercial 4.0 International

You are free to copy, redistribute and adapt the data for non-commercial purposes, provided you give appropriate credit. Note that the data is made available as-is and without warranty. We cannot guarantee its accuracy, and accept no responsibility for any liability arising from its use. You are advised to examine the quality of the data for your intended purposes, and to consult the publications linked on this page. If you wish to use the data for commercial purposes, please contact us.
