# Supplementary material for: Comparing the levelized cost of electric vehicle charging options in Europe
Source: Nat Commun. 2022 Sep 8;13:5277. doi: 10.1038/s41467-022-32835-7 (PMC9458728; doi:10.1038/s41467-022-32835-7)
Supplement: Supplementary file 8 — Software 1 [file 41467_2022_32835_MOESM8_ESM.zip › LCOC-Model/README.docx]

# **File structure**

- LCOC-Model
- data
  - LCOCData.xlsx (input data file)
  - _other (folder with additional data sources for map plotting and on PV output)
- model
  - .py (module scripts of the model)
  - LCOC_Model_environment.yml (anaconda environment)
- output
  - .xlsx and .pkl files computed by the model
    - files containing LCOC for 100 different utilization rates (LCOC_100_...)
    - files containing the same output for all scenarios (LCOC, LCOwoT, …)
    - files file containing all outputs for one scenario (Scenario_...)
    - files with the values of the sensitivity analysis (SensitivityAnalysis...)
    - files file containing LCOC of all aggregated user profiles for one scenario (UserProfile_[scenario])
    - files containing all outputs of one aggregated user profiles in base case (UserProfile_BaseScenario_[user profile])
  - __plots (folder with plots created by the model)

# **Define folder paths**

The following folder paths must be defined once at the beginning of file *externalParameters.py*:

- input folder (pointing to location of folder *data* in the LCOC model parent folder)
  r'…/LCOC-Model/data/'
- output folder (pointing to location of folder *output* in the LCOC model parent folder)
  r'…/LCOC-Model/output/'
- plots folder (pointing to location of folder *__plots* in the output folder)
  r'…/LCOC-Model/output/__plots/'
- other data folder (pointing to location of folder *_other* in the input folder)
  r'…/LCOC-Model/data/_other/'

Use / for MacOS and \ for Windows.

# **Run the model**

Once the folder paths are defined, import the anaconda environment (.yml file) for correct dependencies. Finally, to run the model, run *main.py*.

(for plotting, uncomment the desired lines at the end of *main.py*)
